# Glucocorticoids modulate gastrointestinal microbiome in a wild bird

**DOI:** 10.1098/rsos.171743

**Published:** 2018-04-18

**Authors:** José C. Noguera, Manuel Aira, Marcos Pérez-Losada, Jorge Domínguez, Alberto Velando

**Affiliations:** 1 Grupo de Ecología Animal, Universidade de Vigo, Torre CACTI, 36310, Vigo, Spain; 2 Computational Biology Institute, Milken Institute School of Public Health, George Washington University, Ashburn, VA, USA; 3 CIBIO-InBIO, Centro de Investigação em Biodiversidade e Recursos Genéticos, Universidade do Porto, Portugal

**Keywords:** gastrointestinal bacteria, hypothalamic–pituitary–adrenal axis, Illumina sequencing, *Larus michahellis*, pathogens

## Abstract

It has recently been hypothesized that stress exposure (e.g. via glucocorticoid secretion) may dysregulate the bacterial gut microbiome, a crucial ‘organ' in animal health. However, whether stress exposure (e.g. via glucocorticoid secretion) affects the bacterial gut microbiome of natural populations is unknown. We have experimentally altered the basal glucocorticoid level (corticosterone implants) in a wild avian species, the yellow-legged gull *Larus michahellis*, to assess its effects on the gastrointestinal microbiota. Our results suggest underrepresentation of several microbial taxa in the corticosterone-implanted birds. Importantly, such reduction included potentially pathogenic avian bacteria (e.g. *Mycoplasma* and *Microvirga*) and also some commensal taxa that may be beneficial for birds (e.g. Firmicutes). Our findings clearly demonstrate a close link between microbiome communities and glucocorticoid levels in natural populations. Furthermore, they suggest a beneficial effect of stress in reducing the risk of infection that should be explored in future studies.

## 1. Introduction

Most organisms have to cope with stressful events repeatedly across their life. In vertebrates, exposure to stressors triggers the hypothalamic–pituitary–adrenal (HPA) axis, a neuroendocrine pathway responsible for the production and release of stress hormones (i.e. glucocorticoids) (reviewed in [1]). The activation of the HPA axis has an inherent adaptive value, orchestrating a ‘fight or flight' response that promotes short-term survival [1,2]. Interestingly, it has long been recognized that the gut microbiome modulates stress responses and, particularly, the HPA axis (often referred to as ‘gut–brain axis' [3,4]). Surprisingly, although the relationship between the microbiome and the HPA axis is assumed to be bidirectional [3] and some previous studies in laboratory animals support it (e.g. [5,6]), whether stress responses affect the gut microbiome in natural populations has largely been overlooked.

Prolonged exposure to stressors may affect the susceptibility to infectious agents [7], as evidenced by some studies in birds and mammals [8,9]. However, whereas prolonged stress exposure may be damaging, the activation of the HPA axis may also enhance immune defences by reallocating leucocytes and macrophages towards more vulnerable tissues and organs (e.g. skin and gastrointestinal tracts [7,10,11]). The activation of the HPA axis may, therefore, be beneficial in the short term if, for instance, increased glucocorticoids reduce the load of the opportunistic pathogenic bacteria commonly found in the gut. Indeed, some recent studies suggest that glucocorticoids might reduce the proportion of some microbial taxa [12], and improve host resistance to some fungal, viral and bacterial pathogens [13,14].

Here, we examined the effect of stress hormones on the gut microbiome in free-living yellow-legged gull chicks via manipulating basal corticosterone levels. Corticosterone is the main stress hormone present in birds [15]. We experimentally elevated corticosterone levels within the natural range of variation via corticosterone implants and determined the effects on the gastrointestinal bacterial microbiome. We then assessed if the altered gut microbiota present in corticosterone-implanted chicks included potential pathogens and beneficial bacterial species.

## 2. Material and methods

### 2.1. Study area and field procedures

The field experiment was carried out between April and June 2016 in a colony of yellow-legged gulls in Sálvora Island, northwest Spain. All birds used in this experiment were part of a larger study [16]. We selected 64 three-egg nests (the modal clutch size in this species) with known laying date. Nests were identified and eggs were marked during laying. After clutch completion, we cross-fostered the whole clutch between nests with similar laying dates (±1 day), disrupting any potential parents–offspring microbiome covariation. Nests were then randomly assigned to ‘control' or ‘corticosterone' group. At hatching, chicks were marked for identification. The chick hatched from either the first- or the second-laid egg was randomly assigned to the experimental treatment, so we used only one chick per nest (see [16] for further details).

One day after hatching, the chicks were weighed (±1 g), blood sampled and surgically implanted between the shoulders with a 10-mm surgical silastic tube (Dow Corning; BB518-58). In the corticosterone group, implants were filled with crystallized corticosterone (Sigma-Aldrich; 27840), whereas in the control group, implants were empty (sham). At day 8 of age, 29 chicks (15 corticosterone-implanted and 14 sham-implanted) were trapped, blood sampled, weighed (±1 g) and placed on a clean plastic film until they defecated. Faeces were then harvested using a sterile microbiological swab (Copan, Italy), placed in sterile DNA/RNA free cryotubes (Simport, Canada) and stored in liquid nitrogen. Blood samples (day 1 and 8) were always collected within 3 min of capture to avoid any increase of baseline corticosterone levels as a consequence of handling [17]. Blood samples were kept cold until plasma was separated from red blood cells (within a few hours after collection) and stored in liquid nitrogen. Red blood cells (day 1) were used for molecular sexing of the chicks following [18], and plasma samples (day 1 and 8) to assess the variation in basal plasma corticosterone, triglycerides and protein levels. The sex ratio was similar between experimental groups (GLM with binomial error distribution; Wald *χ*^2 ^= 0.505, DF = 1, *p* = 0.477). A detailed description of the biochemical analyses (i.e. corticosterone, triglycerides and protein levels) and repeatability of the assays are provided in [16]. Importantly, the experimental treatment successfully increased basal corticosterone levels over the time of the experiment (from day 1 to 8 of age) and within the natural range of variation for the species (see [16] for further details and electronic supplementary material). Thus, our experimental manipulation had a significant effect on basal corticosterone levels; plasma corticosterone increased over time but significantly more in the corticosterone than in the control group of birds (treatment: *F*_1,28.33_ = 0.010, *p* = 0.922; age: *F*_1,13.93_ = 72.712, *p* < 0.001; treatment × age: *F*_1,13.96_ = 7.267, *p* = 0.017; electronic supplementary material, figure S1).

### 2.2. DNA extraction, amplification, sequencing and analysis of 16S rRNA genes

Bacterial DNA was extracted from faecal samples using commercial kits (Qiagen DNeasy kit) and following the manufacturer's instructions. We amplified and sequenced a fragment of the 16S rRNA gene covering the V4 region by using a dual-index sequencing strategy [19] and an Illumina MiSeq genome sequencer (Michigan Medical School). Sequence analysis was implemented using Mothur 1.36.1 [20], as detailed in Kozich *et al.* [19]. Briefly, we first combined forward and reverse reads for each sample and then removed sequences with ambiguous bases and longer than 275 base pairs. We then filtered off duplicate sequences and aligned the resulting fasta file using the SILVA reference alignment (version 123, as provided by www.mothur.org). Sequences were screened to overlap with the same alignment coordinates, filtered to remove columns without alignment data and then pre-clustered. Chimeras were checked with the ‘chimera.uchime' command in Mothur [21] and were then removed. Sequences were classified with the naive Bayesian classifier included in Mothur [22] by using the SILVA 123 database and any contaminants (sequences classified as mitochondria, chloroplast, archaea, eukaryote or not classified) were removed. To obtain operational taxonomic units (OTUs) at the 0.03 level, we first constructed a distance matrix (cut-off 0.20) and then clustered the resulting sequences into OTUs and classified them to obtain their consensus taxonomy. A total of 681 529 sequences (mean: 23 501, s.d.: 1125) passed all quality filters and were assigned to 2192 OTUs distributed in 33 bacterial phyla. Rarefaction curves indicated that the sampling depth was optimal for most samples (electronic supplementary material, figure S2). A large fraction of OTUs were singletons (770) and doubletons (330). We did a prevalence filtering of our dataset according to [23], setting a prevalence threshold of 5% of samples (electronic supplementary material, figure S3). This filtering eliminated all singletons and most of the doubletons, leaving a total of 732 OTUs distributed in 18 bacterial phyla.

### 2.3. Data analysis

We tested the effects of our corticosterone treatment on chick growth (body mass), basal corticosterone levels and triglyceride and protein content of chicks between 1 and 8 days of age using linear mixed models (LMMs). The models included treatment (control or corticosterone), age and sex as fixed factors. Two-way interaction between treatment and age was also tested. Chick identity was included as random terms in the models to account for the non-independence of measures from the same individual. In all models, we also explored the effect of hatching order. However, hatching order was never significant in our LMM analyses (*p* > 0.05) and, hence, was removed to avoid model over-parametrization. When needed, variables were log-transformed (i.e. triglycerides) or square root-transformed (i.e. corticosterone levels) to improve data distribution. In the model of chick growth, similar results were achieved when chick body mass was corrected for size (i.e. tarsus length; see electronic supplementary material, figure S1, for further details). Residuals obtained from the models were always normally distributed. We reported results from full models after removing non-significant interactions [24]. The Satterthwaite approximation was used for the estimation of degrees of freedom. Sample sizes among analyses can slightly differ because of the death or loss of chicks and/or insufficient volume of sample (for further details, see [16]). For illustrative purposes, data are presented as the change in values (day 8 minus day 1) and significant level was set as *p* = 0.05. Note that the birds used in this study are a subsample of those birds reported in [16] and, hence, the statistical models reported here do not correspond with any test published previously.

The R package phyloseq was used to import the sequence data [25]. For each sample, we calculated the taxonomic α diversity as the observed number of OTUs per individual (OTU richness), the estimated taxonomic richness (Chao1 richness) and Faith's phylogenetic diversity (PD), which were analysed by linear models (LMs) including treatment and sex as factors.

We used the package DESeq2 to perform differential OTU abundance between groups [26]. Briefly, the differential abundance and richness analyses in DESeq2 use a generalized linear model of counts following a negative binomial distribution, scaled by a normalization factor that accounts for differences in sequencing depth between samples. In the models, we included experimental treatment and sex as factors. Differential OTU abundances were assessed using the Wald tests and *p*-values adjusted by the false discovery rate (*p*-adj < 0.05). In all models, we also explored the effect of hatching order. However, hatching order was never significant in the models (*p*-adj > 0.05) and, hence, was removed to avoid model over-parametrization. Because our corticosterone treatment had a significant effect on OTU abundance (see Results), we also checked whether or not the OTUs that significantly differed between experimental groups (identified by DESeq2; see Results) included potentially pathogenic species previously described in the cloaca and faeces of wild birds [27,28].

We also normalized total count data (732 OTUs) by variance-stabilizing transformation as recommended in McMurdie & Holmes [26]. A principal component analysis (PCA) was performed on normalized data. Differences among samples in PC1 scores were analysed by a general linear model (lm in R) including treatment and sex as factors. We also performed a Bray–Curtis dissimilarity-based principal coordinate analysis (PCoA).

## 3. Results

**Figure 1.**
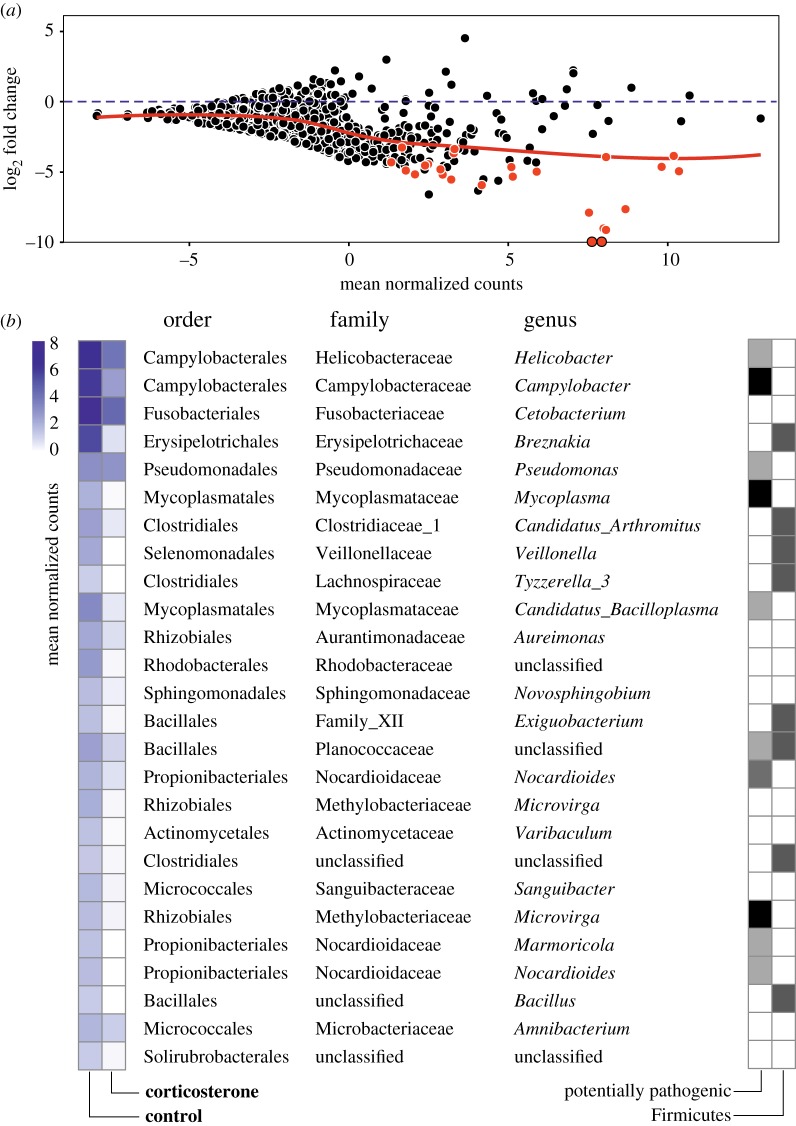
Effects of corticosterone implants on gastrointestinal microbiome of gull chicks. (*a*) The relationship between differential OTU representation (log_2_ fold change) and normalized abundance. Negative values indicate taxa underrepresented in corticosterone-implanted chicks. Red circles indicate taxa showing significant (*p*-adj < 0.05) differential abundance. Local polynomial regression (LOESS; red line) is shown. (*b*) Taxa showing significant differential abundance (DESeq2, *p*-adj < 0.05) between experimental groups (see electronic supplementary material, table S1, for further statistical details). Left heatplot shows normalized mean abundances in control and corticosterone chicks. Right heatplot shows OTUs belonging to potentially pathogenic genera (black) or classes (grey) and those belonging to the phylum Firmicutes.

Proteobacteria (33.65% sequences and 34% OTUs) and Firmicutes (48.61% sequences and 11.2% OTUs) dominated the gastrointestinal microbiome of gull chicks (see electronic supplementary material, figure S3). Corticosterone-implanted chicks tended to have lower α diversity, but the differences were not significant (OTU richness: *F*_1,26_ = 2.57, *p* = 0.12 ; Chao1: *F*_1,26_ = 2.16, *p* = 0.15; PD: *F*_1,26_ = 1.26, *p* = 0.27; see electronic supplementary material, figure S4). Our analyses revealed that most of the OTUs were underrepresented in corticosterone-implanted chicks compared with control ones, especially in those most abundant (figure 1*a*; electronic supplementary material, table S1). The mean of differences (mean: −1.78 log_2_ fold change; 95% CI: −1.93, −1.63) did not include the zero (*t *= 23.73; *p* < 0.001), suggesting an overall underrepresentation of several microbial taxa in corticosterone chicks.

In total, 26 individual OTUs showed significantly (*p*-adj < 0.05; see electronic supplementary material, table S1, for a detailed description of *p*-values for each individual OTU) different abundance between experimental treatments (figure 1*b*). In all cases, these OTUs were underrepresented in corticosterone-implanted chicks compared to controls (log_2_ fold change range: [−27.65, −3.26]). Several potential avian pathogenic OTUs (i.e. *Campylobacter*, *Mycoplasma*, *Microvirga, Helicobacter, Pseudomonas, Candidatus, Marmoricola* and *Nocardioides*) were underrepresented in corticosterone-implanted chicks (figure 1*b*). Some dominant taxa (e.g. 8 OTUs belonging to Firmicutes) were also underrepresented in corticosterone-implanted chicks (figure 1*b*). Male chicks showed a higher relative abundance of three OTUs than females (all *p*-adj < 0.05; see electronic supplementary material, table S2 and figure S5).

**Figure 2.**
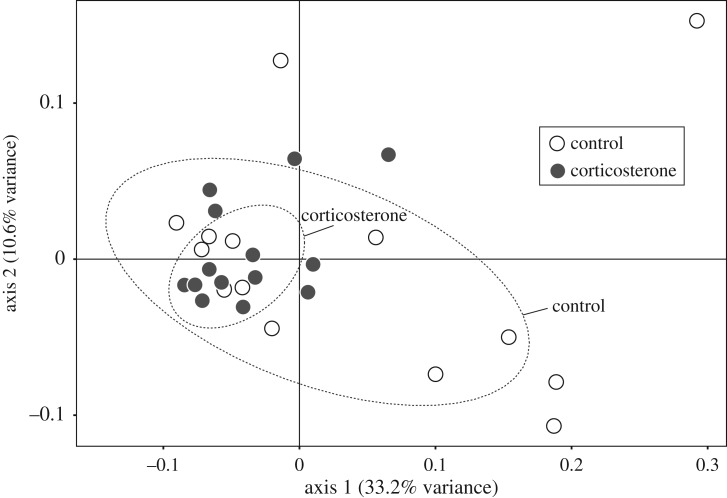
Principal coordinate analysis plot using Bray–Curtis distances on the normalized abundance of OTUs in control and corticosterone chicks. Ellipsoids represent standard errors assuming a multivariate *t*-distribution.

The first principal component (PC1) extracted from the normalized abundance of OTUs was a synoptic descriptor of overall OTU abundance, with 75% of the OTUs showing positive PC1 values. Corticosterone treatment had a significant effect on microbial communities as suggested by the PC1; corticosterone-implanted chicks had lower PC1 scores than control chicks (LM; *F*_1,26_ = 5.40, *p *= 0.028; electronic supplementary material, figure S6). Sex had no effect on PC1 scores (*F*_1,26_ = 1.17, *p *= 0.29). Moreover, PCoA also indicated that corticosterone chicks showed a narrower distribution in the coordinate space than control chicks, mostly due to significant differences in the first axis (axis 1: *F*_1,27_ = 5.51, *p* = 0.026; axis 2: *F*_1,27_ = 0.08, *p* = 0.78; figure 2).

Corticosterone-implanted chicks had lower growth rate than sham-implanted chicks (treatment: *F*_1,25.07_ = 5.140, *p* = 0.032; age: *F*_1,25.89_ = 231.30, *p* < 0.001; treatment × age: *F*_1,25.89_ = 5.051, *p* = 0.03; electronic supplementary material, figure S6). Neither plasma triglycerides nor protein levels differed between experimental groups of birds (triglycerides level: treatment: *F*_1,43_ = 2.325, *p* = 0.135; age: *F*_1,43_ = 0.055, *p* = 0.815; treatment×age: *F*_1,42_ = 0.003, *p* = 0.958; protein level: treatment: *F*_1,26.70_ = 0.556, *p* = 0 = 0.462; age: *F*_1,26.64_ = 2.399, *p* = 0.133; treatment × age: *F*_1,25.81_ = 1.623, *p* = 0.214).

## 4. Discussion

The microbiome of gull chicks was dominated by Proteobacteria and Firmicutes. Similar results were reported in previous studies, indicating that these two microbial phyla are the most abundant in seabird populations, including several gull species (see [29] and references therein). Our findings on differential abundance revealed that experimental elevation of corticosterone levels in wild gull chicks reduces the abundance of some members of the bacterial gastrointestinal microbiome. Our multivariate analyses (PCA and PCoA) also suggested that there was different microbial-community composition, with control chicks showing more dissimilarity among samples than corticosterone chicks.

From a mechanistic point of view, such variation in the microbiome of gull chicks could be caused by different, but not mutually exclusive, mechanisms. For instance, it has been recently shown that the avian gut microbiome is mostly environmentally acquired after hatching [30]. Hence, one possibility is that differences between experimental (i.e. corticosterone-implanted) and control chicks may result from changes in the amount of food and type of diet consumed [31], due to, for example, corticosterone effects on chick begging behaviour and/or parental provisioning [32,33]. Additionally, our corticosteroid treatment may have also favoured lipid accumulation through increased expression of fatty acid synthase [34], resulting in changes in the microbial-community composition leading to reductions in the proportions of some bacterial taxa [35]. However, the above two possibilities seem unlikely because neither plasma triglycerides nor protein levels (both important biomarkers of chicks’ nutritional status [16]) differed between experimental groups of birds in our experiment. A more likely explanation is that the observed reductions of bacterial species in corticosterone-implanted chicks have resulted from a rapid mobilization of leucocytes and other antimicrobial immune cells from peripheral circulating blood towards the gastrointestinal tract [10]. Moreover, higher corticosterone levels may have also caused the involution of lymphoid tissues (thymus, spleen or bursa of Fabricius; [36] and references therein), resulting in a glucocorticoid-mediated heterophilia that could have also contributed to further reducing the bacterial load in the corticosterone treatment group of birds.

It has been generally assumed that glucocorticoids increase the risk of proliferation of pathogenic bacteria [7]. However, our results do not support such hypothesis because some OTUs containing potential avian pathogens (e.g. *Campylobacter*, *Mycoplasma*, *Microvirga, Helicobacter, Pseudomonas, Candidatus, Marmoricola* or *Nocardioides*) were underrepresented in corticosterone-implanted chicks (figure 1*b*). Therefore, our results better support the hypothesis that activation of the HPA axis may reduce the vulnerability to opportunistic bacterial infections in the host organism [7,10]. Nonetheless, it is important to acknowledge that pathogenicity can vary among strains within the same bacterial genus and, therefore, more studies are needed to confirm the potential beneficial effect of stress responses in reducing infection risk in natural populations.

We also found sexual differences in some OTUs among chicks, including the most abundant OTU (*Catellicoccus*) and a genus containing potentially pathogenic species (*Proteus*). It is well established that sexes have different reproductive roles and selection favours sex-specific phenotypes [37] and their underlying molecular and physiological systems [38,39]. However, sex-specific differences in morphology, physiology and behaviour often appear even during early stages of postnatal development. Because the gut microbiome influences host development and physiology (e.g. organ development, morphogenesis, metabolism), future studies should investigate the extent to which sex-specific changes in the gut microbiome may be a driving factor underlying sex-specific phenotypical changes early in life.

Our analyses suggested that there was a reduction of some abundant bacteria in corticosterone-implanted chicks, including potentially beneficial bacteria from the phylum Firmicutes (figure 1*b*). Firmicutes has been related to the breakdown of complex biomolecules in vertebrates, facilitating their availability as an energy source to the host organism [40]. Thus, the reduction of such beneficial commensal bacteria in corticosterone-implanted chicks might involve a growth toll for the birds. Accordingly, we found that corticosterone chicks grew less than control chicks, supporting previous experimental bird studies where corticosterone-treated chicks typically show a reduced growth rate (e.g. [32,41,42]).

In conclusion, we have experimentally demonstrated that increased HPA axis activity in a free-living vertebrate resulted in a generalized reduction of some components of the gastrointestinal microbiome, including some OTUs that contain potential pathogenic species of bacteria in birds. The effect of the gut microbiome on stress responses has been recently established (i.e. gut–brain axis), but our results further highlight that the link is bidirectional, suggesting an important role of the neuroendocrine system in mediating host–microbiome coevolution.

## Supplementary Material

Table S1

## Supplementary Material

Table S2

## Supplementary Material

Supplemetary Materia

## Data accessibility

DNA sequences are available in GenBank SRA database under accession number SRP113238 and physiological data have been deposited in the Figshare digital repository (http://dx.doi.org/doi:10.6084/m9.figshare.5501587).
