# Supplementary material for: Glucocorticoids modulate gastrointestinal microbiome in a wild bird
Source: R Soc Open Sci. 2018 Apr 18;5(4):171743. doi: 10.1098/rsos.171743 (PMC5936907; doi:10.1098/rsos.171743)
Supplement: Table S1 [file rsos171743supp1.pdf]

Table S1. Summary of DESeq2 analyses for the effect of experimental treatment on differential OTUs abundance. Differential OTU abundances were assessed using the Wald tests and p-vales adjusted by false discovery rate. OTUs with a p-adj<0.05 are shown in bold.

| OTU | baseMean | log2FoldChange | lfcSE | stat | pvalue | padj | Phylum | Class | Order | Family | Genus |
|---|---|---|---|---|---|---|---|---|---|---|---|
| **Otu00051** | **243.6559783** | **-27.64822107** | **2.464672867** | **-11.21780559** | **3.33E-29** | **5.00E-27** | **Firmicutes** | **Negativicutes** | **Selenomonadales** | **Veillonellaceae** | **unclassified** |
| **Otu00046** | **196.9431252** | **-27.0890786** | **2.963456873** | **-9.141040266** | **6.19E-20** | **4.64E-18** | **Firmicutes** | **Clostridia** | **Clostridiales** | **Lachnospiraceae** | **Tyzzerella_3** |
| **Otu00018** | **408.5234806** | **-7.644840968** | **1.294826168** | **-5.904144631** | **3.54E-09** | **1.77E-07** | **Firmicutes** | **Erysipelotrichia** | **Erysipelotrichales** | **Erysipelotrichaceae** | **unclassified** |
| **Otu00007** | **895.9208519** | **-4.637471573** | **0.91949116** | **-5.043519475** | **4.57E-07** | **1.71E-05** | **Fusobacteria** | **Fusobacteriia** | **Fusobacteriales** | **Fusobacteriaceae** | **Cetobacterium** |
| **Otu00005** | **1306.372893** | **-4.949916186** | **1.076589884** | **-4.597773265** | **4.27E-06** | **0.000128109** | **Proteobacteria** | **Epsilonproteobacteria** | **Campylobacterales** | **Helicobacteraceae** | **Helicobacter** |
| **Otu00043** | **184.7994206** | **-7.893898997** | **1.855332092** | **-4.254709456** | **2.09E-05** | **0.000523302** | **Tenericutes** | **Mollicutes** | **Mycoplasmatales** | **Mycoplasmataceae** | **Candidatus_Bacilloplasma** |
| **Otu00036** | **253.338566** | **-9.019609651** | **2.726397796** | **-3.308251519** | **0.000938805** | **0.016151972** | **Firmicutes** | **Clostridia** | **Clostridiales** | **Clostridiaceae_1** | **Candidatus_Arthromitus** |
| **Otu00099** | **9.254444548** | **-5.540814722** | **1.679369721** | **-3.299341801** | **0.000969118** | **0.016151972** | **Proteobacteria** | **Alphaproteobacteria** | **Rhizobiales** | **Methylobacteriaceae** | **Microvirga** |
| **Otu00038** | **267.4782315** | **-9.123043166** | **2.764330737** | **-3.30027194** | **0.000965912** | **0.016151972** | **Tenericutes** | **Mollicutes** | **Mycoplasmatales** | **Mycoplasmataceae** | **unclassified** |
| **Otu00106** | **9.775271092** | **-3.667335792** | **1.134219954** | **-3.233355028** | **0.001223454** | **0.018351812** | **Actinobacteria** | **Actinobacteria** | **Propionibacteriales** | **Nocardioidaceae** | **Nocardioides** |
| **Otu00115** | **35.30162363** | **-5.334501049** | **1.679481915** | **-3.176277757** | **0.00149178** | **0.020342461** | **Proteobacteria** | **Alphaproteobacteria** | **Rhodobacterales** | **Rhodobacteraceae** | **unclassified** |
| **Otu00134** | **7.685948797** | **-5.187630777** | **1.646832306** | **-3.150066195** | **0.001632335** | **0.020404184** | **Actinobacteria** | **Actinobacteria** | **Actinomycetales** | **Actinomycetaceae** | **Varibaculum** |
| **Otu00144** | **4.211222821** | **-5.173176853** | **1.660374236** | **-3.115669192** | **0.00183528** | **0.021176311** | **Actinobacteria** | **Actinobacteria** | **Propionibacteriales** | **Nocardioidaceae** | **Marmoricola** |
| **Otu00112** | **5.594321984** | **-4.448835155** | **1.493624401** | **-2.978550131** | **0.002896156** | **0.031030248** | **Actinobacteria** | **Actinobacteria** | **Micrococcales** | **Sanguibacteraceae** | **Sanguibacter** |
| **Otu00182** | **3.490886982** | **-4.911945617** | **1.686078226** | **-2.913237086** | **0.003577029** | **0.03401952** | **Actinobacteria** | **Actinobacteria** | **Propionibacteriales** | **Nocardioidaceae** | **Nocardioides** |
| **Otu00155** | **7.322988817** | **-4.815189059** | **1.655414237** | **-2.908751751** | **0.003628749** | **0.03401952** | **Firmicutes** | **Clostridia** | **Clostridiales** | **unclassified** | **unclassified** |
| **Otu00058** | **267.8344798** | **-3.941186511** | **1.370990708** | **-2.874699653** | **0.00404412** | **0.035683411** | **Proteobacteria** | **Gammaproteobacteria** | **Pseudomonadales** | **Pseudomonadaceae** | **Pseudomonas** |
| **Otu00010** | **1166.184605** | **-3.860141385** | **1.394216424** | **-2.768681619** | **0.005628361** | **0.038375186** | **Proteobacteria** | **Epsilonproteobacteria** | **Campylobacterales** | **Campylobacteraceae** | **Campylobacter** |
| **Otu00139** | **5.24282447** | **-4.538538938** | **1.613472681** | **-2.812901012** | **0.004909676** | **0.038375186** | **Proteobacteria** | **Alphaproteobacteria** | **Rhizobiales** | **Methylobacteriaceae** | **Microvirga** |
| **Otu00071** | **59.30776908** | **-4.980507824** | **1.758801962** | **-2.83176158** | **0.004629235** | **0.038375186** | **Proteobacteria** | **Alphaproteobacteria** | **Rhizobiales** | **Aurantimonadaceae** | **unclassified** |
| **Otu00113** | **17.96886098** | **-5.93479391** | **2.133870494** | **-2.781234346** | **0.005415263** | **0.038375186** | **Firmicutes** | **Bacilli** | **Bacillales** | **Family_XII** | **Exiguobacterium** |
| **Otu00087** | **9.943118465** | **-3.371646171** | **1.205721811** | **-2.796371551** | **0.005167995** | **0.038375186** | **Firmicutes** | **Bacilli** | **Bacillales** | **Planococcaceae** | **unclassified** |
| **Otu00209** | **3.450249548** | **-4.900954032** | **1.792906626** | **-2.733524413** | **0.006266048** | **0.040865529** | **Firmicutes** | **Bacilli** | **Bacillales** | **unclassified** | **unclassified** |
| **Otu00189** | **2.511716869** | **-4.313677756** | **1.59123524** | **-2.710898834** | **0.00671011** | **0.041938185** | **Actinobacteria** | **Thermoleophilia** | **Solirubrobacterales** | **unclassified** | **unclassified** |
| **Otu00049** | **3.172354417** | **-3.261322649** | **1.235861816** | **-2.638905585** | **0.008317414** | **0.047985082** | **Actinobacteria** | **Actinobacteria** | **Micrococcales** | **Microbacteriaceae** | **Amnibacterium** |
| **Otu00135** | **34.19944432** | **-4.652226551** | **1.757624897** | **-2.646882482** | **0.008123758** | **0.047985082** | **Proteobacteria** | **Alphaproteobacteria** | **Sphingomonadales** | **Sphingomonadaceae** | **unclassified** |
| Otu00045 | 48.54878085 | -4.205204396 | 1.638595698 | -2.566346537 | 0.010277609 | 0.057097829 | Firmicutes | Bacilli | Lactobacillales | Streptococcaceae | Streptococcus |
| Otu00109 | 32.90172373 | -4.152552799 | 1.642672798 | -2.527924493 | 0.011473903 | 0.061467335 | Firmicutes | Bacilli | Bacillales | Planococcaceae | unclassified |
| Otu00129 | 18.62653038 | -5.520241105 | 2.206778075 | -2.501493543 | 0.01236707 | 0.063967602 | Firmicutes | Clostridia | Clostridiales | Family_XI | Parvimonas |
| Otu00082 | 15.15731967 | -3.542418865 | 1.458043107 | -2.429570737 | 0.015116715 | 0.075583574 | Actinobacteria | Thermoleophilia | Solirubrobacterales | Solirubrobacteraceae | Solirubrobacter |
| Otu00154 | 4.63727302 | -3.98849616 | 1.678319688 | -2.37648178 | 0.017478625 | 0.081931053 | Actinobacteria | Actinobacteria | Micrococcales | Micrococcaceae | unclassified |
| Otu00081 | 15.36432648 | -3.542977658 | 1.487275725 | -2.38219289 | 0.01720988 | 0.081931053 | Actinobacteria | Actinobacteria | Propionibacteriales | Nocardioidaceae | Nocardioides |
| Otu00095 | 8.587591049 | -3.900092485 | 1.664110845 | -2.343649461 | 0.019096109 | 0.084561353 | Proteobacteria | Alphaproteobacteria | Rhizobiales | Hyphomicrobiaceae | Devosia |
| Otu00110 | 5.693446855 | -6.599988223 | 2.817783593 | -2.34226228 | 0.01916724 | 0.084561353 | Firmicutes | Bacilli | Bacillales | Planococcaceae | unclassified |
| Otu00093 | 9.442991464 | -3.527524463 | 1.545484349 | -2.282471813 | 0.0224615 | 0.096263571 | Actinobacteria | Actinobacteria | Micrococcales | Micrococcaceae | Arthrobacter |
| Otu00076 | 12.4791655 | 4.508935672 | 1.995970693 | 2.259018977 | 0.023882204 | 0.096819748 | Proteobacteria | Gammaproteobacteria | Pseudomonadales | Moraxellaceae | Acinetobacter |
| Otu00034 | 30.89457494 | -2.576435502 | 1.140349721 | -2.259338039 | 0.023862365 | 0.096819748 | Proteobacteria | Alphaproteobacteria | Rhizobiales | unclassified | unclassified |
| Otu00235 | 4.207574809 | -4.102876128 | 1.833577521 | -2.237634396 | 0.025244907 | 0.099650947 | Proteobacteria | Alphaproteobacteria | Rhizobiales | unclassified | unclassified |
| Otu00104 | 12.3255541 | -3.493289972 | 1.641295299 | -2.128373837 | 0.033306101 | 0.119974191 | Actinobacteria | Actinobacteria | Micrococcales | Micrococcaceae | unclassified |
| Otu00136 | 6.228095345 | -3.105614314 | 1.450775031 | -2.140658784 | 0.032301565 | 0.119974191 | Proteobacteria | Alphaproteobacteria | Rhizobiales | Aurantimonadaceae | unclassified |
| Otu00247 | 1.857864702 | -2.88914162 | 1.359643291 | -2.124926176 | 0.033592773 | 0.119974191 | Proteobacteria | Alphaproteobacteria | Rhodobacterales | Rhodobacteraceae | unclassified |
| Otu00149 | 16.60101205 | -6.328896458 | 2.938573285 | -2.153731026 | 0.031261272 | 0.119974191 | Verrucomicrobia | Verrucomicrobiae | Verrucomicrobiales | Verrucomicrobiaceae | unclassified |
| Otu00084 | 12.75940146 | -2.373562565 | 1.131658711 | -2.097419073 | 0.035956494 | 0.125429631 | Actinobacteria | Actinobacteria | Micrococcales | Microbacteriaceae | unclassified |
| Otu00097 | 11.39696283 | -2.828371887 | 1.356482217 | -2.085078486 | 0.037062177 | 0.12634833 | Actinobacteria | Actinobacteria | Micrococcales | Cellulomonadaceae | Cellulomonas |
| Otu00133 | 5.880239963 | -3.351502337 | 1.625674322 | -2.061607476 | 0.039245122 | 0.130817072 | Actinobacteria | Thermoleophilia | Solirubrobacterales | Solirubrobacteraceae | Solirubrobacter |
| Otu00161 | 3.38538582 | -3.62821604 | 1.768328444 | -2.051777232 | 0.040191317 | 0.131058642 | Actinobacteria | Actinobacteria | Propionibacteriales | Nocardioidaceae | Nocardioides |
| Otu00059 | 14.35979803 | -2.351614909 | 1.174806082 | -2.001704746 | 0.045316496 | 0.143201853 | Actinobacteria | Actinobacteria | Corynebacteriales | Mycobacteriaceae | Mycobacterium |
| Otu00103 | 10.79486826 | -3.35387612 | 1.679452677 | -1.997005432 | 0.045824593 | 0.143201853 | Actinobacteria | Actinobacteria | Frankiales | Geodermatophilaceae | unclassified |
| Otu00162 | 1.949040728 | -3.344160474 | 1.682523516 | -1.987586172 | 0.046857477 | 0.143441256 | Actinobacteria | Actinobacteria | Pseudonocardiales | Pseudonocardiaceae | Actinomycetospora |
| Otu00132 | 5.156637352 | -2.931780433 | 1.504430814 | -1.948763881 | 0.051323629 | 0.153970887 | Proteobacteria | Alphaproteobacteria | Rhizobiales | Methylobacteriaceae | Methylobacterium |
| Otu00179 | 3.810222184 | -3.979772751 | 2.07615996 | -1.916891197 | 0.055251756 | 0.159380064 | Proteobacteria | Alphaproteobacteria | Caulobacterales | Caulobacteraceae | Brevundimonas |
| Otu00074 | 25.73978137 | -5.623632566 | 2.926983204 | -1.921306743 | 0.054693048 | 0.159380064 | Bacteroidetes | Sphingobacteriia | Sphingobacteriales | Sphingobacteriaceae | unclassified |
| Otu00183 | 7.840505283 | -4.557772118 | 2.405688489 | -1.894581173 | 0.058147911 | 0.163313916 | Actinobacteria | Actinobacteria | Corynebacteriales | Nocardiaceae | Rhodococcus |
| Otu00142 | 9.870763988 | -4.220930645 | 2.233606275 | -1.889737996 | 0.05879301 | 0.163313916 | Cyanobacteria | Cyanobacteria | SubsectionIII | FamilyI | unclassified |
| Otu00158 | 4.724354716 | -3.995251064 | 2.171898425 | -1.839520218 | 0.065838707 | 0.178737542 | Proteobacteria | Alphaproteobacteria | Rhizobiales | Bradyrhizobiaceae | unclassified |
| Otu00120 | 3.849632301 | -2.463889129 | 1.3438195 | -1.833497079 | 0.066728683 | 0.178737542 | Proteobacteria | Alphaproteobacteria | Rhizobiales | Bradyrhizobiaceae | unclassified |
| Otu00021 | 131.6752215 | 2.01829371 | 1.121211525 | 1.800100752 | 0.071844731 | 0.189065081 | Firmicutes | Bacilli | Lactobacillales | Enterococcaceae | Enterococcus |
| Otu00178 | 2.833792985 | -2.701352093 | 1.515836304 | -1.782086948 | 0.074735054 | 0.193280312 | Actinobacteria | Actinobacteria | Propionibacteriales | Nocardioidaceae | Aeromicrobium |
| Otu00140 | 3.823014991 | -3.052908316 | 1.73620845 | -1.758376603 | 0.078683451 | 0.198604533 | Actinobacteria | Actinobacteria | Micrococcales | Microbacteriaceae | Curtobacterium |
| Otu00206 | 2.028827699 | -3.930433746 | 2.240924515 | -1.753934021 | 0.079441813 | 0.198604533 | Actinobacteria | Actinobacteria | Propionibacteriales | Nocardioidaceae | Marmoricola |
| Otu00124 | 3.329059964 | -2.434379515 | 1.40534583 | -1.732228084 | 0.083232961 | 0.200707944 | Actinobacteria | Actinobacteria | Micromonosporales | Micromonosporaceae | unclassified |
| Otu00114 | 5.07338925 | -2.927330523 | 1.687843447 | -1.7343614 | 0.082853979 | 0.200707944 | Actinobacteria | Actinobacteria | Pseudonocardiales | Pseudonocardiaceae | Pseudonocardia |
| Otu00008 | 51.03832308 | -3.271424259 | 1.919238927 | -1.704542468 | 0.088279789 | 0.200707944 | Proteobacteria | Epsilonproteobacteria | Campylobacterales | Campylobacteraceae | Campylobacter |
| Otu00186 | 1.940720046 | -3.127964269 | 1.823126498 | -1.715714336 | 0.08621434 | 0.200707944 | Proteobacteria | Alphaproteobacteria | Rhizobiales | Bradyrhizobiaceae | Bosea |
| Otu00001 | 7711.896819 | -1.199375757 | 0.70370513 | -1.704372621 | 0.088311496 | 0.200707944 | Firmicutes | Bacilli | Lactobacillales | Enterococcaceae | Catellicoccus |
| Otu00068 | 4.41495154 | -2.541424932 | 1.485458896 | -1.710868566 | 0.087105379 | 0.200707944 | Tenericutes | Mollicutes | Mycoplasmatales | Mycoplasmataceae | Ureaplasma |
| Otu00105 | 8.405437787 | -2.24922838 | 1.353379978 | -1.661934132 | 0.096525982 | 0.213197267 | Actinobacteria | Actinobacteria | Frankiales | Geodermatophilaceae | Blastococcus |
| Otu00090 | 6.827741334 | -2.722462495 | 1.638735698 | -1.661318843 | 0.096649428 | 0.213197267 | Proteobacteria | Alphaproteobacteria | Rhizobiales | Methylobacteriaceae | unclassified |
| Otu00141 | 3.87744812 | -2.755898159 | 1.665923716 | -1.654276323 | 0.098071382 | 0.213198655 | Proteobacteria | Alphaproteobacteria | Rhizobiales | Rhizobiaceae | Rhizobium |
| Otu00148 | 5.196405374 | -4.828792495 | 2.942832626 | -1.640865489 | 0.10082534 | 0.216054301 | Proteobacteria | Alphaproteobacteria | Sphingomonadales | Erythrobacteraceae | Altererythrobacter |
| Otu00177 | 2.344313097 | -2.231515681 | 1.3703575 | -1.628418629 | 0.10343615 | 0.218418656 | Actinobacteria | Actinobacteria | Micrococcales | Microbacteriaceae | unclassified |
| Otu00252 | 2.484997387 | -4.280105178 | 2.64517259 | -1.618081631 | 0.105645007 | 0.218418656 | Proteobacteria | Alphaproteobacteria | Rhodospirillales | Rhodospirillaceae | Skermanella |
| Otu00314 | 3.032871066 | -4.458876107 | 2.760806513 | -1.615062876 | 0.106297079 | 0.218418656 | Firmicutes | Bacilli | Bacillales | Paenibacillaceae | unclassified |
| Otu00259 | 3.370576744 | -4.521304126 | 2.81126951 | -1.608278435 | 0.107774212 | 0.21846124 | Cyanobacteria | unclassified | unclassified | unclassified | unclassified |
| Otu00023 | 200.9623519 | -2.292561981 | 1.442017784 | -1.5898292 | 0.11187331 | 0.219945885 | Proteobacteria | Gammaproteobacteria | Vibrionales | Vibrionaceae | Vibrio |
| Otu00211 | 8.462411493 | -4.658835789 | 2.938832459 | -1.585267569 | 0.112905554 | 0.219945885 | Proteobacteria | Alphaproteobacteria | Sphingomonadales | unclassified | unclassified |
| Otu00331 | 3.40126558 | -4.667484423 | 2.943879444 | -1.585487623 | 0.112855587 | 0.219945885 | Chloroflexi | TK10 | unclassified | unclassified | unclassified |
| Otu00039 | 12.00729958 | -2.890541298 | 1.860294025 | -1.553808838 | 0.120230022 | 0.23121158 | Firmicutes | Clostridia | Clostridiales | Peptostreptococcaceae | Peptostreptococcus |
| Otu00004 | 1368.475293 | -1.39490011 | 0.912698113 | -1.52832584 | 0.126431657 | 0.240060109 | Firmicutes | Clostridia | Clostridiales | Clostridiaceae_1 | Clostridium_sensu_stricto_1 |
| Otu00350 | 2.260551668 | -4.429135855 | 2.926041298 | -1.513695606 | 0.13010306 | 0.240544887 | Actinobacteria | Thermoleophilia | Gaiellales | Gaiellaceae | Gaiella |
| Otu00088 | 5.915171257 | -2.038090528 | 1.352687312 | -1.506697454 | 0.131888225 | 0.240544887 | Actinobacteria | Actinobacteria | Pseudonocardiales | Pseudonocardiaceae | Pseudonocardia |
| Otu00015 | 131.2317671 | 2.226862176 | 1.482614785 | 1.501982982 | 0.133101504 | 0.240544887 | Proteobacteria | Gammaproteobacteria | Enterobacteriales | Enterobacteriaceae | Edwardsiella |
| Otu00127 | 6.038200836 | -2.872841131 | 1.908532208 | -1.505262064 | 0.132256714 | 0.240544887 | Proteobacteria | Betaproteobacteria | Burkholderiales | Comamonadaceae | unclassified |
| Otu00057 | 58.54481543 | -4.322423296 | 2.910825013 | -1.484947833 | 0.137557644 | 0.245638649 | Bacteroidetes | Flavobacteriia | Flavobacteriales | Flavobacteriaceae | Chryseobacterium |
| Otu00300 | 3.368513767 | -4.335433277 | 2.945460406 | -1.471903431 | 0.141046957 | 0.248906395 | Fusobacteria | Fusobacteriia | Fusobacteriales | Fusobacteriaceae | Fusobacterium |
| Otu00379 | 2.287288421 | -4.289533877 | 2.945841195 | -1.456132084 | 0.14535611 | 0.253528099 | Proteobacteria | Alphaproteobacteria | Rhodobacterales | Rhodobacteraceae | Rubellimicrobium |
| Otu00242 | 1.971839732 | -3.202411685 | 2.217733942 | -1.444001746 | 0.148738484 | 0.253531507 | Actinobacteria | Actinobacteria | Frankiales | unclassified | unclassified |
| Otu00073 | 5.730592762 | -4.259505555 | 2.942759008 | -1.447453068 | 0.147770072 | 0.253531507 | Firmicutes | Negativicutes | Selenomonadales | Veillonellaceae | unclassified |
| Otu00100 | 2.340740749 | -2.487253051 | 1.759375413 | -1.413713658 | 0.157445994 | 0.260088623 | Proteobacteria | Alphaproteobacteria | Rhizobiales | Phyllobacteriaceae | unclassified |
| Otu00399 | 2.526123286 | -4.163239633 | 2.946706951 | -1.412844814 | 0.157701358 | 0.260088623 | Firmicutes | Bacilli | Bacillales | Bacillaceae | Bacillus |
| Otu00031 | 3.146333301 | -4.158348303 | 2.94385224 | -1.412553336 | 0.157787098 | 0.260088623 | Bacteroidetes | Bacteroidia | Bacteroidales | Prevotellaceae | Alloprevotella |
| Otu00126 | 8.641083757 | -3.82669156 | 2.755584109 | -1.388704321 | 0.16492268 | 0.268895673 | Bacteroidetes | Sphingobacteriia | Sphingobacteriales | Sphingobacteriaceae | Pedobacter |
| Otu00167 | 3.622541416 | -3.430380721 | 2.496029674 | -1.374334911 | 0.169337733 | 0.273125375 | Proteobacteria | Alphaproteobacteria | Caulobacterales | Caulobacteraceae | Brevundimonas |
| Otu00130 | 3.769428884 | -2.672355514 | 1.968290386 | -1.35770389 | 0.174557662 | 0.278549461 | Actinobacteria | Actinobacteria | Propionibacteriales | Nocardioidaceae | Nocardioides |
| Otu00298 | 2.20634243 | -3.888392938 | 2.900562466 | -1.340565143 | 0.180061681 | 0.281346376 | Actinobacteria | Actinobacteria | Micrococcales | Micrococcaceae | Arthrobacter |
| Otu00358 | 2.725124164 | -3.946503538 | 2.943409891 | -1.340793054 | 0.179987652 | 0.281346376 | Bacteroidetes | Cytophagia | Cytophagales | Cytophagaceae | Hymenobacter |
| Otu00096 | 6.028315176 | -2.230480481 | 1.686037726 | -1.322912558 | 0.185864459 | 0.286368399 | Actinobacteria | Actinobacteria | Micrococcales | Micrococcaceae | Rothia |
| Otu00214 | 2.338051656 | -3.269655597 | 2.489205553 | -1.313533787 | 0.189003144 | 0.286368399 | Firmicutes | Clostridia | Clostridiales | Peptostreptococcaceae | unclassified |
| Otu00098 | 2.27469717 | -2.043795934 | 1.555369193 | -1.314026241 | 0.188837374 | 0.286368399 | Proteobacteria | Alphaproteobacteria | Rhizobiales | Xanthobacteraceae | Variibacter |
| Otu00296 | 2.399588329 | -3.235950466 | 2.500766393 | -1.293983507 | 0.195671118 | 0.287751644 | Proteobacteria | Alphaproteobacteria | Rhizobiales | Methylobacteriaceae | Methylobacterium |
| Otu00020 | 66.76096931 | -1.96266657 | 1.510724085 | -1.299156206 | 0.193890328 | 0.287751644 | Proteobacteria | Alphaproteobacteria | Rhodobacterales | Rhodobacteraceae | Paracoccus |
| Otu00191 | 2.262385967 | -3.429493773 | 2.649013616 | -1.294630482 | 0.195447732 | 0.287751644 | Firmicutes | Bacilli | Lactobacillales | Aerococcaceae | unclassified |
| Otu00044 | 26.03633665 | -2.24067347 | 1.756623876 | -1.275556766 | 0.202112245 | 0.294338222 | Firmicutes | Clostridia | Clostridiales | Peptostreptococcaceae | Peptostreptococcus |
| Otu00072 | 29.0026344 | -2.323365325 | 1.879933591 | -1.235876276 | 0.216504557 | 0.297942051 | Firmicutes | Clostridia | Clostridiales | Peptostreptococcaceae | Peptostreptococcus |
| Otu00032 | 4.636276263 | -1.743960875 | 1.397583264 | -1.247840411 | 0.212089506 | 0.297942051 | Firmicutes | Clostridia | Clostridiales | Clostridiaceae_1 | Clostridium_sensu_stricto_1 |
| Otu00121 | 6.922869835 | -1.924940348 | 1.554724518 | -1.238123105 | 0.215670418 | 0.297942051 | Proteobacteria | Betaproteobacteria | Burkholderiales | Comamonadaceae | unclassified |
| Otu00208 | 2.127401634 | -2.539990009 | 2.049587554 | -1.239268849 | 0.215245951 | 0.297942051 | Proteobacteria | Alphaproteobacteria | Rhizobiales | unclassified | unclassified |
| Otu00152 | 2.519596275 | -2.218493159 | 1.786292623 | -1.241953939 | 0.214253557 | 0.297942051 | Firmicutes | Bacilli | Bacillales | Bacillaceae | unclassified |
| Otu00185 | 3.881221484 | -2.628016787 | 2.107061344 | -1.247242656 | 0.212308537 | 0.297942051 | Bacteroidetes | Sphingobacteriia | Sphingobacteriales | Sphingobacteriaceae | unclassified |
| Otu00150 | 3.639395038 | -1.786655227 | 1.483105742 | -1.204671505 | 0.22833014 | 0.310905399 | Actinobacteria | Actinobacteria | Micromonosporales | Micromonosporaceae | Actinoplanes |
| Otu00117 | 8.780392089 | -3.503284176 | 2.918969106 | -1.200178573 | 0.230069995 | 0.310905399 | Bacteroidetes | Sphingobacteriia | Sphingobacteriales | Sphingobacteriaceae | Pedobacter |
| Otu00079 | 8.97091848 | -1.439289363 | 1.253499258 | -1.148217164 | 0.250878925 | 0.335998561 | Proteobacteria | Alphaproteobacteria | Rhizobiales | Methylobacteriaceae | Methylobacterium |
| Otu00278 | 1.923786512 | -3.367463772 | 2.950805404 | -1.141201574 | 0.253786049 | 0.336884136 | Proteobacteria | Alphaproteobacteria | Sphingomonadales | unclassified | unclassified |
| Otu00156 | 2.659222955 | -3.308497036 | 2.929853722 | -1.129236252 | 0.258798185 | 0.340523927 | Firmicutes | Bacilli | Lactobacillales | Lactobacillaceae | Lactobacillus |

| | | | | | | | | | | | | |
|---|---|---|---|---|---|---|---|---|---|---|---|---|
| Otu00003 | 464.568075 | 0.988178856 | 0.921170958 | 1.072742087 | 0.283386851 | 0.369635023 | Fusobacteria | Fusobacteriia | Fusobacteriales | Fusobacteriaceae | Fusobacterium |
| Otu00223 | 1.886934395 | -2.397037557 | 2.251154778 | -1.064803531 | 0.286964847 | 0.370333551 | Actinobacteria | Actinobacteria | Micrococcales | Intrasporangiaceae | Janibacter |
| Otu00262 | 2.165985971 | -2.399019854 | 2.261891738 | -1.060625411 | 0.28886017 | 0.370333551 | Chloroflexi | Thermomicrobia | JG30-KF-CM45 | unclassified | unclassified |
| Otu00086 | 15.08138773 | -2.019686612 | 1.931115954 | -1.045865013 | 0.29562336 | 0.375792406 | Proteobacteria | Alphaproteobacteria | Sphingomonadales | Sphingomonadaceae | Sphingomonas |
| Otu00060 | 2.262380845 | 2.983801107 | 2.935901909 | 1.016314986 | 0.309479415 | 0.390100102 | Proteobacteria | Gammaproteobacteria | Pseudomonadales | Moraxellaceae | Moraxella |
| Otu00016 | 8.229295065 | 2.128440884 | 2.215591416 | 0.96066489 | 0.33672069 | 0.420900863 | Proteobacteria | Gammaproteobacteria | Pasteurellales | Pasteurellaceae | unclassified |
| Otu00024 | 85.9760934 | -1.033262618 | 1.095275621 | -0.943381372 | 0.345485868 | 0.424777706 | Firmicutes | Bacilli | Lactobacillales | Lactobacillaceae | Lactobacillus |
| Otu00159 | 3.661596775 | -1.822957816 | 1.927639651 | -0.945694293 | 0.344304534 | 0.424777706 | Firmicutes | Bacilli | Bacillales | unclassified | unclassified |
| Otu00012 | 282.3827 | -1.375058521 | 1.53303762 | -0.896950279 | 0.369745449 | 0.450909084 | Actinobacteria | Actinobacteria | Corynebacteriales | Corynebacteriaceae | unclassified |
| Otu00019 | 1.963523867 | -2.513525116 | 2.935499963 | -0.856251115 | 0.391858895 | 0.474022857 | Proteobacteria | Epsilonproteobacteria | Campylobacterales | Helicobacteraceae | Helicobacter |
| Otu00022 | 114.0967224 | 0.87751793 | 1.046941104 | 0.838173157 | 0.401933461 | 0.482320153 | Firmicutes | Bacilli | Lactobacillales | Enterococcaceae | Enterococcus |
| Otu00193 | 2.985930733 | -1.892355831 | 2.335224991 | -0.81035268 | 0.417737506 | 0.497306555 | Actinobacteria | Actinobacteria | Micromonosporales | Micromonosporaceae | unclassified |
| Otu00083 | 9.29662804 | 1.200782033 | 1.591927798 | 0.75429428 | 0.450672536 | 0.532290397 | Proteobacteria | Gammaproteobacteria | Pseudomonadales | Moraxellaceae | Acinetobacter |
| Otu00063 | 2.951050171 | -2.148658474 | 2.932412061 | -0.732727335 | 0.463724749 | 0.54342744 | Proteobacteria | Gammaproteobacteria | Pseudomonadales | Moraxellaceae | Psychrobacter |
| Otu00147 | 5.968259234 | -1.058397823 | 1.643118784 | -0.644139568 | 0.519484934 | 0.594591688 | Actinobacteria | Actinobacteria | Frankiales | Nakamurellaceae | Nakamurella |
| Otu00062 | 24.79909815 | -0.819801377 | 1.279942369 | -0.640498664 | 0.521848458 | 0.594591688 | Actinobacteria | Actinobacteria | Micromonosporales | Micromonosporaceae | unclassified |
| Otu00187 | 3.368174125 | -1.670611987 | 2.617045719 | -0.638357968 | 0.523240685 | 0.594591688 | Proteobacteria | Alphaproteobacteria | Rhizobiales | Methylobacteriaceae | Methylobacterium |
| Otu00092 | 14.09880287 | -1.896442338 | 2.940662307 | -0.644903134 | 0.51898996 | 0.594591688 | Bacteroidetes | Flavobacteriia | Flavobacteriales | Flavobacteriaceae | unclassified |
| Otu00002 | 1645.206147 | 0.434363623 | 0.738900504 | 0.587851302 | 0.556632106 | 0.624046413 | Proteobacteria | Gammaproteobacteria | Enterobacteriales | Enterobacteriaceae | unclassified |
| Otu00166 | 5.249393324 | -1.694813644 | 2.889281786 | -0.586586484 | 0.557481462 | 0.624046413 | Armatimonadetes | unclassified | unclassified | unclassified | unclassified |
| Otu00213 | 2.1898227 | -1.206733451 | 2.175663777 | -0.554650707 | 0.579133599 | 0.643481776 | Proteobacteria | Alphaproteobacteria | Sphingomonadales | unclassified | unclassified |
| Otu00118 | 27.46707442 | -1.275391834 | 2.972312219 | -0.429090802 | 0.667857146 | 0.736607146 | Bacteroidetes | Bacteroidia | Bacteroidales | Porphyromonadaceae | Dysgonomonas |
| Otu00163 | 3.284861225 | -0.804157901 | 2.00391846 | -0.401292726 | 0.688204619 | 0.753508706 | Proteobacteria | Betaproteobacteria | Burkholderiales | Comamonadaceae | Verminephrobacter |
| Otu00102 | 5.728157963 | 0.630730238 | 1.663161447 | 0.379235726 | 0.704512824 | 0.765774809 | Proteobacteria | Gammaproteobacteria | Xanthomonadales | Xanthomonadaceae | Stenotrophomonas |
| Otu00029 | 54.75387093 | 0.594227278 | 1.736140871 | 0.342269045 | 0.732148429 | 0.790088233 | Proteobacteria | Alphaproteobacteria | Sphingomonadales | unclassified | unclassified |
| Otu00027 | 20.17380473 | 0.414066553 | 1.297966165 | 0.319011824 | 0.749717547 | 0.8032688 | Firmicutes | Bacilli | Lactobacillales | Streptococcaceae | Streptococcus |
| Otu00055 | 6.520711474 | -0.425519525 | 1.456953156 | -0.292061226 | 0.770239812 | 0.819404055 | Proteobacteria | Gammaproteobacteria | Pseudomonadales | Moraxellaceae | Psychrobacter |
| Otu00173 | 2.05679223 | -0.584554042 | 2.086354739 | -0.280179603 | 0.779339715 | 0.823246178 | Actinobacteria | Actinobacteria | Kineosporiales | Kineosporiaceae | unclassified |
| Otu00026 | 109.59509 | -0.297585419 | 1.156092239 | -0.257406294 | 0.796865145 | 0.835872529 | Firmicutes | Bacilli | Bacillales | Staphylococcaceae | Staphylococcus |
| Otu00030 | 5.679230633 | -0.289422723 | 1.539573029 | -0.187988954 | 0.85088531 | 0.886338865 | Proteobacteria | Gammaproteobacteria | Pasteurellales | Pasteurellaceae | Pasteurella |
| Otu00014 | 222.2223755 | -0.241870601 | 1.509141381 | -0.160270339 | 0.872668123 | 0.902760127 | Firmicutes | Clostridia | Clostridiales | Peptostreptococcaceae | Peptoclostridium |
| Otu00025 | 67.02470005 | 0.184404593 | 1.409437279 | 0.130835615 | 0.895905349 | 0.915106054 | Proteobacteria | Gammaproteobacteria | Enterobacteriales | Enterobacteriaceae | unclassified |
| Otu00378 | 2.184572527 | -0.381081229 | 2.938178786 | -0.129699809 | 0.896803933 | 0.915106054 | Proteobacteria | Betaproteobacteria | Nitrosomonadales | Nitrosomonadaceae | Nitrosospira |
| Otu00053 | 3.446043179 | 0.161556172 | 2.053540016 | 0.078672035 | 0.937293489 | 0.949959618 | Actinobacteria | Actinobacteria | Micrococcales | Intrasporangiaceae | unclassified |
| Otu00013 | 58.65671401 | 0.049641694 | 2.171118702 | 0.022864569 | 0.981758303 | 0.982497068 | Proteobacteria | Gammaproteobacteria | Enterobacteriales | Enterobacteriaceae | Proteus |
| Otu00131 | 3.445103639 | 0.039143922 | 1.78426258 | 0.021938431 | 0.982497068 | 0.982497068 | Firmicutes | Bacilli | Bacillales | unclassified | unclassified |
| Otu00980 | 0.310519044 | -2.246384137 | 2.963999942 | -0.7578894 | 0.448517197 | NA | Cyanobacteria | ML635J-21 | unclassified | unclassified | unclassified |
| Otu01196 | 0.042690782 | -1.034877247 | 2.970871604 | -0.34834129 | 0.727583888 | NA | Cyanobacteria | Melainabacteria | Gastranaerophilales | unclassified | unclassified |
| Otu00525 | 0.153358398 | -2.315458844 | 2.960922498 | -0.782005894 | 0.43421111 | NA | Actinobacteria | Thermoleophilia | Gaiellales | unclassified | unclassified |
| Otu00627 | 0.124060073 | -1.766185385 | 2.963046739 | -0.596070714 | 0.551127988 | NA | Actinobacteria | Thermoleophilia | Solirubrobacterales | Solirubrobacteraceae | Solirubrobacter |
| Otu00864 | 0.455713904 | -2.346223432 | 2.962787835 | -0.791897214 | 0.428420612 | NA | Actinobacteria | Thermoleophilia | Solirubrobacterales | Solirubrobacteraceae | Solirubrobacter |
| Otu00780 | 0.075558789 | -1.858830864 | 2.965469349 | -0.626825182 | 0.530773835 | NA | Actinobacteria | Thermoleophilia | Solirubrobacterales | Solirubrobacteraceae | Solirubrobacter |
| Otu00284 | 0.588768823 | -2.257273546 | 2.100993317 | -1.074383972 | 0.282650623 | NA | Actinobacteria | Thermoleophilia | Solirubrobacterales | Solirubrobacteraceae | Solirubrobacter |
| Otu00673 | 0.095568896 | -2.002496851 | 2.963932595 | -0.67562159 | 0.499280931 | NA | Actinobacteria | Thermoleophilia | Solirubrobacterales | unclassified | unclassified |
| Otu00257 | 0.627003978 | -3.31664017 | 2.94930304 | -1.124550487 | 0.260779565 | NA | Actinobacteria | Thermoleophilia | Solirubrobacterales | 0319-6M6 | unclassified |
| Otu00335 | 0.994265292 | -1.344454495 | 2.798015027 | -0.480502957 | 0.630869801 | NA | Actinobacteria | Thermoleophilia | Solirubrobacterales | 288-2 | unclassified |
| Otu00230 | 0.628022715 | -1.53140697 | 2.341360078 | -0.65406726 | 0.513068474 | NA | Actinobacteria | Thermoleophilia | Solirubrobacterales | 288-2 | unclassified |
| Otu00253 | 0.730691921 | -2.782941426 | 2.94297988 | -0.945620269 | 0.344342302 | NA | Actinobacteria | Thermoleophilia | Solirubrobacterales | 288-2 | unclassified |
| Otu00879 | 0.064621265 | -1.7052699 | 2.967814841 | -0.574587699 | 0.565570173 | NA | Actinobacteria | Thermoleophilia | Solirubrobacterales | 288-2 | unclassified |
| Otu00522 | 0.719408596 | -3.261042895 | 2.954207248 | -1.103863955 | 0.269652157 | NA | Actinobacteria | Thermoleophilia | Solirubrobacterales | S1-80 | unclassified |
| Otu01129 | 0.037779395 | -1.507802737 | 2.96938731 | -0.507782441 | 0.611605925 | NA | Actinobacteria | Thermoleophilia | Solirubrobacterales | Elev-16S-1332 | unclassified |
| Otu00812 | 0.101671278 | -0.216184927 | 2.971152733 | -0.072761297 | 0.94199607 | NA | Actinobacteria | Thermoleophilia | Solirubrobacterales | 480-2 | unclassified |
| Otu00542 | 0.384289054 | -1.827968541 | 2.958420493 | -0.617886654 | 0.536650057 | NA | Actinobacteria | Thermoleophilia | Solirubrobacterales | 480-2 | unclassified |
| Otu00254 | 1.413375643 | -3.73525024 | 2.527266363 | -1.477980436 | 0.139413015 | NA | Actinobacteria | Thermoleophilia | Solirubrobacterales | 480-2 | unclassified |
| Otu00180 | 1.465728496 | -3.681310086 | 1.878761363 | -1.959434635 | 0.050061908 | NA | Actinobacteria | Thermoleophilia | Solirubrobacterales | 480-2 | unclassified |
| Otu00736 | 0.443300453 | -2.442736349 | 2.95963757 | -0.825349824 | 0.409173012 | NA | Actinobacteria | Thermoleophilia | Solirubrobacterales | 480-2 | unclassified |
| Otu00456 | 0.357434969 | -2.952272237 | 2.956214501 | -0.998666449 | 0.317956299 | NA | Actinobacteria | Thermoleophilia | Solirubrobacterales | 480-2 | unclassified |
| Otu00512 | 0.361578713 | -2.722646622 | 2.957722704 | -0.920521257 | 0.35730043 | NA | Actinobacteria | Thermoleophilia | Solirubrobacterales | 480-2 | unclassified |
| Otu01023 | 0.130073613 | -0.697291423 | 2.971407674 | -0.234667033 | 0.814467177 | NA | Actinobacteria | Thermoleophilia | Solirubrobacterales | 480-2 | unclassified |
| Otu00363 | 1.722349105 | -4.252527604 | 2.945629303 | -1.443673717 | 0.148830778 | NA | Actinobacteria | Thermoleophilia | Solirubrobacterales | 480-2 | unclassified |
| Otu00791 | 0.16081182 | -1.277604016 | 2.971001265 | -0.43002473 | 0.667177652 | NA | Actinobacteria | Thermoleophilia | Solirubrobacterales | 480-2 | unclassified |
| Otu00122 | 1.168713304 | -4.026054423 | 2.943820247 | -1.367629164 | 0.171428183 | NA | Actinobacteria | Thermoleophilia | Solirubrobacterales | Elev-16S-1332 | unclassified |
| Otu01009 | 0.052449191 | -1.232545126 | 2.968503607 | -0.415207556 | 0.677989968 | NA | Actinobacteria | Thermoleophilia | Solirubrobacterales | Elev-16S-1332 | unclassified |
| Otu00240 | 1.338761775 | -2.430743607 | 1.707610734 | -1.423476416 | 0.154598094 | NA | Actinobacteria | Thermoleophilia | Solirubrobacterales | 480-2 | unclassified |
| Otu00485 | 0.186544422 | -2.090744032 | 2.958394314 | -0.706715809 | 0.479743103 | NA | Actinobacteria | Thermoleophilia | Solirubrobacterales | unclassified | unclassified |
| Otu00993 | 0.283825688 | -1.761212556 | 2.969552595 | -0.593090205 | 0.553120786 | NA | Actinobacteria | Thermoleophilia | Solirubrobacterales | 480-2 | unclassified |
| Otu00797 | 0.181776227 | -1.86079223 | 2.965767489 | -0.627423504 | 0.530381665 | NA | Actinobacteria | Thermoleophilia | Solirubrobacterales | 0319-6M6 | unclassified |
| Otu00798 | 0.130858905 | -2.067355442 | 2.964575212 | -0.697353008 | 0.485581902 | NA | Actinobacteria | Thermoleophilia | Solirubrobacterales | 0319-6M6 | unclassified |
| Otu01001 | 0.069100476 | -1.134102437 | 2.970996582 | -0.381724585 | 0.702665662 | NA | Actinobacteria | Thermoleophilia | Solirubrobacterales | unclassified | unclassified |
| Otu00395 | 0.906434667 | -0.388897103 | 2.937579671 | -0.132386913 | 0.894678266 | NA | Actinobacteria | Thermoleophilia | Solirubrobacterales | Patulibacteraceae | Patulibacter |
| Otu00557 | 0.400337831 | -1.852857127 | 2.964638168 | -0.624985925 | 0.531980296 | NA | Actinobacteria | Thermoleophilia | Solirubrobacterales | unclassified | unclassified |
| Otu00737 | 0.250181608 | -1.909011049 | 2.967869627 | -0.643226047 | 0.520077434 | NA | Actinobacteria | Thermoleophilia | Solirubrobacterales | unclassified | unclassified |
| Otu01166 | 0.159248512 | -1.660395234 | 2.968967855 | -0.559249987 | 0.575991123 | NA | Actinobacteria | Thermoleophilia | Solirubrobacterales | unclassified | unclassified |
| Otu00337 | 1.130255182 | -3.110510385 | 2.841750807 | -1.09457535 | 0.27370272 | NA | Actinobacteria | Thermoleophilia | Solirubrobacterales | unclassified | unclassified |
| Otu00656 | 0.24915521 | -1.42582129 | 2.966746294 | -0.480601018 | 0.630800091 | NA | Actinobacteria | Thermoleophilia | Solirubrobacterales | unclassified | unclassified |
| Otu00665 | 0.532129401 | -1.742644981 | 2.962751695 | -0.58818462 | 0.556408379 | NA | Actinobacteria | Thermoleophilia | Solirubrobacterales | Patulibacteraceae | Patulibacter |
| Otu00279 | 0.788465739 | -0.69704829 | 2.935198535 | -0.237479094 | 0.812285136 | NA | Actinobacteria | Thermoleophilia | Solirubrobacterales | unclassified | unclassified |
| Otu00551 | 0.187407907 | -2.395932782 | 2.961394193 | -0.809055677 | 0.418483122 | NA | Actinobacteria | Thermoleophilia | Solirubrobacterales | Patulibacteraceae | Patulibacter |
| Otu00746 | 0.474230132 | -2.543968228 | 2.960635137 | -0.85926435 | 0.390194688 | NA | Actinobacteria | Thermoleophilia | Gaiellales | unclassified | unclassified |
| Otu01378 | 0.037779395 | -1.507802737 | 2.96938731 | -0.507782441 | 0.611605925 | NA | Actinobacteria | Thermoleophilia | Gaiellales | unclassified | unclassified |
| Otu00900 | 0.022472707 | -0.489654915 | 2.973778911 | -0.164657471 | 0.869213593 | NA | Actinobacteria | Thermoleophilia | Gaiellales | unclassified | unclassified |
| Otu00559 | 0.174961347 | -1.605394938 | 2.959286943 | -0.54249384 | 0.58747835 | NA | Actinobacteria | Thermoleophilia | Gaiellales | unclassified | unclassified |
| Otu00919 | 0.134103481 | -0.790058097 | 2.970391695 | -0.265977749 | 0.790256346 | NA | Actinobacteria | Thermoleophilia | Gaiellales | unclassified | unclassified |
| Otu00375 | 0.448391117 | 0.297250822 | 2.943848203 | 0.100973556 | 0.919571452 | NA | Actinobacteria | Thermoleophilia | Gaiellales | Gaiellaceae | Gaiella |
| Otu00322 | 0.574967431 | -3.448601179 | 2.951978331 | -1.168233907 | 0.242712427 | NA | Actinobacteria | Thermoleophilia | Gaiellales | Gaiellaceae | Gaiella |
| Otu00219 | 0.439593923 | -1.485422015 | 2.967600175 | -0.500546545 | 0.616690291 | NA | Actinobacteria | Thermoleophilia | Gaiellales | Gaiellaceae | Gaiella |
| Otu01032 | 0.055517327 | -0.578806138 | 2.974205817 | -0.194608636 | 0.845699341 | NA | Actinobacteria | Thermoleophilia | Gaiellales | unclassified | unclassified |
| Otu00588 | 0.124146618 | -0.743215684 | 2.96148549 | -0.250960434 | 0.801844699 | NA | Actinobacteria | Rubrobacteria | Rubrobacterales | Rubrobacteriaceae | Rubrobacter |
| Otu00626 | 0.493493299 | -2.706763392 | 2.959083993 | -0.914730166 | 0.360333304 | NA | Actinobacteria | Rubrobacteria | Rubrobacterales | Rubrobacteriaceae | Rubrobacter |
| Otu00582 | 0.143680217 | -0.529898737 | 2.958447058 | -0.179113814 | 0.857848335 | NA | Actinobacteria | Rubrobacteria | Rubrobacterales | Rubrobacteriaceae | Rubrobacter |
| Otu00265 | 0.575060325 | -2.027029105 | 2.952646464 | -0.686512635 | 0.492389899 | NA | Actinobacteria | Actinobacteria | Micromonosporales | Micromonosporaceae | Actinoplanes |
| Otu00232 | 0.949424147 | -3.604515307 | 2.942269771 | -1.225079815 | 0.22054515 | NA | Actinobacteria | Actinobacteria | Micromonosporales | Micromonosporaceae | Virgisporangium |
| Otu00502 | 0.205347739 | -1.996009738 | 2.959260182 | -0.674496197 | 0.499995903 | NA | Actinobacteria | Actinobacteria | Micromonosporales | Micromonosporaceae | unclassified |
| Otu00266 | 0.568089375 | -2.644034798 | 2.94693237 | -0.897215974 | 0.369603683 | NA | Actinobacteria | Actinobacteria | Micromonosporales | Micromonosporaceae | unclassified |
| Otu00248 | 0.916218503 | -1.050891316 | 2.197025929 | -0.478324494 | 0.632419265 | NA | Actinobacteria | Actinobacteria | Micromonosporales | Micromonosporaceae | unclassified |
| Otu00346 | 0.842903713 | 1.444187202 | 2.941648501 | 0.490944857 | 0.62346545 | NA | Actinobacteria | Actinobacteria | Micromonosporales | Micromonosporaceae | unclassified |
| Otu00309 | 0.545366902 | -1.061848174 | 2.941796748 | -0.360952256 | 0.718135138 | NA | Actinobacteria | Actinobacteria | Micromonosporales | Micromonosporaceae | unclassified |
| Otu00466 | 0.480444781 | 0.90600405 | 2.945611767 | 0.30757755 | 0.758403805 | NA | Actinobacteria | Actinobacteria | Micromonosporales | Micromonosporaceae | unclassified |
| Otu00968 | 0.165105478 | -1.122650132 | 2.970462242 | -0.377937856 | 0.705476759 | NA | Actinobacteria | Actinobacteria | Micromonosporales | Micromonosporaceae | Dactylosporangium |
| Otu00316 | 0.960193859 | -4.131277291 | 2.946383703 | -1.402151827 | 0.160869913 | NA | Actinobacteria | Actinobacteria | Pseudonocardiales | Pseudonocardiaceae | unclassified |
| Otu00586 | 1.070115383 | -2.864638107 | 2.955911594 | -0.969121713 | 0.332484465 | NA | Actinobacteria | Actinobacteria | Pseudonocardiales | Pseudonocardiaceae | Actinophytocola |
| Otu00671 | 0.684691266 | -3.165125955 | 2.955344216 | -1.07098386 | 0.284176687 | NA | Actinobacteria | Actinobacteria | Pseudonocardiales | Pseudonocardiaceae | Pseudonocardia |
| Otu01207 | 0.037779395 | -1.507802737 | 2.96938731 | -0.507782441 | 0.611605925 | NA | Actinobacteria | Actinobacteria | Pseudonocardiales | Pseudonocardiaceae | Lentzea |
| Otu00695 | 0.197094734 | -1.321962107 | 2.964991727 | -0.445856929 | 0.655700596 | NA | Actinobacteria | Actinobacteria | unclassified | unclassified | unclassified |
| Otu00577 | 0.252417614 | -2.488783583 | 2.960271914 | -0.840728033 | 0.400500312 | NA | Actinobacteria | Actinobacteria | Streptomycetales | Streptomycetaceae | Streptomyces |
| Otu00377 | 0.320287168 | -1.258242301 | 2.956183051 | -0.425630713 | 0.670376957 | NA | Actinobacteria | Actinobacteria | Streptomycetales | Streptomycetaceae | Streptomyces |
| Otu00494 | 0.307314268 | -1.375544676 | 2.957526889 | -0.465099635 | 0.641860108 | NA | Actinobacteria | Actinobacteria | Streptomycetales | Streptomycetaceae | unclassified |
| Otu00440 | 0.748773821 | -2.888754333 | 2.953781945 | -0.977984965 | 0.32808176 | NA | Actinobacteria | Actinobacteria | Streptomycetales | Streptomycetaceae | unclassified |
| Otu00581 | 0.211836745 | -1.75706359 | 2.966802508 | -0.592241508 | 0.553688878 | NA | Actinobacteria | Actinobacteria | Frankiales | Cryptosporangiaceae | Cryptosporangium |
| Otu00609 | 0.135742389 | -0.920868608 | 2.959081259 | -0.311200852 | 0.755647936 | NA | Actinobacteria | Actinobacteria | unclassified | unclassified | unclassified |
| Otu00089 | 1.484055471 | -2.940017579 | 1.891478377 | -1.554349029 | 0.120101184 | NA | Actinobacteria | Actinobacteria | unclassified | unclassified | unclassified |
| Otu00530 | 0.499539852 | -1.132987598 | 2.962166368 | -0.382486146 | 0.702100803 | NA | Actinobacteria | Actinobacteria | Micrococcales | Brevibacteriaceae | Brevibacterium |
| Otu00953 | 0.063834298 | -0.190064354 | 2.971719883 | -0.063957695 | 0.949003912 | NA | Actinobacteria | Actinobacteria | Kineosporiales | Kineosporiaceae | unclassified |
| Otu00483 | 0.457266542 | -2.309532928 | 2.964156698 | -0.779153453 | 0.435889326 | NA | Actinobacteria | Actinobacteria | Actinomycetales | Actinomycetaceae | Actinomyces |
| Otu00333 | 0.321019617 | -2.174924709 | 2.958687823 | -0.735097732 | 0.462279975 | NA | Actinobacteria | Actinobacteria | Micrococcales | Dermabacteraceae | unclassified |
| Otu00065 | 0.00787546 | -1.097607542 | 2.973987649 | -0.369069301 | 0.712076071 | NA | Actinobacteria | Actinobacteria | Actinomycetales | Actinomycetaceae | Actinomyces |
| Otu00450 | 0.278499979 | -2.578437973 | 2.960348598 | -0.870991334 | 0.383758885 | NA | Actinobacteria | Actinobacteria | Actinomycetales | Actinomycetaceae | unclassified |
| Otu00307 | 1.653809182 | -3.861154814 | 2.393650543 | -1.613082087 | 0.106726675 | NA | Actinobacteria | Actinobacteria | Actinomycetales | Actinomycetaceae | Actinomyces |
| Otu00067 | 0.500097622 | -1.768471656 | 2.960719444 | -0.597311461 | 0.550299454 | NA | Actinobacteria | Actinobacteria | Micrococcales | unclassified | unclassified |

| | | | | | | | | | | | |
|---|---|---|---|---|---|---|---|---|---|---|---|
| Otu00217 | 1.232096378 | -1.859575163 | 2.931118626 | -0.634425078 | 0.525803448 | NA | Actinobacteria | Actinobacteria | Micrococcales | Micrococcaceae | Arthrobacter |
| Otu00303 | 0.868891138 | -3.520878631 | 2.94800964 | -1.194323988 | 0.232351258 | NA | Actinobacteria | Actinobacteria | Micrococcales | Promicromonosporaceae | Cellulosimicrobium |
| Otu01003 | 0.180054734 | -1.389195945 | 2.970436243 | -0.467674049 | 0.640017702 | NA | Actinobacteria | Actinobacteria | Micrococcales | Beutenbergiaceae | Salana |
| Otu00611 | 0.417596788 | -2.3459903 | 2.963872376 | -0.791528785 | 0.428635486 | NA | Actinobacteria | Actinobacteria | Micrococcales | unclassified | unclassified |
| Otu01049 | 0.080248837 | -1.885018775 | 2.965175657 | -0.635719091 | 0.524959531 | NA | Actinobacteria | Actinobacteria | Micrococcales | Microbacteriaceae | unclassified |
| Otu00267 | 0.727431328 | -1.876144085 | 2.940850588 | -0.637959675 | 0.52349993 | NA | Actinobacteria | Actinobacteria | Micrococcales | Intrasporangiaceae | unclassified |
| Otu00401 | 0.678715921 | -3.076610156 | 2.956242378 | -1.040716478 | 0.298007153 | NA | Actinobacteria | Actinobacteria | Micrococcales | Intrasporangiaceae | unclassified |
| Otu00431 | 0.104291221 | -0.966989908 | 2.968408251 | -0.325760416 | 0.74460563 | NA | Actinobacteria | Actinobacteria | Kineosporiales | Kineosporiaceae | Kineosporia |
| Otu00738 | 0.794316944 | -2.852089455 | 2.958820859 | -0.963927723 | 0.335082163 | NA | Actinobacteria | Actinobacteria | Kineosporiales | Kineosporiaceae | unclassified |
| Otu01079 | 0.191068772 | -1.053968591 | 2.971679105 | -0.354671064 | 0.722836031 | NA | Actinobacteria | Actinobacteria | unclassified | unclassified | unclassified |
| Otu00268 | 1.039906779 | -3.4021917 | 1.641201682 | -2.072988188 | 0.038173379 | NA | Actinobacteria | Actinobacteria | Frankiales | Geodermatophilaceae | Modestobacter |
| Otu00301 | 0.640563606 | -3.312244917 | 2.947679342 | -1.123678845 | 0.261149294 | NA | Actinobacteria | Actinobacteria | Frankiales | Geodermatophilaceae | Modestobacter |
| Otu00454 | 0.221479825 | -1.030623652 | 2.95284236 | -0.349027657 | 0.727068546 | NA | Actinobacteria | Actinobacteria | Frankiales | Geodermatophilaceae | unclassified |
| Otu00170 | 0.369922494 | 0.418299234 | 2.964402253 | 0.141107447 | 0.887785061 | NA | Actinobacteria | Actinobacteria | Corynebacteriales | Nocardiaceae | Gordonia |
| Otu00153 | 1.846319181 | -3.398740375 | 1.661644554 | -2.045407585 | 0.040814692 | NA | Actinobacteria | Actinobacteria | Corynebacteriales | Nocardiaceae | Williamsia |
| Otu00198 | 0.895808011 | -0.318835643 | 2.912969372 | -0.109453826 | 0.912842543 | NA | Actinobacteria | Actinobacteria | Corynebacteriales | Nocardiaceae | unclassified |
| Otu00690 | 0.066135398 | -1.770597856 | 2.966758237 | -0.596812316 | 0.550632694 | NA | Actinobacteria | Actinobacteria | Corynebacteriales | Nocardiaceae | Nocardia |
| Otu00184 | 1.666913948 | -3.5268045 | 2.482889576 | -1.420443557 | 0.15547859 | NA | Actinobacteria | Actinobacteria | Corynebacteriales | Nocardiaceae | Rhodococcus |
| Otu00591 | 0.151493455 | -2.297053046 | 2.961980086 | -0.775512657 | 0.438036789 | NA | Actinobacteria | Actinobacteria | Corynebacteriales | Nocardiaceae | Rhodococcus |
| Otu00227 | 0.768355861 | -2.110465905 | 2.957136424 | -0.713685675 | 0.475421566 | NA | Actinobacteria | Actinobacteria | Corynebacteriales | Mycobacteriaceae | Mycobacterium |
| Otu00366 | 1.316925492 | -3.576110956 | 2.457310099 | -1.455294941 | 0.145587627 | NA | Actinobacteria | Actinobacteria | Corynebacteriales | Mycobacteriaceae | Mycobacterium |
| Otu00923 | 0.068670366 | -1.248992914 | 2.968878601 | -0.420695179 | 0.673977682 | NA | Actinobacteria | Actinobacteria | Corynebacteriales | Mycobacteriaceae | Mycobacterium |
| Otu00422 | 0.686154693 | -0.689928705 | 2.953174738 | -0.233622717 | 0.81527789 | NA | Actinobacteria | Actinobacteria | Corynebacteriales | Corynebacteriaceae | Corynebacterium_1 |
| Otu00017 | 0.019422142 | -1.259169883 | 2.972536871 | -0.423601098 | 0.671856754 | NA | Actinobacteria | Actinobacteria | Corynebacteriales | Corynebacteriaceae | Corynebacterium |
| Otu00705 | 0.095568896 | -2.002496851 | 2.963932595 | -0.67562159 | 0.499280931 | NA | Actinobacteria | Actinobacteria | Corynebacteriales | Corynebacteriaceae | Corynebacterium |
| Otu00704 | 0.176124189 | -0.017674742 | 2.960825863 | -0.005969531 | 0.995237032 | NA | Actinobacteria | Actinobacteria | Frankiales | Geodermatophilaceae | unclassified |
| Otu00195 | 0.894250777 | -2.910483484 | 2.951955882 | -0.985950875 | 0.32415722 | NA | Actinobacteria | Actinobacteria | Frankiales | Acidothermaceae | Acidothermus |
| Otu00423 | 0.547046117 | 0.925000787 | 2.95003653 | 0.313555706 | 0.753858505 | NA | Actinobacteria | Actinobacteria | unclassified | unclassified | unclassified |
| Otu00221 | 0.524187383 | -2.554149334 | 2.961841405 | -0.862351822 | 0.388493943 | NA | Actinobacteria | Actinobacteria | Frankiales | Acidothermaceae | Acidothermus |
| Otu00397 | 0.575887473 | 0.336116832 | 2.943977156 | 0.114171005 | 0.909102236 | NA | Actinobacteria | Actinobacteria | Frankiales | Acidothermaceae | Acidothermus |
| Otu00753 | 0.029331346 | -0.998163731 | 2.972727407 | -0.335773717 | 0.737041511 | NA | Actinobacteria | Actinobacteria | Streptosporangiales | Thermomonosporaceae | Actinomadura |
| Otu00809 | 0.070919576 | -1.396949539 | 2.966531951 | -0.47090325 | 0.637709827 | NA | Actinobacteria | Actinobacteria | Streptosporangiales | Thermomonosporaceae | Actinocorallia |
| Otu01372 | 0.031902721 | -1.11978721 | 2.972039392 | -0.376774014 | 0.706341554 | NA | Actinobacteria | Actinobacteria | Streptosporangiales | Thermomonosporaceae | Actinoallomurus |
| Otu00514 | 0.184791212 | -2.472361298 | 2.960269418 | -0.835181178 | 0.403615706 | NA | Actinobacteria | Actinobacteria | Streptosporangiales | Streptosporangiaceae | Streptosporangium |
| Otu01025 | 0.057042562 | -1.698405656 | 2.967356196 | -0.572363257 | 0.567075903 | NA | Actinobacteria | Actinobacteria | Streptosporangiales | Streptosporangiaceae | Microbispora |
| Otu01303 | 0.022791153 | -1.118881258 | 2.972194713 | -0.376449515 | 0.706582741 | NA | Actinobacteria | Actinobacteria | Propionibacteriales | Nocardioidaceae | Marmoricola |
| Otu00447 | 1.299279072 | -3.418609356 | 2.370494335 | -1.442150401 | 0.149259949 | NA | Actinobacteria | Actinobacteria | Propionibacteriales | Nocardioidaceae | Nocardioides |
| Otu00735 | 0.215385159 | -2.233871437 | 2.962838838 | -0.753963195 | 0.450871322 | NA | Actinobacteria | Actinobacteria | Propionibacteriales | Nocardioidaceae | Nocardioides |
| Otu00616 | 0.232521201 | -0.004031667 | 2.953687303 | -0.001364961 | 0.998910919 | NA | Actinobacteria | Actinobacteria | Propionibacteriales | Nocardioidaceae | Nocardioides |
| Otu00443 | 1.320311449 | -2.449046857 | 2.937186812 | -0.833806977 | 0.404389763 | NA | Actinobacteria | Actinobacteria | Propionibacteriales | Nocardioidaceae | Nocardioides |
| Otu00263 | 1.181834053 | -3.602509335 | 2.236646232 | -1.610674626 | 0.107250659 | NA | Actinobacteria | Actinobacteria | Propionibacteriales | Nocardioidaceae | Nocardioides |
| Otu00270 | 0.736572218 | -3.024498218 | 2.956704803 | -1.022928706 | 0.306341556 | NA | Actinobacteria | Actinobacteria | Propionibacteriales | Nocardioidaceae | Nocardioides |
| Otu00425 | 0.500944159 | -1.681301742 | 2.374734917 | -0.707995545 | 0.478948024 | NA | Actinobacteria | Actinobacteria | Propionibacteriales | Nocardioidaceae | Nocardioides |
| Otu00607 | 0.327050865 | -1.960214293 | 2.960117802 | -0.662208204 | 0.507837797 | NA | Actinobacteria | Actinobacteria | Propionibacteriales | Nocardioidaceae | unclassified |
| Otu01384 | 0.037779395 | -1.507802737 | 2.96938731 | -0.507782441 | 0.611605925 | NA | Actinobacteria | Actinobacteria | Propionibacteriales | Nocardioidaceae | Kribbella |
| Otu00249 | 0.868378284 | -2.449199865 | 2.890414806 | -0.84735238 | 0.396798739 | NA | Actinobacteria | Actinobacteria | Propionibacteriales | Nocardioidaceae | Marmoricola |
| Otu00702 | 0.147961536 | -1.182893744 | 2.965866581 | -0.398835791 | 0.690014203 | NA | Actinobacteria | Actinobacteria | Frankiales | Geodermatophilaceae | unclassified |
| Otu00306 | 0.738435386 | -0.224028747 | 2.936090072 | -0.076301728 | 0.939179051 | NA | Actinobacteria | Actinobacteria | Frankiales | Frankiaceae | Jatrophihabitans |
| Otu00849 | 0.116910228 | -2.058832154 | 2.964448621 | -0.694507619 | 0.487363924 | NA | Actinobacteria | Actinobacteria | Frankiales | Frankiaceae | Jatrophihabitans |
| Otu00606 | 0.133017195 | -0.664780578 | 2.964978123 | -0.224210956 | 0.822593159 | NA | Actinobacteria | Actinobacteria | Frankiales | unclassified | unclassified |
| Otu00360 | 0.383933634 | -2.016919038 | 2.964133304 | -0.680441408 | 0.49622501 | NA | Actinobacteria | Actinobacteria | Frankiales | unclassified | unclassified |
| Otu00536 | 0.200911779 | -2.485075143 | 2.960403936 | -0.839437859 | 0.401223646 | NA | Actinobacteria | Actinobacteria | Frankiales | unclassified | unclassified |
| Otu00245 | 0.302983337 | -2.696484516 | 2.959124431 | -0.911244045 | 0.362166802 | NA | Actinobacteria | Actinobacteria | Frankiales | unclassified | unclassified |
| Otu00271 | 0.360181001 | -2.718171729 | 2.954283276 | -0.920078231 | 0.35753188 | NA | Actinobacteria | Actinobacteria | unclassified | unclassified | unclassified |
| Otu00222 | 0.880325036 | -1.175335568 | 2.933727226 | -0.400628783 | 0.688693451 | NA | Actinobacteria | Actinobacteria | unclassified | unclassified | unclassified |
| Otu00719 | 0.458005444 | -2.773048314 | 2.959066298 | -0.937136257 | 0.348688473 | NA | Actinobacteria | Acidimicrobiia | Acidimicrobiales | Acidimicrobiaceae | unclassified |
| Otu00283 | 1.669490731 | -3.907403166 | 1.863690205 | -2.096594786 | 0.036029461 | NA | Actinobacteria | Acidimicrobiia | Acidimicrobiales | Acidimicrobiaceae | unclassified |
| Otu00774 | 0.197933822 | -2.090056673 | 2.964324335 | -0.705070173 | 0.480766567 | NA | Actinobacteria | Acidimicrobiia | Acidimicrobiales | Acidimicrobiaceae | Illumatobacter |
| Otu00492 | 0.294567608 | -2.733453791 | 2.957612306 | -0.924209635 | 0.35537718 | NA | Actinobacteria | Acidimicrobiia | Acidimicrobiales | Acidimicrobiaceae | Illumatobacter |
| Otu00811 | 0.69758725 | -2.684149737 | 2.960594923 | -0.906625123 | 0.364605071 | NA | Actinobacteria | unclassified | unclassified | unclassified | unclassified |
| Otu00540 | 0.395231743 | -2.990252495 | 2.956297261 | -1.01148573 | 0.311784007 | NA | Actinobacteria | Acidimicrobiia | Acidimicrobiales | Acidimicrobiales_Incertae_Sedis | Candidatus_Microthrix |
| Otu00558 | 0.250192876 | 0.030715011 | 2.962201495 | 0.010368981 | 0.991726898 | NA | Actinobacteria | Acidimicrobiia | Acidimicrobiales | Acidimicrobiales_Incertae_Sedis | Candidatus_Microthrix |
| Otu00344 | 1.447979801 | -4.306413451 | 2.945279993 | -1.462140598 | 0.143702686 | NA | Actinobacteria | Acidimicrobiia | Acidimicrobiales | Iamiaceae | Iamia |
| Otu00598 | 0.142420873 | -2.274699807 | 2.961801163 | -0.768012328 | 0.442479861 | NA | Actinobacteria | Acidimicrobiia | Acidimicrobiales | Iamiaceae | Iamia |
| Otu00639 | 0.246117155 | -1.963807954 | 2.962098746 | -0.662978558 | 0.507344287 | NA | Actinobacteria | Acidimicrobiia | Acidimicrobiales | Iamiaceae | Iamia |
| Otu00273 | 1.138390863 | -3.299043507 | 2.941951377 | -1.121379344 | 0.262126425 | NA | Actinobacteria | Acidimicrobiia | Acidimicrobiales | Iamiaceae | Iamia |
| Otu00419 | 0.406867928 | -2.619093104 | 2.955233163 | -0.886255994 | 0.375479597 | NA | Actinobacteria | Acidimicrobiia | Acidimicrobiales | Iamiaceae | Iamia |
| Otu00192 | 0.119890488 | -1.537683602 | 2.962061605 | -0.519126138 | 0.603672782 | NA | Actinobacteria | Acidimicrobiia | Acidimicrobiales | Iamiaceae | Iamia |
| Otu00981 | 0.437197677 | -2.098552602 | 2.965633716 | -0.707623666 | 0.479178992 | NA | Actinobacteria | Acidimicrobiia | Acidimicrobiales | Iamiaceae | Iamia |
| Otu00503 | 0.759246335 | -3.167885623 | 2.955216366 | -1.071964023 | 0.283736192 | NA | Actinobacteria | Acidimicrobiia | Acidimicrobiales | Iamiaceae | Iamia |
| Otu00476 | 0.34818929 | -1.934217294 | 2.955291373 | -0.654492925 | 0.512794284 | NA | Actinobacteria | Acidimicrobiia | Acidimicrobiales | unclassified | unclassified |
| Otu00455 | 0.566960834 | -3.172482604 | 2.952633028 | -1.074458821 | 0.282617091 | NA | Actinobacteria | Acidimicrobiia | Acidimicrobiales | Acidimicrobiales_Incertae_Sedis | Candidatus_Microthrix |
| Otu00637 | 0.234648326 | -2.374447567 | 2.961392638 | -0.801800996 | 0.422668082 | NA | Actinobacteria | Acidimicrobiia | Acidimicrobiales | Iamiaceae | Iamia |
| Otu00334 | 1.543968181 | -1.979348063 | 2.942968173 | -0.672568627 | 0.501221767 | NA | Actinobacteria | Acidimicrobiia | Acidimicrobiales | unclassified | unclassified |
| Otu00807 | 0.063805443 | -1.276419244 | 2.968202605 | -0.430031037 | 0.667173064 | NA | Actinobacteria | unclassified | unclassified | unclassified | unclassified |
| Otu00914 | 0.163364462 | -1.519222499 | 2.968271551 | -0.511820591 | 0.608776578 | NA | Actinobacteria | unclassified | unclassified | unclassified | unclassified |
| Otu00679 | 0.677742363 | -0.923799188 | 2.965768717 | -0.311487265 | 0.755430222 | NA | Actinobacteria | unclassified | unclassified | unclassified | unclassified |
| Otu00754 | 0.857528433 | -2.730961304 | 2.955556603 | -0.924009136 | 0.355481559 | NA | Actinobacteria | MB-A2-108 | unclassified | unclassified | unclassified |
| Otu00188 | 0.023359873 | -1.323321734 | 2.971664796 | -0.445313259 | 0.656093388 | NA | Firmicutes | Clostridia | Clostridiales | unclassified | unclassified |
| Otu01282 | 0.035582271 | -0.410818162 | 2.973250223 | -0.138171405 | 0.890104953 | NA | Firmicutes | Clostridia | Clostridiales | Family_XI | Gallicola |
| Otu00745 | 0.386115603 | -2.905518957 | 2.957138427 | -0.982544115 | 0.325831861 | NA | Firmicutes | Clostridia | Clostridiales | Family_XIII | [Eubacterium]_brachy_group |
| Otu00297 | 0.260306898 | 0.174733106 | 2.951258146 | 0.05920631 | 0.952787783 | NA | Firmicutes | Clostridia | Clostridiales | Peptostreptococcaceae | unclassified |
| Otu00078 | 0.41793451 | -1.73380141 | 2.969804092 | -0.583810028 | 0.559348131 | NA | Firmicutes | Clostridia | Clostridiales | Peptostreptococcaceae | unclassified |
| Otu00703 | 0.087987767 | -1.551203887 | 2.964489949 | -0.523261645 | 0.600792188 | NA | Firmicutes | Clostridia | Clostridiales | Peptostreptococcaceae | Terrisporobacter |
| Otu01045 | 0.056295622 | -1.692152647 | 2.967418104 | -0.570244094 | 0.568512153 | NA | Firmicutes | Clostridia | Clostridiales | Lachnospiraceae | unclassified |
| Otu01340 | 0.025934339 | -0.578806139 | 2.974205817 | -0.194608637 | 0.845699341 | NA | Firmicutes | Clostridia | Clostridiales | Lachnospiraceae | Lachnoclostridium |
| Otu00605 | 0.111097365 | -2.098869018 | 2.962998266 | -0.708359854 | 0.478721817 | NA | Firmicutes | Clostridia | Clostridiales | Clostridiaceae_1 | Clostridium_sensu_stricto_1 |
| Otu00632 | 0.096856062 | -1.866600506 | 2.96378529 | -0.629802878 | 0.528823562 | NA | Firmicutes | Clostridia | Clostridiales | Clostridiaceae_1 | Clostridium_sensu_stricto_1 |
| Otu01336 | 0.008212655 | -0.894046489 | 2.974210836 | -0.300599567 | 0.763719862 | NA | Firmicutes | Clostridia | Clostridiales | Clostridiaceae_1 | unclassified |
| Otu01174 | 0.012701833 | -1.165106781 | 2.973599677 | -0.391816958 | 0.695193464 | NA | Firmicutes | Clostridia | Clostridiales | Clostridiaceae_1 | unclassified |
| Otu00269 | 0.311577479 | -1.692076159 | 2.949799146 | -0.573624195 | 0.566222136 | NA | Firmicutes | Clostridia | Clostridiales | Clostridiaceae_1 | Clostridium_sensu_stricto_1 |
| Otu00426 | 0.194634506 | -0.910686728 | 2.95448821 | -0.308238403 | 0.757900933 | NA | Firmicutes | Clostridia | Clostridiales | Clostridiaceae_1 | unclassified |
| Otu00870 | 0.078063397 | -1.156531734 | 2.967679797 | -0.38970907 | 0.696751689 | NA | Firmicutes | Clostridia | Clostridiales | Clostridiaceae_1 | Clostridium_sensu_stricto_1 |
| Otu00009 | 0.292456099 | -0.6654764 | 2.961918679 | -0.224677471 | 0.822230192 | NA | Firmicutes | Clostridia | Clostridiales | Ruminococcaceae | Faecalibacterium |
| Otu00006 | 0.098494791 | -1.748086143 | 2.965358048 | -0.589502554 | 0.555524199 | NA | Firmicutes | Clostridia | Clostridiales | Ruminococcaceae | Subdoligranulum |
| Otu00437 | 0.260763089 | -1.593248645 | 2.955224244 | -0.539129526 | 0.589797484 | NA | Proteobacteria | Deltaproteobacteria | Myxococcales | Haliangiaceae | Haliangium |
| Otu00678 | 0.1507892 | 0.237555093 | 2.967344435 | 0.080056461 | 0.936192351 | NA | Proteobacteria | Deltaproteobacteria | Myxococcales | Haliangiaceae | Haliangium |
| Otu00531 | 0.234719763 | -0.080872894 | 2.963753321 | -0.027287323 | 0.978230568 | NA | Proteobacteria | Deltaproteobacteria | Myxococcales | Nannocystaceae | unclassified |
| Otu01396 | 0.039031698 | -1.053436334 | 2.971709996 | -0.354488269 | 0.722972994 | NA | Proteobacteria | Deltaproteobacteria | Myxococcales | Nannocystaceae | Nannocystis |
| Otu01184 | 0.016812502 | -0.883768791 | 2.973554236 | -0.297209575 | 0.766306512 | NA | Proteobacteria | Deltaproteobacteria | Oligoflexales | unclassified | unclassified |
| Otu00513 | 0.082359078 | -0.283425653 | 2.968838887 | -0.095466835 | 0.923944031 | NA | Proteobacteria | Gammaproteobacteria | Xanthomonadales | Xanthomonadaceae | Rhodanobacter |
| Otu00905 | 0.217517105 | -1.057604667 | 2.971692053 | -0.35589309 | 0.721920631 | NA | Proteobacteria | Gammaproteobacteria | Xanthomonadales | Xanthomonadaceae | Luteibacter |
| Otu01289 | 0.033887121 | -0.97440408 | 2.971671678 | -0.327897623 | 0.742989071 | NA | Proteobacteria | Gammaproteobacteria | Xanthomonadales | Xanthomonadaceae | unclassified |
| Otu00452 | 0.506879231 | -0.603053525 | 2.95248444 | -0.204252906 | 0.83815587 | NA | Proteobacteria | Gammaproteobacteria | Xanthomonadales | Xanthomonadaceae | Luteimonas |
| Otu00326 | 1.525236835 | -2.832349988 | 1.715187795 | -1.651335204 | 0.098670148 | NA | Proteobacteria | Gammaproteobacteria | Xanthomonadales | Xanthomonadaceae | unclassified |
| Otu00960 | 0.055078767 | -0.976640647 | 2.971245569 | -0.328697384 | 0.742384432 | NA | Proteobacteria | Gammaproteobacteria | Xanthomonadales | Xanthomonadaceae | Lysobacter |
| Otu00218 | 0.115579003 | -2.12373257 | 2.962768948 | -0.716806679 | 0.47349339 | NA | Proteobacteria | Gammaproteobacteria | Xanthomonadales | Xanthomonadaceae | unclassified |
| Otu00392 | 0.272714235 | -2.431276202 | 2.956833896 | -0.822256605 | 0.410930869 | NA | Proteobacteria | Gammaproteobacteria | Xanthomonadales | Xanthomonadaceae | Pseudoxanthomonas |
| Otu01014 | 0.178352704 | -1.895376473 | 2.966460091 | -0.638935436 | 0.522864934 | NA | Proteobacteria | Gammaproteobacteria | Xanthomonadales | Xanthomonadaceae | Arenimonas |
| Otu00912 | 0.04130738 | -1.37438378 | 2.969085234 | -0.462898055 | 0.643437442 | NA | Proteobacteria | Gammaproteobacteria | Xanthomonadales | Xanthomonadaceae | Mizugakiibacter |
| Otu00125 | 0.414865377 | 0.466791485 | 2.95762898 | 0.157826248 | 0.874593715 | NA | Proteobacteria | Gammaproteobacteria | Pseudomonadales | Pseudomonadaceae | Pseudomonas |
| Otu00885 | 0.063772265 | -1.423496853 | 2.97194453 | -0.478978271 | 0.631954087 | NA | Proteobacteria | Gammaproteobacteria | Pseudomonadales | Pseudomonadaceae | unclassified |
| Otu00646 | 0.114991039 | -2.120511645 | 2.96279839 | -0.715712433 | 0.474168932 | NA | Proteobacteria | Gammaproteobacteria | Pseudomonadales | Pseudomonadaceae | unclassified |
| Otu00546 | 0.381513573 | -2.645982507 | 2.954637545 | -0.895535397 | 0.370500953 | NA | Proteobacteria | Gammaproteobacteria | Cellvibrionales | Cellvibrionaceae | Cellvibrio |
| Otu00913 | 0.086383631 | -0.943112686 | 2.970282582 | -0.317516149 | 0.750851988 | NA | Proteobacteria | Gammaproteobacteria | unclassified | unclassified | unclassified |
| Otu00244 | 1.24912352 | 1.788970176 | 2.938604776 | 0.608782164 | 0.542668837 | NA | Proteobacteria | Gammaproteobacteria | Xanthomonadales | Xanthomonadales_Incertae_Sedis | Acidibacter |
| Otu00830 | 0.206465387 | -0.28448991 | 2.970808501 | -0.09576178 | 0.923709773 | NA | Proteobacteria | Gammaproteobacteria | Xanthomonadales | Xanthomonadales_Incertae_Sedis | Steroidobacter |
| Otu01149 | 0.107590428 | -1.053968576 | 2.971679106 | -0.354671059 | 0.722836035 | NA | Proteobacteria | unclassified | unclassified | unclassified | unclassified |
| Otu01215 | 0.070525734 | -1.599078811 | 2.968376601 | -0.53870483 | 0.590090541 | NA | Proteobacteria | unclassified | unclassified | unclassified | unclassified |

| | | | | | | | | | | | | |
|---|---|---|---|---|---|---|---|---|---|---|---|---|
| Otu00406 | 0.273868408 | -1.380308211 | 2.955608406 | -0.467013224 | 0.640490417 | NA | Proteobacteria | Gammaproteobacteria | Legionellales | Legionellaceae | Legionella |
| Otu00800 | 0.192694288 | -1.289102804 | 2.96969759 | -0.434085548 | 0.66422632 | NA | Proteobacteria | Gammaproteobacteria | Legionellales | Legionellaceae | Legionella |
| Otu00028 | 0.051242402 | -0.682796818 | 2.97445294 | -0.229553747 | 0.818438552 | NA | Proteobacteria | Gammaproteobacteria | Legionellales | Legionellaceae | Legionella |
| Otu00107 | 0.402458032 | -1.868053001 | 2.960464513 | -0.630999964 | 0.528040548 | NA | Proteobacteria | Gammaproteobacteria | Legionellales | Coxiellaceae | Rickettsiella |
| Otu00537 | 0.058264309 | -0.047198403 | 2.968384905 | -0.015900365 | 0.987313879 | NA | Proteobacteria | Gammaproteobacteria | Aeromonadales | Aeromonadaceae | Tolumonas |
| Otu00061 | 0.096232086 | -0.538876958 | 2.969663621 | -0.181460605 | 0.856006052 | NA | Proteobacteria | Gammaproteobacteria | Pasteurellales | Pasteurellaceae | Avibacterium |
| Otu00464 | 0.910289643 | -2.152188799 | 2.948364852 | -0.729960133 | 0.465414554 | NA | Proteobacteria | Gammaproteobacteria | unclassified | unclassified | unclassified |
| Otu00793 | 0.074970825 | -1.854333088 | 2.965520423 | -0.625297696 | 0.531775692 | NA | Proteobacteria | Gammaproteobacteria | Vibrionales | Vibrionaceae | unclassified |
| Otu00564 | 0.329338218 | 1.147583792 | 2.960564139 | 0.387623351 | 0.698294786 | NA | Proteobacteria | Gammaproteobacteria | Pseudomonadales | Moraxellaceae | Moraxella |
| Otu00069 | 1.248067309 | -3.860913463 | 2.550786498 | -1.513616866 | 0.130123041 | NA | Proteobacteria | Gammaproteobacteria | Pseudomonadales | Moraxellaceae | Psychrobacter |
| Otu00207 | 0.14315713 | -1.55926631 | 2.96217214 | -0.526392875 | 0.598615265 | NA | Proteobacteria | Gammaproteobacteria | Pseudomonadales | Moraxellaceae | Psychrobacter |
| Otu00964 | 0.044542275 | -1.011918366 | 2.971290658 | -0.340565257 | 0.733430888 | NA | Proteobacteria | Betaproteobacteria | Burkholderiales | Burkholderiaceae | Burkholderia |
| Otu00467 | 0.242811271 | -0.620895037 | 2.952846639 | -0.210269991 | 0.833456955 | NA | Proteobacteria | Betaproteobacteria | Burkholderiales | Alcaligenaceae | unclassified |
| Otu00325 | 0.81095526 | -2.154759008 | 2.95683357 | -0.728738685 | 0.466161524 | NA | Proteobacteria | Betaproteobacteria | Burkholderiales | Alcaligenaceae | unclassified |
| Otu01176 | 0.008110678 | -1.079036526 | 2.974030903 | -0.36281954 | 0.716739694 | NA | Proteobacteria | Betaproteobacteria | Burkholderiales | Alcaligenaceae | unclassified |
| Otu01410 | 0.022689176 | -1.289899993 | 2.972273832 | -0.433977509 | 0.664304774 | NA | Proteobacteria | Betaproteobacteria | Burkholderiales | Alcaligenaceae | Castellaniella |
| Otu00511 | 1.266322374 | -3.298468485 | 2.953957577 | -1.116626898 | 0.264153886 | NA | Proteobacteria | Betaproteobacteria | Burkholderiales | Burkholderiaceae | Lautropia |
| Otu01089 | 0.073825564 | -0.801916578 | 2.972741911 | -0.269756542 | 0.787347558 | NA | Proteobacteria | Betaproteobacteria | Burkholderiales | unclassified | unclassified |
| Otu00085 | 0.62404549 | -2.410971802 | 2.630342609 | -0.916599911 | 0.359352331 | NA | Proteobacteria | Betaproteobacteria | Burkholderiales | Alcaligenaceae | Sutterella |
| Otu00684 | 0.090700803 | 0.027932867 | 2.96950127 | 0.009406585 | 0.992494742 | NA | Proteobacteria | Betaproteobacteria | Burkholderiales | Oxalobacteraceae | unclassified |
| Otu00287 | 0.679490185 | 0.474892219 | 2.088918427 | 0.227338805 | 0.820160301 | NA | Proteobacteria | Betaproteobacteria | Burkholderiales | Comamonadaceae | unclassified |
| Otu00196 | 0.021659414 | -0.682796831 | 2.97445294 | -0.229553752 | 0.818438548 | NA | Proteobacteria | Betaproteobacteria | Burkholderiales | Comamonadaceae | Aquabacterium |
| Otu01177 | 0.025597144 | -0.746617752 | 2.974134172 | -0.251037011 | 0.801785495 | NA | Proteobacteria | Betaproteobacteria | Burkholderiales | Comamonadaceae | unclassified |
| Otu01055 | 0.25886096 | -1.48542163 | 2.967600179 | -0.500546415 | 0.616690383 | NA | Proteobacteria | Betaproteobacteria | Burkholderiales | Comamonadaceae | unclassified |
| Otu00040 | 0.644950457 | -3.548698372 | 2.95029978 | -1.202826369 | 0.229043518 | NA | Proteobacteria | Betaproteobacteria | unclassified | unclassified | unclassified |
| Otu00236 | 0.929929445 | 0.300128583 | 2.280653591 | 0.131597619 | 0.895302569 | NA | Proteobacteria | Betaproteobacteria | Burkholderiales | Oxalobacteraceae | Massilia |
| Otu00519 | 0.529976881 | -2.748037039 | 2.959439217 | -0.928566812 | 0.353113628 | NA | Proteobacteria | Betaproteobacteria | Burkholderiales | Oxalobacteraceae | Duganella |
| Otu00373 | 0.07748926 | -0.337146119 | 2.965285029 | -0.11369771 | 0.909477428 | NA | Proteobacteria | Betaproteobacteria | Hydrogenophilales | Hydrogenophilaceae | Hydrogenophilus |
| Otu01034 | 0.070551025 | -0.465613792 | 2.972958625 | -0.156616304 | 0.875547252 | NA | Proteobacteria | Betaproteobacteria | SC-I-84 | unclassified | unclassified |
| Otu00907 | 0.05541535 | -0.732881377 | 2.974090503 | -0.246422016 | 0.805355565 | NA | Proteobacteria | Betaproteobacteria | SC-I-84 | unclassified | unclassified |
| Otu00108 | 0.717675932 | -3.562231585 | 2.949621296 | -1.207691167 | 0.227166077 | NA | Proteobacteria | Betaproteobacteria | Neisseriales | Neisseriaceae | Neisseria |
| Otu01332 | 0.016099402 | -0.578806139 | 2.974205817 | -0.194608637 | 0.845699341 | NA | Proteobacteria | Gammaproteobacteria | Pseudomonadales | Moraxellaceae | Acinetobacter |
| Otu00643 | 0.277003426 | -1.054825763 | 2.969839032 | -0.355179439 | 0.722455167 | NA | Proteobacteria | Gammaproteobacteria | Cellvibrionales | Halieaceae | unclassified |
| Otu01258 | 0.037030307 | -0.41081816 | 2.973250223 | -0.138171405 | 0.890104954 | NA | Proteobacteria | Deltaproteobacteria | unclassified | unclassified | unclassified |
| Otu01259 | 0.069758629 | -0.974404078 | 2.971671678 | -0.327897622 | 0.742989071 | NA | Proteobacteria | Deltaproteobacteria | Myxococcales | Polyangiaceae | Sorangium |
| Otu00281 | 0.211315633 | -2.033049837 | 2.961631923 | -0.686462697 | 0.49242138 | NA | Proteobacteria | Deltaproteobacteria | Myxococcales | BIrii41 | unclassified |
| Otu00480 | 0.315316681 | -1.444012176 | 2.955996446 | -0.488502677 | 0.62519383 | NA | Proteobacteria | Deltaproteobacteria | Myxococcales | Polyangiaceae | Byssovorax |
| Otu00667 | 0.115693192 | -0.010820704 | 2.968946993 | -0.003644627 | 0.997092015 | NA | Proteobacteria | Deltaproteobacteria | Myxococcales | Sandaracinaceae | unclassified |
| Otu00766 | 0.059486412 | -1.721476025 | 2.967116306 | -0.580184882 | 0.561789947 | NA | Proteobacteria | Deltaproteobacteria | Myxococcales | Sandaracinaceae | unclassified |
| Otu00565 | 0.148765027 | -1.909104675 | 2.960523105 | -0.644853834 | 0.519021912 | NA | Proteobacteria | Deltaproteobacteria | Myxococcales | Sandaracinaceae | unclassified |
| Otu00596 | 0.123244251 | -1.313461031 | 2.962910155 | -0.443300999 | 0.657548033 | NA | Proteobacteria | Deltaproteobacteria | Myxococcales | Sandaracinaceae | unclassified |
| Otu00389 | 0.121075646 | -0.595358698 | 2.96921422 | -0.200510524 | 0.841081328 | NA | Proteobacteria | Deltaproteobacteria | Myxococcales | unclassified | unclassified |
| Otu00280 | 0.477987226 | -2.146466424 | 2.947068954 | -0.728339397 | 0.466405851 | NA | Proteobacteria | Deltaproteobacteria | Myxococcales | unclassified | unclassified |
| Otu00048 | 0.238766913 | -1.53116293 | 2.971394031 | -0.515301207 | 0.606342556 | NA | Proteobacteria | Deltaproteobacteria | Desulfovibrionales | Desulfovibrionaceae | Desulfovibrio |
| Otu00329 | 0.359376563 | -2.821521503 | 2.953324789 | -0.955371219 | 0.339390003 | NA | Proteobacteria | Deltaproteobacteria | Oligoflexales | Oligoflexaceae | Oligoflexus |
| Otu00773 | 0.070965419 | -1.410168704 | 2.966346963 | -0.475388996 | 0.634509737 | NA | Proteobacteria | Deltaproteobacteria | Oligoflexales | Oligoflexaceae | Oligoflexus |
| Otu01301 | 0.21324218 | -1.180876113 | 2.971985077 | -0.397335815 | 0.69111984 | NA | Proteobacteria | Epsilonproteobacteria | Campylobacterales | Campylobacteraceae | Campylobacter |
| Otu00037 | 0.170460456 | -0.455753707 | 2.970356519 | -0.153434008 | 0.878056028 | NA | Proteobacteria | Epsilonproteobacteria | Campylobacterales | Campylobacteraceae | Campylobacter |
| Otu00138 | 0.62504248 | 1.316348332 | 2.944414754 | 0.447066205 | 0.654827259 | NA | Proteobacteria | Epsilonproteobacteria | Campylobacterales | Campylobacteraceae | Campylobacter |
| Otu00299 | 0.795239149 | -3.971800133 | 2.947753509 | -1.347399001 | 0.177851762 | NA | Proteobacteria | Epsilonproteobacteria | Campylobacterales | Helicobacteraceae | Helicobacter |
| Otu00275 | 0.532999076 | -1.158954792 | 2.442312388 | -0.474531759 | 0.635120756 | NA | Proteobacteria | Alphaproteobacteria | Rhodospirillales | Rhodospirillales_Incertae_Sedis | Reyranella |
| Otu00239 | 0.771388388 | -1.679159593 | 2.310702934 | -0.726687783 | 0.467417239 | NA | Proteobacteria | Alphaproteobacteria | Rhodospirillales | Rhodospirillales_Incertae_Sedis | Reyranella |
| Otu00817 | 0.109031903 | -1.416692107 | 2.96625639 | -0.477602716 | 0.632932998 | NA | Proteobacteria | Alphaproteobacteria | Rhodospirillales | DA111 | unclassified |
| Otu00920 | 0.061835055 | -0.672953437 | 2.969839401 | -0.226595902 | 0.820737979 | NA | Proteobacteria | Alphaproteobacteria | Rhodospirillales | Acetobacteraceae | unclassified |
| Otu00767 | 0.124041089 | 0.109921822 | 2.968100844 | 0.037034396 | 0.970457581 | NA | Proteobacteria | Alphaproteobacteria | Rhodospirillales | Acetobacteraceae | unclassified |
| Otu00231 | 0.461181614 | 1.123110671 | 2.947176957 | 0.381080162 | 0.703143767 | NA | Proteobacteria | Alphaproteobacteria | Rhodospirillales | Acetobacteraceae | Acidiphilium |
| Otu00405 | 0.46554909 | 1.592602644 | 2.950375281 | 0.539796633 | 0.589337289 | NA | Proteobacteria | Alphaproteobacteria | Rhodospirillales | Acetobacteraceae | Acidiphilium |
| Otu00370 | 0.15647641 | -0.540457526 | 2.968311384 | -0.182075752 | 0.855523278 | NA | Proteobacteria | Alphaproteobacteria | Rhodospirillales | Acetobacteraceae | unclassified |
| Otu00769 | 0.176752493 | 0.362658115 | 2.961019372 | 0.122477454 | 0.9025209 | NA | Proteobacteria | Alphaproteobacteria | Rhodospirillales | Acetobacteraceae | unclassified |
| Otu00709 | 0.075698057 | -1.178885537 | 2.967669486 | -0.397242868 | 0.691188374 | NA | Proteobacteria | Alphaproteobacteria | Rhodospirillales | Acetobacteraceae | Roseococcus |
| Otu00698 | 0.294460967 | -0.250581064 | 2.965527014 | -0.084497987 | 0.932660503 | NA | Proteobacteria | Alphaproteobacteria | Rhodospirillales | Acetobacteraceae | Roseomonas |
| Otu00610 | 0.148184067 | 0.59641074 | 2.964342866 | 0.201194925 | 0.84054616 | NA | Proteobacteria | Alphaproteobacteria | Rhodospirillales | Acetobacteraceae | unclassified |
| Otu00642 | 0.879187847 | -2.526771667 | 2.953646862 | -0.855475209 | 0.392288123 | NA | Proteobacteria | Alphaproteobacteria | Rhodospirillales | Acetobacteraceae | Roseomonas |
| Otu00290 | 1.4808442 | -2.263159251 | 2.93453296 | -0.771216164 | 0.440578815 | NA | Proteobacteria | Alphaproteobacteria | Rhodospirillales | Acetobacteraceae | Roseomonas |
| Otu01377 | 0.10080784 | -0.650921913 | 2.971994592 | -0.219018539 | 0.826635606 | NA | Proteobacteria | Alphaproteobacteria | Rhodospirillales | Rhodospirillaceae | Dongia |
| Otu00320 | 0.446980993 | -1.984136232 | 2.948194295 | -0.673000499 | 0.500946973 | NA | Proteobacteria | Alphaproteobacteria | Rhodospirillales | Rhodospirillaceae | Skermanella |
| Otu01181 | 0.031087644 | -0.98541825 | 2.971523153 | -0.331620586 | 0.740175773 | NA | Proteobacteria | Alphaproteobacteria | Rhodospirillales | Rhodospirillaceae | Defluviicoccus |
| Otu00403 | 0.492146492 | 0.22898396 | 2.942700679 | 0.077814221 | 0.937975834 | NA | Proteobacteria | Alphaproteobacteria | Rhodospirillales | Rhodospirillales_Incertae_Sedis | Candidatus_Alysiosphaera |
| Otu00451 | 1.734320034 | -3.476389142 | 2.944469822 | -1.180650287 | 0.237741678 | NA | Proteobacteria | Alphaproteobacteria | Rhodospirillales | Rhodospirillales_Incertae_Sedis | Candidatus_Alysiosphaera |
| Otu01359 | 0.063881956 | -0.55812525 | 2.973484548 | -0.18770074 | 0.85111125 | NA | Proteobacteria | Alphaproteobacteria | Rhodospirillales | Rhodospirillales_Incertae_Sedis | Candidatus_Alysiosphaera |
| Otu00374 | 0.263060728 | -2.549452118 | 2.955973507 | -0.862474617 | 0.388426395 | NA | Proteobacteria | Alphaproteobacteria | Rhodospirillales | Rhodospirillales_Incertae_Sedis | Candidatus_Alysiosphaera |
| Otu00101 | 0.019263167 | -1.256736319 | 2.972570818 | -0.422777587 | 0.672457541 | NA | Proteobacteria | Alphaproteobacteria | Rhizobiales | A0839 | unclassified |
| Otu00194 | 0.092225359 | 0.181430395 | 2.964056314 | 0.061210171 | 0.951191829 | NA | Proteobacteria | Alphaproteobacteria | Caulobacterales | Caulobacteraceae | unclassified |
| Otu00457 | 0.267836538 | 0.806134134 | 2.953203672 | 0.272969366 | 0.784876764 | NA | Proteobacteria | Alphaproteobacteria | Caulobacterales | Caulobacteraceae | unclassified |
| Otu00729 | 0.099720662 | 0.260552574 | 2.967584228 | 0.087799555 | 0.930035992 | NA | Proteobacteria | Alphaproteobacteria | Caulobacterales | Caulobacteraceae | Caulobacter |
| Otu01317 | 0.208967255 | -1.337214644 | 2.972375466 | -0.449880797 | 0.652796395 | NA | Proteobacteria | Alphaproteobacteria | Caulobacterales | Caulobacteraceae | Phenylobacterium |
| Otu00590 | 0.12535857 | -1.233079935 | 2.965476845 | -0.415811689 | 0.677547805 | NA | Proteobacteria | Alphaproteobacteria | unclassified | unclassified | unclassified |
| Otu00414 | 0.24904423 | -1.942830824 | 2.956210484 | -0.657203144 | 0.511050304 | NA | Proteobacteria | Alphaproteobacteria | Rhizobiales | Hyphomicrobiaceae | Rhodomicrobium |
| Otu00752 | 0.22662902 | 0.63002081 | 2.956735645 | 0.213079858 | 0.831264675 | NA | Proteobacteria | Alphaproteobacteria | Rhizobiales | Hyphomicrobiaceae | Rhodomicrobium |
| Otu00759 | 0.583039988 | -2.105442388 | 2.963351632 | -0.710493606 | 0.477398094 | NA | Proteobacteria | Alphaproteobacteria | Rhizobiales | Hyphomicrobiaceae | Pedomicrobium |
| Otu00790 | 0.124982232 | -1.755518787 | 2.966161444 | -0.591848697 | 0.55395191 | NA | Proteobacteria | Alphaproteobacteria | Rhizobiales | Hyphomicrobiaceae | Pedomicrobium |
| Otu00926 | 0.020170373 | -0.781311229 | 2.973685832 | -0.262741686 | 0.792749684 | NA | Proteobacteria | Alphaproteobacteria | Rhizobiales | Hyphomicrobiaceae | Hyphomicrobium |
| Otu00959 | 0.082422226 | -0.934980356 | 2.970543556 | -0.314750596 | 0.752951025 | NA | Proteobacteria | Alphaproteobacteria | Rhizobiales | Hyphomicrobiaceae | Pedomicrobium |
| Otu00672 | 0.317571402 | -1.473252705 | 2.967618452 | -0.496442763 | 0.61958206 | NA | Proteobacteria | Alphaproteobacteria | Rhizobiales | Hyphomicrobiaceae | Hyphomicrobium |
| Otu00747 | 0.326014681 | -1.526331085 | 2.963076651 | -0.515116976 | 0.60647128 | NA | Proteobacteria | Alphaproteobacteria | Rhizobiales | unclassified | unclassified |
| Otu00771 | 0.438742277 | -2.588959856 | 2.96115083 | -0.874308674 | 0.381950177 | NA | Proteobacteria | Alphaproteobacteria | Rhizobiales | Rhodobiaceae | unclassified |
| Otu00755 | 0.08306861 | -1.466735455 | 2.966051621 | -0.494507731 | 0.620947648 | NA | Proteobacteria | Alphaproteobacteria | Rhizobiales | DUNssu044 | unclassified |
| Otu00385 | 1.101910744 | -1.853058238 | 2.948406733 | -0.628494779 | 0.529679866 | NA | Proteobacteria | Alphaproteobacteria | Rhizobiales | Rhizobiaceae | Kaistia |
| Otu00404 | 0.299836238 | -1.303329804 | 2.954456999 | -0.441140218 | 0.659111489 | NA | Proteobacteria | Alphaproteobacteria | Rhizobiales | Rhizobiaceae | Kaistia |
| Otu00201 | 1.479043734 | -3.42867093 | 1.494811121 | -2.293715161 | 0.021806865 | NA | Proteobacteria | Alphaproteobacteria | Rhizobiales | Hyphomicrobiaceae | Devosia |
| Otu00327 | 0.509079216 | 0.508109381 | 2.850073066 | 0.178279423 | 0.858503538 | NA | Proteobacteria | Alphaproteobacteria | Rhizobiales | Hyphomicrobiaceae | unclassified |
| Otu00571 | 0.122874083 | -1.668347639 | 2.970377038 | -0.561661909 | 0.574346391 | NA | Proteobacteria | Alphaproteobacteria | Rhizobiales | Hyphomicrobiaceae | Devosia |
| Otu00295 | 1.042138784 | -2.416872114 | 2.106605475 | -1.147282746 | 0.251264783 | NA | Proteobacteria | Alphaproteobacteria | Rhizobiales | Hyphomicrobiaceae | Devosia |
| Otu00583 | 0.120101065 | -1.749126831 | 2.962810692 | -0.590360645 | 0.554948889 | NA | Proteobacteria | Alphaproteobacteria | Rhizobiales | Rhizobiales_Incertae_Sedis | Nordella |
| Otu01246 | 0.032439084 | -0.97440408 | 2.971671678 | -0.327897623 | 0.742989071 | NA | Proteobacteria | Alphaproteobacteria | Rhizobiales | Rhizobiales_Incertae_Sedis | Nordella |
| Otu00517 | 0.239226705 | -2.230666087 | 2.964391282 | -0.752487062 | 0.451758207 | NA | Proteobacteria | Alphaproteobacteria | Rhizobiales | unclassified | unclassified |
| Otu00293 | 0.936915965 | -1.085807492 | 2.206844842 | -0.492018048 | 0.622706585 | NA | Proteobacteria | Alphaproteobacteria | Rhizobiales | Phyllobacteriaceae | unclassified |
| Otu00638 | 0.153865599 | -0.472391936 | 2.96057636 | -0.159560801 | 0.873227059 | NA | Proteobacteria | Alphaproteobacteria | Rhizobiales | unclassified | unclassified |
| Otu01018 | 0.436450737 | -2.087889966 | 2.965742025 | -0.704002556 | 0.481431182 | NA | Proteobacteria | Alphaproteobacteria | Rhizobiales | unclassified | unclassified |
| Otu01239 | 0.348793625 | -1.733801178 | 2.969804094 | -0.583809949 | 0.559348184 | NA | Proteobacteria | Alphaproteobacteria | Rhizobiales | D05-2 | unclassified |
| Otu00376 | 0.313650486 | -0.221424417 | 2.948138587 | -0.075106516 | 0.940129964 | NA | Proteobacteria | Alphaproteobacteria | Rhizobiales | Rhizobiales_Incertae_Sedis | Alsobacter |
| Otu01021 | 0.326148423 | -0.414636839 | 2.97077106 | -0.139572128 | 0.888998064 | NA | Proteobacteria | Alphaproteobacteria | Rhizobiales | alphaI_cluster | unclassified |
| Otu00654 | 0.373829147 | -2.249503676 | 2.959679051 | -0.760049869 | 0.447224776 | NA | Proteobacteria | Alphaproteobacteria | Rhizobiales | Xanthobacteraceae | Labrys |
| Otu00954 | 0.049672009 | -1.362851668 | 2.968840124 | -0.459051889 | 0.646196904 | NA | Proteobacteria | Alphaproteobacteria | Rhizobiales | unclassified | unclassified |
| Otu00042 | 0.860211736 | 0.149139449 | 2.27911805 | 0.065437351 | 0.947825786 | NA | Proteobacteria | Alphaproteobacteria | Rhizobiales | Methylobacteriaceae | Methylobacterium |
| Otu00966 | 0.234246363 | -1.122650145 | 2.970462242 | -0.377937861 | 0.705476756 | NA | Proteobacteria | Alphaproteobacteria | Rhizobiales | Methylobacteriaceae | Microvirga |
| Otu00617 | 0.1365442 | -2.019160588 | 2.962202064 | -0.681641746 | 0.495465511 | NA | Proteobacteria | Alphaproteobacteria | Rhizobiales | Methylobacteriaceae | Microvirga |
| Otu00215 | 1.05431967 | -3.931250978 | 2.04504038 | -1.922334159 | 0.054563724 | NA | Proteobacteria | Alphaproteobacteria | Rhizobiales | Methylobacteriaceae | Microvirga |
| Otu00741 | 0.08306861 | -1.466735455 | 2.966051621 | -0.494507731 | 0.620947648 | NA | Proteobacteria | Alphaproteobacteria | Rhizobiales | unclassified | unclassified |
| Otu01368 | 0.227483482 | -1.647630021 | 2.969117903 | -0.554922396 | 0.578947742 | NA | Proteobacteria | Alphaproteobacteria | Rhizobiales | unclassified | unclassified |
| Otu00660 | 0.090642194 | -1.736081676 | 2.964581653 | -0.585607644 | 0.558139211 | NA | Proteobacteria | Alphaproteobacteria | Rhizobiales | Bradyrhizobiaceae | Bosea |
| Otu00315 | 0.330573906 | -0.478142701 | 2.964944319 | -0.161265322 | 0.871884435 | NA | Proteobacteria | Alphaproteobacteria | Rhizobiales | Beijerinckiaceae | Chelatococcus |
| Otu01325 | 0.096969637 | -1.05396859 | 2.971679105 | -0.354671064 | 0.722836031 | NA | Proteobacteria | Alphaproteobacteria | Rhizobiales | Methylobacteriaceae | Methylobacterium |
| Otu00799 | 0.016895746 | -0.803791054 | 2.973710717 | -0.270299007 | 0.786930229 | NA | Proteobacteria | Alphaproteobacteria | Rhizobiales | Methylobacteriaceae | Methylobacterium |
| Otu00330 | 0.638359954 | -1.065851703 | 2.960095589 | -0.360073407 | 0.718792239 | NA | Proteobacteria | Alphaproteobacteria | Rhizobiales | Methylobacteriaceae | Methylobacterium |
| Otu00713 | 0.16313929 | -1.790028161 | 2.969209579 | -0.602863528 | 0.546599481 | NA | Proteobacteria | Alphaproteobacteria | Rhizobiales | unclassified | unclassified |
| Otu00482 | 0.351254849 | -1.569103029 | 2.96295267 | -0.529574112 | 0.596407247 | NA | Proteobacteria | Alphaproteobacteria | Rhizobiales | unclassified | unclassified |
| Otu00402 | 0.267173844 | -0.770362904 | 2.950928569 | -0.261057795 | 0.794047938 | NA | Proteobacteria | Alphaproteobacteria | Rhizobiales | unclassified | unclassified |
| Otu00453 | 0.246913902 | -2.206725068 | 2.96005617 | -0.745501079 | 0.455968859 | NA | Proteobacteria | Alphaproteobacteria | Rhizobiales | Methylocystaceae | unclassified |

| | | | | | | | | | | | |
|---|---|---|---|---|---|---|---|---|---|---|---|
| Otu00365 | 0.341486554 | -1.572339269 | 2.949497892 | -0.533087097 | 0.59397329 | NA | Proteobacteria | Alphaproteobacteria | Rhizobiales | unclassified | unclassified |
| Otu00319 | 1.535985119 | -3.053276724 | 2.941062078 | -1.038154464 | 0.299198149 | NA | Proteobacteria | Alphaproteobacteria | Rhizobiales | Rhizobiaceae | unclassified |
| Otu00569 | 0.365947552 | 0.360000958 | 2.960724586 | 0.12159218 | 0.903222007 | NA | Proteobacteria | Alphaproteobacteria | Rhizobiales | Rhizobiaceae | Shinella |
| Otu00229 | 1.016970848 | -1.499271157 | 1.564169668 | -0.958509289 | 0.33780601 | NA | Proteobacteria | Alphaproteobacteria | unclassified | unclassified | unclassified |
| Otu00050 | 0.938457879 | -1.07898155 | 2.327485367 | -0.463582528 | 0.642946875 | NA | Proteobacteria | Alphaproteobacteria | Rhizobiales | Phyllobacteriaceae | Mesorhizobium |
| Otu00635 | 0.185567834 | -0.045969922 | 2.955850923 | -0.015552179 | 0.987591657 | NA | Proteobacteria | Alphaproteobacteria | Rhizobiales | unclassified | unclassified |
| Otu00664 | 0.180113868 | -1.672482394 | 2.965567875 | -0.563966992 | 0.572776596 | NA | Proteobacteria | Alphaproteobacteria | Rhizobiales | Xanthobacteraceae | unclassified |
| Otu00123 | 1.742650996 | -2.82734361 | 1.605534465 | -1.760998391 | 0.078238675 | NA | Proteobacteria | Alphaproteobacteria | Rhizobiales | Xanthobacteraceae | Pseudolabrys |
| Otu01134 | 0.176960215 | -1.053968594 | 2.971679105 | -0.354671065 | 0.72283603 | NA | Proteobacteria | Alphaproteobacteria | Rhizobiales | Xanthobacteraceae | Pseudolabrys |
| Otu00336 | 0.949970046 | -1.109124243 | 2.768685941 | -0.400595902 | 0.688717663 | NA | Proteobacteria | Alphaproteobacteria | Rhizobiales | unclassified | unclassified |
| Otu00224 | 1.028606566 | -3.842195409 | 2.941524328 | -1.306191954 | 0.191487288 | NA | Proteobacteria | Alphaproteobacteria | Rhizobiales | unclassified | unclassified |
| Otu00894 | 0.059597784 | -1.22646528 | 2.968571144 | -0.413150038 | 0.679496691 | NA | Proteobacteria | Alphaproteobacteria | Rhizobiales | Bradyrhizobiaceae | unclassified |
| Otu00241 | 0.814124485 | -2.339488854 | 2.755145547 | -0.849134397 | 0.395806512 | NA | Proteobacteria | Alphaproteobacteria | Rhizobiales | Bradyrhizobiaceae | Tardiphaga |
| Otu01171 | 0.221606809 | -1.218059852 | 2.971330285 | -0.409937548 | 0.681851761 | NA | Proteobacteria | Alphaproteobacteria | Rhizobiales | unclassified | unclassified |
| Otu00444 | 0.266399611 | -1.597670676 | 2.959320144 | -0.539877606 | 0.589281442 | NA | Proteobacteria | Alphaproteobacteria | Rhizobiales | Rhizobiales_Incertae_Sedis | Agaricicola |
| Otu00599 | 0.147758757 | -2.276298614 | 2.962174645 | -0.768455235 | 0.442216776 | NA | Proteobacteria | Alphaproteobacteria | Rhizobiales | Rhizobiales_Incertae_Sedis | Agaricicola |
| Otu01000 | 0.035934706 | -1.268194089 | 2.972752726 | -0.42660598 | 0.669666342 | NA | Proteobacteria | Alphaproteobacteria | Rhizobiales | Rhizobiales_Incertae_Sedis | Agaricicola |
| Otu00686 | 0.66393422 | -2.92810627 | 2.957538287 | -0.990048475 | 0.322150426 | NA | Proteobacteria | Alphaproteobacteria | Rhizobiales | JG34-KF-361 | unclassified |
| Otu00481 | 0.558165284 | -3.263181559 | 2.953898434 | -1.104703372 | 0.269288142 | NA | Proteobacteria | Alphaproteobacteria | Rhizobiales | JG34-KF-361 | unclassified |
| Otu00888 | 0.169883613 | -2.024005747 | 2.966291884 | -0.682335328 | 0.495026939 | NA | Proteobacteria | Alphaproteobacteria | Rhizobiales | JG34-KF-361 | unclassified |
| Otu00979 | 0.047256557 | -0.465613831 | 2.972958625 | -0.156616317 | 0.875547242 | NA | Proteobacteria | Alphaproteobacteria | Rhizobiales | JG34-KF-361 | unclassified |
| Otu00732 | 0.344795976 | -0.019951657 | 2.960818535 | -0.006738561 | 0.994623447 | NA | Proteobacteria | Alphaproteobacteria | Rhizobiales | JG34-KF-361 | unclassified |
| Otu00806 | 0.081953523 | -0.635553806 | 2.969674586 | -0.214014629 | 0.830535649 | NA | Proteobacteria | Alphaproteobacteria | Rhodobacterales | Rhodobacteraceae | unclassified |
| Otu01179 | 0.022453958 | -1.310577566 | 2.971834587 | -0.4409995 | 0.659213359 | NA | Proteobacteria | Alphaproteobacteria | unclassified | unclassified | unclassified |
| Otu00533 | 0.151117579 | -2.304834483 | 2.961045026 | -0.77838549 | 0.43634179 | NA | Proteobacteria | Alphaproteobacteria | Rhodobacterales | Rhodobacteraceae | unclassified |
| Otu00343 | 0.441603765 | -1.841841924 | 2.947343031 | -0.624916036 | 0.532026167 | NA | Proteobacteria | Alphaproteobacteria | unclassified | unclassified | unclassified |
| Otu00199 | 0.985851321 | -4.221662058 | 2.94667851 | -1.432684985 | 0.151947882 | NA | Proteobacteria | Alphaproteobacteria | Rhodobacterales | Rhodobacteraceae | unclassified |
| Otu00408 | 0.420862094 | -2.988189178 | 2.955252994 | -1.011144963 | 0.311947053 | NA | Proteobacteria | Alphaproteobacteria | Rhodobacterales | Rhodobacteraceae | unclassified |
| Otu00216 | 1.151804919 | -1.784642618 | 2.537960122 | -0.703179929 | 0.481943626 | NA | Proteobacteria | Alphaproteobacteria | Rhodobacterales | Rhodobacteraceae | unclassified |
| Otu01094 | 0.227483482 | -1.647630021 | 2.969117903 | -0.554922396 | 0.578947742 | NA | Proteobacteria | Alphaproteobacteria | Rhodobacterales | Rhodobacteraceae | unclassified |
| Otu00918 | 0.062581995 | -0.681728411 | 2.96970481 | -0.229561002 | 0.818432914 | NA | Proteobacteria | Alphaproteobacteria | Rhodobacterales | Rhodobacteraceae | unclassified |
| Otu00860 | 0.064878169 | -0.934805447 | 2.967173198 | -0.315049168 | 0.752724323 | NA | Proteobacteria | Alphaproteobacteria | Rhodobacterales | Rhodobacteraceae | Paracocccus |
| Otu00251 | 0.660404448 | -1.508863766 | 2.937946119 | -0.513577753 | 0.607547237 | NA | Proteobacteria | Alphaproteobacteria | Rhodobacterales | Rhodobacteraceae | Sedimentitalea |
| Otu00472 | 0.936357804 | -3.641388502 | 2.950210049 | -1.234281099 | 0.217098176 | NA | Proteobacteria | Alphaproteobacteria | Rhodobacterales | Rhodobacteraceae | unclassified |
| Otu00505 | 0.385692465 | -1.050407499 | 2.955350966 | -0.355425637 | 0.722270746 | NA | Proteobacteria | Alphaproteobacteria | Rhodobacterales | Rhodobacteraceae | unclassified |
| Otu00352 | 0.719853647 | -2.740939482 | 2.947351992 | -0.929966794 | 0.352388278 | NA | Proteobacteria | Alphaproteobacteria | Rhodobacterales | Rhodobacteraceae | unclassified |
| Otu00982 | 0.188976126 | -0.455753714 | 2.970356519 | -0.153434011 | 0.878056026 | NA | Proteobacteria | Alphaproteobacteria | Caulobacterales | Hyphomonadaceae | Hirschia |
| Otu01269 | 0.052525718 | -1.034877249 | 2.970871604 | -0.348341291 | 0.727583888 | NA | Proteobacteria | Alphaproteobacteria | Rhizobiales | Rhizobiales_Incertae_Sedis | Rhizomicrobium |
| Otu01304 | 0.070505569 | -0.985418248 | 2.971523153 | -0.331620586 | 0.740175773 | NA | Proteobacteria | Alphaproteobacteria | Rhizobiales | Rhizobiales_Incertae_Sedis | Rhizomicrobium |
| Otu00762 | 0.103721297 | -0.17145938 | 2.969822462 | -0.057733882 | 0.953960605 | NA | Proteobacteria | Alphaproteobacteria | Sphingomonadales | Sphingomonadaceae | Zymomonas |
| Otu00274 | 1.328046068 | -2.327544635 | 2.695457672 | -0.863506283 | 0.387859166 | NA | Proteobacteria | Alphaproteobacteria | Sphingomonadales | Sphingomonadaceae | Sphingomonas |
| Otu00495 | 0.153771772 | 0.642103755 | 2.963871394 | 0.216643595 | 0.828486108 | NA | Proteobacteria | Alphaproteobacteria | Sphingomonadales | unclassified | unclassified |
| Otu00313 | 0.745508768 | 0.475855615 | 2.937901599 | 0.161971257 | 0.871328488 | NA | Proteobacteria | Alphaproteobacteria | Sphingomonadales | Sphingomonadaceae | Sphingomonas |
| Otu00288 | 0.873824554 | -3.713627617 | 2.75600984 | -1.347465297 | 0.177830423 | NA | Proteobacteria | Alphaproteobacteria | Sphingomonadales | Sphingomonadaceae | Sphingomonas |
| Otu00491 | 1.684377593 | -3.440724087 | 2.951660546 | -1.165690984 | 0.243739407 | NA | Proteobacteria | Alphaproteobacteria | Sphingomonadales | Ellin6055 | unclassified |
| Otu00416 | 0.410569906 | -1.029551548 | 2.963805441 | -0.347374876 | 0.728309706 | NA | Proteobacteria | Alphaproteobacteria | Sphingomonadales | Sphingomonadaceae | Sphingomonas |
| Otu00957 | 0.092273343 | -0.362495958 | 2.971497589 | -0.121990998 | 0.902906147 | NA | Proteobacteria | Alphaproteobacteria | Sphingomonadales | Sphingomonadaceae | unclassified |
| Otu00305 | 0.355573108 | 0.503039449 | 2.961619849 | 0.169852808 | 0.865125895 | NA | Proteobacteria | Alphaproteobacteria | Sphingomonadales | Sphingomonadaceae | Sphingomonas |
| Otu00475 | 0.596521025 | -1.720468574 | 2.955790301 | -0.582067197 | 0.56052142 | NA | Proteobacteria | Alphaproteobacteria | Sphingomonadales | Sphingomonadaceae | unclassified |
| Otu00246 | 0.725681185 | -1.47074846 | 2.658565053 | -0.553211387 | 0.580118671 | NA | Proteobacteria | Alphaproteobacteria | Sphingomonadales | Sphingomonadaceae | Sphingomonas |
| Otu00550 | 0.118171202 | -0.061551734 | 2.960940401 | -0.0207879 | 0.98341485 | NA | Proteobacteria | Alphaproteobacteria | Sphingomonadales | Sphingomonadaceae | Sphingomonas |
| Otu00810 | 0.095844546 | -0.439719775 | 2.963653557 | -0.148370842 | 0.88205011 | NA | Proteobacteria | Alphaproteobacteria | Sphingomonadales | unclassified | unclassified |
| Otu00562 | 0.133256301 | 0.5377976 | 2.964168121 | 0.181432894 | 0.856027802 | NA | Proteobacteria | Alphaproteobacteria | Sphingomonadales | Sphingomonadaceae | unclassified |
| Otu00618 | 0.928467933 | -3.613520813 | 2.950463983 | -1.224729681 | 0.220677087 | NA | Proteobacteria | Alphaproteobacteria | Sphingomonadales | Erythrobacteraceae | Altererythrobacter |
| Otu00831 | 0.208806362 | -1.424399968 | 2.968242215 | -0.479879964 | 0.631312749 | NA | Proteobacteria | Alphaproteobacteria | Sphingomonadales | Erythrobacteraceae | Porphyrobacter |
| Otu00541 | 0.498523181 | 0.631299227 | 2.948029723 | 0.214142762 | 0.83043573 | NA | Proteobacteria | Alphaproteobacteria | Sphingomonadales | Sphingomonadaceae | Novosphingobium |
| Otu00651 | 0.679350956 | -2.604942207 | 2.956682102 | -0.881035606 | 0.378298548 | NA | Planctomycetes | Planctomycetacia | Planctomycetales | Planctomycetaceae | Singulisphaera |
| Otu01330 | 0.055517327 | -0.578806138 | 2.974205817 | -0.194608636 | 0.845699341 | NA | Planctomycetes | Planctomycetacia | Planctomycetales | Planctomycetaceae | unclassified |
| Otu00938 | 0.042699282 | -1.553349197 | 2.968972977 | -0.523194118 | 0.600839175 | NA | Planctomycetes | Planctomycetacia | Planctomycetales | Planctomycetaceae | unclassified |
| Otu00682 | 0.094075017 | -1.992940634 | 2.96402932 | -0.672375479 | 0.501344689 | NA | Planctomycetes | Planctomycetacia | Planctomycetales | Planctomycetaceae | Singulisphaera |
| Otu01158 | 0.023436115 | -1.301141594 | 2.972118452 | -0.43778255 | 0.661543922 | NA | Planctomycetes | Planctomycetacia | Planctomycetales | Planctomycetaceae | Singulisphaera |
| Otu01262 | 0.066613295 | -0.410818138 | 2.973250222 | -0.138171397 | 0.89010496 | NA | Planctomycetes | Planctomycetacia | Planctomycetales | Planctomycetaceae | Singulisphaera |
| Otu01015 | 0.02098545 | -0.925089602 | 2.973260564 | -0.311136405 | 0.755696927 | NA | Planctomycetes | Planctomycetacia | Planctomycetales | Planctomycetaceae | Singulisphaera |
| Otu00428 | 0.275131809 | 0.421839964 | 2.952348327 | 0.142882857 | 0.886382698 | NA | Planctomycetes | Planctomycetacia | Planctomycetales | Planctomycetaceae | Singulisphaera |
| Otu00532 | 0.214781081 | -1.707791983 | 2.965504635 | -0.575885791 | 0.56469238 | NA | Planctomycetes | Planctomycetacia | Planctomycetales | Planctomycetaceae | Singulisphaera |
| Otu00847 | 0.104425843 | -1.290153634 | 2.969405096 | -0.434482192 | 0.663938324 | NA | Planctomycetes | Planctomycetacia | Planctomycetales | Planctomycetaceae | Singulisphaera |
| Otu00342 | 0.62007824 | -2.039402421 | 2.954751203 | -0.690211216 | 0.490061371 | NA | Planctomycetes | Planctomycetacia | Planctomycetales | Planctomycetaceae | unclassified |
| Otu00479 | 0.433011138 | -3.159813001 | 2.954440746 | -1.069513073 | 0.284838541 | NA | Planctomycetes | Planctomycetacia | Planctomycetales | Planctomycetaceae | unclassified |
| Otu00462 | 0.765701247 | -3.047764471 | 2.952250917 | -1.03235279 | 0.301906879 | NA | Planctomycetes | Planctomycetacia | Planctomycetales | Planctomycetaceae | unclassified |
| Otu00538 | 0.567536049 | -0.259141797 | 2.959233105 | -0.087570593 | 0.930217976 | NA | Planctomycetes | Planctomycetacia | Planctomycetales | Planctomycetaceae | unclassified |
| Otu00825 | 0.293553575 | -0.465441483 | 2.966803115 | -0.156883172 | 0.875336923 | NA | Planctomycetes | Planctomycetacia | Planctomycetales | Planctomycetaceae | Singulisphaera |
| Otu00388 | 0.344988573 | -2.045223549 | 2.962303787 | -0.690416546 | 0.489932274 | NA | Planctomycetes | Planctomycetacia | Planctomycetales | Planctomycetaceae | Singulisphaera |
| Otu00845 | 0.075558789 | -1.858830864 | 2.965469349 | -0.626825182 | 0.530773835 | NA | Planctomycetes | Planctomycetacia | Planctomycetales | Planctomycetaceae | Singulisphaera |
| Otu00474 | 0.186008059 | -2.257025381 | 2.959297521 | -0.762689579 | 0.445648547 | NA | Planctomycetes | Planctomycetacia | Planctomycetales | Planctomycetaceae | Singulisphaera |
| Otu01164 | 0.031155782 | -1.112121031 | 2.972105042 | -0.374186314 | 0.708265705 | NA | Planctomycetes | Planctomycetacia | Planctomycetales | Planctomycetaceae | Singulisphaera |
| Otu01052 | 0.088274856 | -1.217007693 | 2.96867684 | -0.409949536 | 0.681842966 | NA | Planctomycetes | Planctomycetacia | Planctomycetales | Planctomycetaceae | Zavarzinella |
| Otu00796 | 0.16786254 | -0.492570232 | 2.970640169 | -0.165812823 | 0.868304255 | NA | Planctomycetes | Planctomycetacia | Planctomycetales | Planctomycetaceae | Planctomyces |
| Otu00711 | 0.065764376 | -0.095545391 | 2.970696668 | -0.032162621 | 0.974342365 | NA | Planctomycetes | Planctomycetacia | Planctomycetales | Planctomycetaceae | Planctomyces |
| Otu00984 | 0.242311173 | -0.790058108 | 2.970391694 | -0.265977753 | 0.790256343 | NA | Planctomycetes | Planctomycetacia | Planctomycetales | Planctomycetaceae | Planctomyces |
| Otu01168 | 0.138687791 | -0.541194458 | 2.971032268 | -0.182157045 | 0.855459482 | NA | Planctomycetes | Planctomycetacia | Planctomycetales | Planctomycetaceae | Planctomyces |
| Otu00398 | 0.243050621 | -2.480808691 | 2.956728205 | -0.839038464 | 0.401447726 | NA | Planctomycetes | Planctomycetacia | Planctomycetales | Planctomycetaceae | Pir4_lineage |
| Otu00650 | 0.596337935 | -2.960537161 | 2.957243664 | -1.001113705 | 0.31677184 | NA | Planctomycetes | Planctomycetacia | Planctomycetales | Planctomycetaceae | Pir4_lineage |
| Otu00761 | 0.533058867 | -1.114586185 | 2.965517389 | -0.375848811 | 0.707029298 | NA | Planctomycetes | Planctomycetacia | Planctomycetales | Planctomycetaceae | unclassified |
| Otu00813 | 0.316685195 | -2.337396279 | 2.962891444 | -0.788890286 | 0.430176134 | NA | Planctomycetes | Planctomycetacia | Planctomycetales | Planctomycetaceae | unclassified |
| Otu00351 | 0.301587062 | -2.668397448 | 2.954751347 | -0.903086972 | 0.366479738 | NA | Planctomycetes | Planctomycetacia | Planctomycetales | Planctomycetaceae | Pir4_lineage |
| Otu00172 | 0.498807272 | -2.091789804 | 2.946141708 | -0.710009909 | 0.477697992 | NA | Planctomycetes | Planctomycetacia | Planctomycetales | Planctomycetaceae | Pir4_lineage |
| Otu01085 | 0.037779395 | -1.507802737 | 2.96938731 | -0.507782441 | 0.611605925 | NA | Planctomycetes | Planctomycetacia | Planctomycetales | Planctomycetaceae | Blastopirellula |
| Otu01192 | 0.107590428 | -1.053968576 | 2.971679106 | -0.354671059 | 0.722836035 | NA | Planctomycetes | Planctomycetacia | Planctomycetales | Planctomycetaceae | Rhodopirellula |
| Otu00652 | 0.260149738 | 0.696555295 | 2.953548223 | 0.235836777 | 0.813559329 | NA | Planctomycetes | Planctomycetacia | Planctomycetales | Planctomycetaceae | Pirellula |
| Otu01198 | 0.159089537 | -1.658176429 | 2.968993827 | -0.558497769 | 0.576504528 | NA | Planctomycetes | Planctomycetacia | Planctomycetales | Planctomycetaceae | Pirellula |
| Otu00412 | 0.222703273 | -1.308814933 | 2.953586356 | -0.443127363 | 0.657673614 | NA | Acidobacteria | Acidobacteria | Subgroup_6 | unclassified | unclassified |
| Otu00493 | 0.521137644 | 0.642884134 | 2.950235581 | 0.217909423 | 0.827499685 | NA | Acidobacteria | Acidobacteria | Subgroup_6 | unclassified | unclassified |
| Otu00372 | 0.299650642 | -0.285884774 | 2.949155236 | -0.096937852 | 0.922775749 | NA | Acidobacteria | Acidobacteria | Subgroup_6 | unclassified | unclassified |
| Otu01082 | 0.073825564 | -0.801916578 | 2.972741911 | -0.269756542 | 0.787347558 | NA | Acidobacteria | Acidobacteria | Subgroup_6 | unclassified | unclassified |
| Otu00390 | 0.739792078 | 2.215355824 | 2.947229126 | 0.751674108 | 0.452247065 | NA | Acidobacteria | Acidobacteria | Subgroup_6 | unclassified | unclassified |
| Otu00700 | 0.152708327 | 0.284003616 | 2.960953506 | 0.09591627 | 0.923587072 | NA | Acidobacteria | Acidobacteria | Subgroup_6 | unclassified | unclassified |
| Otu01204 | 0.07292198 | -1.034877258 | 2.970871604 | -0.348341294 | 0.727583886 | NA | Acidobacteria | Acidobacteria | Subgroup_6 | unclassified | unclassified |
| Otu00803 | 0.134634424 | -1.064931915 | 2.963028742 | -0.359406542 | 0.719290981 | NA | Acidobacteria | Acidobacteria | Subgroup_6 | unclassified | unclassified |
| Otu00324 | 0.69343472 | -0.401852684 | 2.943042094 | -0.136543302 | 0.891391795 | NA | Acidobacteria | Acidobacteria | Subgroup_4 | Unknown_Family | Blastocatella |
| Otu00520 | 0.188071066 | 0.640211158 | 2.964500309 | 0.215959214 | 0.82901954 | NA | Acidobacteria | Acidobacteria | Subgroup_4 | Unknown_Family | Blastocatella |
| Otu00391 | 1.342355662 | -2.458671515 | 2.935319175 | -0.837616412 | 0.402246172 | NA | Acidobacteria | Acidobacteria | Subgroup_4 | Unknown_Family | Blastocatella |
| Otu00471 | 0.195027918 | -2.075914257 | 2.95822384 | -0.701743468 | 0.48283916 | NA | Acidobacteria | Acidobacteria | Subgroup_4 | Unknown_Family | Blastocatella |
| Otu00792 | 0.087011717 | -1.428886645 | 2.966462759 | -0.481680291 | 0.630033079 | NA | Acidobacteria | Acidobacteria | Subgroup_4 | RB41 | unclassified |
| Otu00420 | 0.537703124 | 1.40032196 | 2.946476088 | 0.475253122 | 0.634606568 | NA | Acidobacteria | Acidobacteria | Subgroup_4 | RB41 | unclassified |
| Otu00906 | 0.027066078 | -1.016410401 | 2.971813802 | -0.342016852 | 0.73233821 | NA | unclassified | unclassified | unclassified | unclassified | unclassified |
| Otu01320 | 0.004274925 | -0.822536931 | 2.974646544 | -0.276515855 | 0.782151884 | NA | Verrucomicrobia | Opitutae | Opitutales | Opitutaceae | Opitutus |
| Otu00526 | 0.279034516 | -0.127050269 | 2.950468499 | -0.043061049 | 0.965652869 | NA | Verrucomicrobia | Spartobacteria | Chthoniobacterales | Xiphinematobacteraceae | Candidatus_Xiphinematobacter |
| Otu00644 | 0.102657563 | -1.288365281 | 2.962506709 | -0.434890249 | 0.663642093 | NA | Verrucomicrobia | Spartobacteria | Chthoniobacterales | FukuN18_freshwater_group | unclassified |
| Otu00238 | 0.986123808 | -2.872135786 | 2.956243105 | -0.971549255 | 0.331274838 | NA | Verrucomicrobia | Spartobacteria | Chthoniobacterales | Xiphinematobacteraceae | Candidatus_Xiphinematobacter |
| Otu00521 | 0.159906538 | -2.126567026 | 2.964849845 | -0.717259604 | 0.473213928 | NA | Verrucomicrobia | Spartobacteria | Chthoniobacterales | Xiphinematobacteraceae | Candidatus_Xiphinematobacter |
| Otu01035 | 0.489069838 | -0.249239159 | 2.970502936 | -0.0839047 | 0.933132202 | NA | Verrucomicrobia | Spartobacteria | Chthoniobacterales | unclassified | unclassified |
| Otu00631 | 0.371680268 | -0.500071248 | 2.967769338 | -0.168500713 | 0.866189383 | NA | Verrucomicrobia | Spartobacteria | Chthoniobacterales | DA101_soil_group | unclassified |
| Otu00760 | 0.070646973 | -0.879394606 | 2.96636854 | -0.29645494 | 0.766882674 | NA | Verrucomicrobia | Spartobacteria | Chthoniobacterales | DA101_soil_group | unclassified |
| Otu00175 | 0.156386457 | -1.345849471 | 2.962431851 | -0.454305631 | 0.649608864 | NA | Verrucomicrobia | Spartobacteria | Chthoniobacterales | DA101_soil_group | unclassified |
| Otu00202 | 1.169981868 | -2.115618578 | 1.776245324 | -1.191062152 | 0.233629186 | NA | Verrucomicrobia | Spartobacteria | Chthoniobacterales | DA101_soil_group | unclassified |
| Otu00256 | 1.083354641 | 0.562228966 | 2.563713459 | 0.219302576 | 0.826414355 | NA | Verrucomicrobia | Spartobacteria | Chthoniobacterales | DA101_soil_group | unclassified |
| Otu00197 | 0.977966829 | -3.672168284 | 2.944612948 | -1.247080125 | 0.21236812 | NA | Verrucomicrobia | Spartobacteria | Chthoniobacterales | Chthoniobacteraceae | Chthoniobacter |
| Otu00677 | 0.089692223 | -1.727009048 | 2.964689387 | -0.582526134 | 0.560212343 | NA | Verrucomicrobia | Spartobacteria | Chthoniobacterales | Chthoniobacteraceae | Chthoniobacter |
| Otu00944 | 0.07569309 | -1.168132106 | 2.97073795 | -0.393212773 | 0.694162337 | NA | Verrucomicrobia | Spartobacteria | Chthoniobacterales | Chthoniobacteraceae | Chthoniobacter |

| | | | | | | | | | | | | |
|---|---|---|---|---|---|---|---|---|---|---|---|---|
| Otu01213 | 0.191068772 | -1.053968591 | 2.971679105 | -0.354671064 | 0.722836031 | NA | Verrucomicrobia | Verrucomicrobiae | Verrucomicrobiales | Verrucomicrobiaceae | Prosthecobacter |
| Otu00261 | 1.426594283 | -3.576448801 | 1.707234563 | -2.094878394 | 0.036181804 | NA | Verrucomicrobia | Verrucomicrobiae | Verrucomicrobiales | Verrucomicrobiaceae | Luteolibacter |
| Otu00897 | 0.113836557 | -1.89537646 | 2.966460091 | -0.638935432 | 0.522864937 | NA | Verrucomicrobia | Verrucomicrobiae | Verrucomicrobiales | Verrucomicrobiaceae | Luteolibacter |
| Otu00749 | 0.085087398 | 0.133612808 | 2.964944871 | 0.04506418 | 0.964056153 | NA | Verrucomicrobia | Verrucomicrobiae | Verrucomicrobiales | Verrucomicrobiaceae | Haloferula |
| Otu00779 | 0.051242402 | -0.682796818 | 2.97445294 | -0.229553747 | 0.818438552 | NA | Verrucomicrobia | Verrucomicrobiae | Verrucomicrobiales | Verrucomicrobiaceae | Luteolibacter |
| Otu00544 | 1.414613712 | -3.224989471 | 2.950945817 | -1.09286638 | 0.274452472 | NA | Verrucomicrobia | Verrucomicrobiae | Verrucomicrobiales | Verrucomicrobiaceae | unclassified |
| Otu00171 | 0.320076833 | -1.591015188 | 2.964568635 | -0.536676793 | 0.591490889 | NA | Verrucomicrobia | OPB35_soil_group | unclassified | unclassified | unclassified |
| Otu01288 | 0.141768355 | -0.471044228 | 2.972551876 | -0.158464595 | 0.874090717 | NA | Verrucomicrobia | OPB35_soil_group | unclassified | unclassified | unclassified |
| Otu00430 | 0.306999231 | -1.898756095 | 2.95625811 | -0.642283598 | 0.520689062 | NA | Verrucomicrobia | OPB35_soil_group | unclassified | unclassified | unclassified |
| Otu00075 | 1.700948676 | -3.582339083 | 2.951420504 | -1.21376777 | 0.224836417 | NA | Firmicutes | Negativicutes | Selenomonadales | Veillonellaceae | unclassified |
| Otu00902 | 0.01248758 | -0.780816988 | 2.97414397 | -0.262535034 | 0.792908979 | NA | Firmicutes | unclassified | unclassified | unclassified | unclassified |
| Otu00035 | 0.019422142 | -1.259169883 | 2.972536871 | -0.423601098 | 0.671856754 | NA | Firmicutes | Negativicutes | Selenomonadales | Veillonellaceae | Veillonella |
| Otu00600 | 0.102800309 | -0.162942689 | 2.962370332 | -0.055004159 | 0.95613515 | NA | unclassified | unclassified | unclassified | unclassified | unclassified |
| Otu00225 | 0.076917306 | -0.259584747 | 2.970597078 | -0.087384704 | 0.930365727 | NA | Fusobacteria | Fusobacteriia | Fusobacteriales | Leptotrichiaceae | Streptobacillus |
| Otu00143 | 0.853635132 | -2.081499759 | 1.768732776 | -1.176831112 | 0.239262922 | NA | Fusobacteria | Fusobacteriia | Fusobacteriales | Fusobacteriaceae | unclassified |
| Otu00228 | 0.054248325 | -1.383562851 | 2.972138531 | -0.46551089 | 0.64156564 | NA | Tenericutes | Mollicutes | Entomoplasmatales | Entomoplasmatales_Incertae_Sedis | Candidatus_Hepatoplasma |
| Otu00629 | 0.236142206 | -2.384396504 | 2.961291385 | -0.805188073 | 0.420711158 | NA | Firmicutes | Erysipelotrichia | Erysipelotrichales | Erysipelotrichaceae | unclassified |
| Otu00508 | 0.19379078 | 0.726999474 | 2.958086759 | 0.245766785 | 0.80586277 | NA | Armatimonadetes | unclassified | unclassified | unclassified | unclassified |
| Otu00507 | 0.19379078 | 0.726999474 | 2.958086759 | 0.245766785 | 0.80586277 | NA | Armatimonadetes | unclassified | unclassified | unclassified | unclassified |
| Otu00165 | 0.089398975 | -1.537804969 | 2.971348915 | -0.517544392 | 0.604776188 | NA | Firmicutes | Bacilli | unclassified | unclassified | unclassified |
| Otu00915 | 0.058967378 | -0.667539376 | 2.972411741 | -0.224578367 | 0.822307295 | NA | Firmicutes | Bacilli | Lactobacillales | Enterococcaceae | Catellicoccus |
| Otu00896 | 0.073522174 | -1.159209347 | 2.970758515 | -0.390206522 | 0.696383839 | NA | Firmicutes | Bacilli | Lactobacillales | Lactobacillaceae | Lactobacillus |
| Otu00310 | 1.034127732 | -2.313014731 | 2.94053357 | -0.786596948 | 0.431517846 | NA | Firmicutes | Bacilli | Lactobacillales | Carnobacteriaceae | Dolosigranulum |
| Otu00168 | 0.818778412 | -3.947059457 | 2.948372927 | -1.33872463 | 0.180660335 | NA | Firmicutes | Bacilli | Lactobacillales | unclassified | unclassified |
| Otu00317 | 0.418958985 | -0.480179888 | 2.944571651 | -0.163072917 | 0.870461024 | NA | Firmicutes | Bacilli | Lactobacillales | Enterococcaceae | Vagococcus |
| Otu00357 | 0.650437942 | -3.639501122 | 2.950406866 | -1.23355906 | 0.217367256 | NA | Firmicutes | Bacilli | Lactobacillales | unclassified | unclassified |
| Otu00819 | 0.106216365 | -0.63555383 | 2.969674586 | -0.214014637 | 0.830535643 | NA | Firmicutes | Bacilli | Bacillales | Listeriaceae | Listeria |
| Otu00145 | 0.013922857 | -0.682796837 | 2.97445294 | -0.229553754 | 0.818438546 | NA | Firmicutes | Bacilli | Bacillales | Staphylococcaceae | Macrococcus |
| Otu00226 | 0.099072544 | -1.056618386 | 2.965077726 | -0.356354363 | 0.721575202 | NA | Firmicutes | Bacilli | Bacillales | Bacillaceae | Bacillus |
| Otu00603 | 0.1673978 | -2.254920721 | 2.96301648 | -0.761021998 | 0.446643928 | NA | Firmicutes | Bacilli | Bacillales | unclassified | unclassified |
| Otu00663 | 0.473625937 | -0.826203823 | 2.9618195 | -0.278951443 | 0.780282089 | NA | Firmicutes | Bacilli | Bacillales | Bacillaceae | unclassified |
| Otu00250 | 0.568680866 | -1.710006133 | 2.943025867 | -0.581036732 | 0.561215697 | NA | Firmicutes | Bacilli | Bacillales | Bacillaceae | unclassified |
| Otu00302 | 1.169358803 | -4.023360868 | 2.943833033 | -1.366708242 | 0.171716774 | NA | Firmicutes | Bacilli | Bacillales | Planococcaceae | Domibacillus |
| Otu00340 | 1.644397784 | 0.92728187 | 2.95339688 | 0.313971304 | 0.753542834 | NA | Firmicutes | Bacilli | Bacillales | Planococcaceae | unclassified |
| Otu00712 | 0.500700939 | -2.167251083 | 2.965346835 | -0.730859223 | 0.464865145 | NA | Firmicutes | Bacilli | Bacillales | Bacillaceae | unclassified |
| Otu00308 | 0.508954747 | -3.278814404 | 2.951775354 | -1.110794017 | 0.266657022 | NA | Firmicutes | Bacilli | Bacillales | unclassified | unclassified |
| Otu00234 | 0.765880814 | -2.445830003 | 2.940199453 | -0.831858533 | 0.4054888 | NA | Firmicutes | Bacilli | Bacillales | Bacillaceae | Bacillus |
| Otu00356 | 0.611716164 | -0.558167073 | 2.635836322 | -0.211760901 | 0.832293573 | NA | Firmicutes | Bacilli | Bacillales | Planococcaceae | unclassified |
| Otu00765 | 0.259922567 | -1.6012499 | 2.965499329 | -0.539959623 | 0.589224878 | NA | Firmicutes | Erysipelotrichia | Erysipelotrichales | Erysipelotrichaceae | Turicibacter |
| Otu00054 | 0.484696845 | -2.740673811 | 2.948338483 | -0.929565525 | 0.352596084 | NA | Firmicutes | Bacilli | Lactobacillales | Streptococcaceae | Lactococcus |
| Otu00128 | 0.004172948 | -1.009762316 | 2.974451443 | -0.339478501 | 0.734249291 | NA | Firmicutes | Bacilli | Lactobacillales | Leuconostocaceae | Weissella |
| Otu00757 | 0.0777283 | -0.987972279 | 2.966987712 | -0.332988328 | 0.739143093 | NA | Firmicutes | Bacilli | Bacillales | Paenibacillaceae | Paenibacillus |
| Otu01277 | 0.033186024 | -0.98541825 | 2.971523153 | -0.331620586 | 0.740175773 | NA | Firmicutes | Bacilli | Bacillales | Paenibacillaceae | Paenibacillus |
| Otu00446 | 0.390601615 | 0.768751902 | 2.947547549 | 0.260810687 | 0.794238502 | NA | Firmicutes | Bacilli | Bacillales | Paenibacillaceae | Paenibacillus |
| Otu00675 | 0.086704464 | -1.70438315 | 2.964961557 | -0.574841568 | 0.565398451 | NA | Firmicutes | Bacilli | Bacillales | Paenibacillaceae | Paenibacillus |
| Otu00490 | 0.156600634 | -1.347157025 | 2.957946386 | -0.455436593 | 0.648795174 | NA | Firmicutes | Bacilli | Bacillales | Paenibacillaceae | Paenibacillus |
| Otu00775 | 0.129917762 | -0.295971931 | 2.964650638 | -0.099833662 | 0.920476383 | NA | Firmicutes | Bacilli | Bacillales | Paenibacillaceae | Cohnella |
| Otu00566 | 0.433245484 | 0.122368153 | 2.956709202 | 0.041386604 | 0.966987692 | NA | Fibrobacteres | Fibrobacteria | Fibrobacterales | Fibrobacteraceae | possible_genus_04 |
| Otu00837 | 0.22179203 | -0.956299003 | 2.971353512 | -0.321839525 | 0.747574269 | NA | Actinobacteria | Coriobacteriia | Coriobacteriales | Coriobacteriaceae | unclassified |
| Otu01024 | 0.159089537 | -1.658176429 | 2.968993827 | -0.558497769 | 0.576504528 | NA | Acidobacteria | Acidobacteria | Subgroup_3 | unclassified | unclassified |
| Otu01265 | 0.069758629 | -0.974404078 | 2.971671678 | -0.327897622 | 0.742989071 | NA | Acidobacteria | Acidobacteria | Subgroup_3 | Unknown_Family | Candidatus_Solibacter |
| Otu00573 | 0.346421933 | -1.314028483 | 2.955654094 | -0.444581281 | 0.656622378 | NA | Acidobacteria | Acidobacteria | Subgroup_3 | SJA-149 | unclassified |
| Otu00728 | 0.32531893 | -1.874390321 | 2.963746313 | -0.632439528 | 0.527099711 | NA | Acidobacteria | Acidobacteria | Acidobacteriales | Acidobacteriaceae_(Subgroup_1) | unclassified |
| Otu00940 | 0.063834298 | -0.190064354 | 2.971719883 | -0.063957695 | 0.949003912 | NA | Armatimonadetes | Armatimonadia | Armatimonadales | unclassified | unclassified |
| Otu00776 | 0.085493712 | 0.187552348 | 2.967663119 | 0.063198665 | 0.949608308 | NA | Deinococcus-Thermus | Deinococci | Deinococcales | Trueperaceae | Truepera |
| Otu01146 | 0.139410687 | -0.471044217 | 2.972551876 | -0.158464591 | 0.87409072 | NA | Deinococcus-Thermus | Deinococci | Deinococcales | Trueperaceae | Truepera |
| Otu00361 | 0.432003745 | -2.422108802 | 2.949822892 | -0.821103127 | 0.411587529 | NA | Deinococcus-Thermus | Deinococci | Deinococcales | Deinococcaceae | Deinococcus |
| Otu00496 | 0.445727205 | -2.682340223 | 2.955362106 | -0.907618128 | 0.364080013 | NA | Chloroflexi | Caldilineae | Caldilineales | Caldilineaceae | unclassified |
| Otu00781 | 0.093429467 | -1.403588102 | 2.966438808 | -0.473155926 | 0.636101939 | NA | Chloroflexi | TK10 | unclassified | unclassified | unclassified |
| Otu00653 | 0.372384786 | -0.409273175 | 2.958812237 | -0.13832347 | 0.889984778 | NA | Chloroflexi | TK10 | unclassified | unclassified | unclassified |
| Otu00770 | 0.197615872 | -2.087276293 | 2.964354814 | -0.704124986 | 0.481354941 | NA | unclassified | unclassified | unclassified | unclassified | unclassified |
| Otu00859 | 0.646164932 | -2.660767131 | 2.960346543 | -0.898802587 | 0.368757822 | NA | Chloroflexi | Chloroflexia | Kallotenuales | AKIW781 | unclassified |
| Otu00978 | 0.210331939 | -1.389622003 | 2.96768077 | -0.468251847 | 0.639604499 | NA | Chloroflexi | Chloroflexia | Kallotenuales | AKIW781 | unclassified |
| Otu00529 | 0.121039117 | -2.020283033 | 2.962399229 | -0.681975277 | 0.495254584 | NA | Chloroflexi | Thermomicrobia | JG30-KF-CM45 | unclassified | unclassified |
| Otu00359 | 0.367000421 | -2.302321017 | 2.964213908 | -0.776705423 | 0.437332587 | NA | Chloroflexi | Thermomicrobia | JG30-KF-CM45 | unclassified | unclassified |
| Otu00321 | 1.021798227 | -3.570190539 | 2.947313333 | -1.211337288 | 0.225766163 | NA | Chloroflexi | Thermomicrobia | JG30-KF-CM45 | unclassified | unclassified |
| Otu00563 | 0.146190571 | -2.197217907 | 2.963431076 | -0.741443904 | 0.458424332 | NA | Chloroflexi | Thermomicrobia | JG30-KF-CM45 | unclassified | unclassified |
| Otu00589 | 0.477944242 | -2.127070621 | 2.958515928 | -0.718965411 | 0.472162232 | NA | Chloroflexi | Thermomicrobia | JG30-KF-CM45 | unclassified | unclassified |
| Otu00442 | 0.734079753 | -0.711990077 | 2.956376767 | -0.240831982 | 0.809685341 | NA | Chloroflexi | Thermomicrobia | JG30-KF-CM45 | unclassified | unclassified |
| Otu00868 | 0.101282656 | -1.78290028 | 2.968134693 | -0.600680381 | 0.548052888 | NA | Chloroflexi | Thermomicrobia | JG30-KF-CM45 | unclassified | unclassified |
| Otu00400 | 0.652565457 | -3.493298626 | 2.951774501 | -1.183457146 | 0.236628021 | NA | Chloroflexi | Thermomicrobia | JG30-KF-CM45 | unclassified | unclassified |
| Otu00560 | 0.473093316 | -0.10231169 | 2.963227014 | -0.034527118 | 0.972456818 | NA | unclassified | unclassified | unclassified | unclassified | unclassified |
| Otu00459 | 0.304725783 | -2.897559367 | 2.956345135 | -0.980115391 | 0.327029163 | NA | Chloroflexi | Thermomicrobia | JG30-KF-CM45 | unclassified | unclassified |
| Otu01347 | 0.284277478 | -1.73380117 | 2.969804094 | -0.583809947 | 0.559348185 | NA | Chloroflexi | KD4-96 | unclassified | unclassified | unclassified |
| Otu00815 | 0.441138524 | -2.020694044 | 2.962509584 | -0.68208861 | 0.495182922 | NA | Chloroflexi | KD4-96 | unclassified | unclassified | unclassified |
| Otu00516 | 0.349978763 | -2.923240071 | 2.956413332 | -0.988779221 | 0.322771173 | NA | Chloroflexi | KD4-96 | unclassified | unclassified | unclassified |
| Otu00518 | 0.397087629 | -3.002020085 | 2.956180333 | -1.015506413 | 0.309864491 | NA | Chloroflexi | KD4-96 | unclassified | unclassified | unclassified |
| Otu01415 | 0.072901815 | -0.410818129 | 2.973250222 | -0.138171395 | 0.890104962 | NA | Chloroflexi | Anaerolineae | Anaerolineales | Anaerolineaceae | unclassified |
| Otu01251 | 0.049382532 | -1.589643111 | 2.968477754 | -0.53550784 | 0.592298738 | NA | Chloroflexi | Anaerolineae | Anaerolineales | Anaerolineaceae | unclassified |
| Otu00341 | 0.676136434 | 1.232840005 | 2.943026728 | 0.418902076 | 0.6752877 | NA | Chloroflexi | unclassified | unclassified | unclassified | unclassified |
| Otu01208 | 0.139410687 | -0.471044217 | 2.972551876 | -0.158464591 | 0.87409072 | NA | Chloroflexi | Anaerolineae | Anaerolineales | Anaerolineaceae | unclassified |
| Otu01191 | 0.054457235 | -0.839917293 | 2.972683256 | -0.282545169 | 0.777525517 | NA | Chloroflexi | Anaerolineae | Anaerolineales | Anaerolineaceae | unclassified |
| Otu00487 | 0.181694147 | -2.466100313 | 2.959955425 | -0.833154544 | 0.404757575 | NA | Saccharibacteria | unclassified | unclassified | unclassified | unclassified |
| Otu00383 | 0.325864359 | -1.326253364 | 2.952386038 | -0.449214075 | 0.653277235 | NA | WCHB1-60 | unclassified | unclassified | unclassified | unclassified |
| Otu01264 | 0.060006504 | -0.818201041 | 2.973675975 | -0.275148015 | 0.783202524 | NA | Planctomycetes | Phycisphaerae | Phycisphaerales | Phycisphaeraceae | Phycisphaera |
| Otu01248 | 0.042174885 | -0.496146627 | 2.972812494 | -0.166894692 | 0.867452909 | NA | Planctomycetes | Phycisphaerae | WD2101_soil_group | unclassified | unclassified |
| Otu00548 | 0.103683039 | 0.07098 | 2.968820434 | 0.023908485 | 0.980925606 | NA | Planctomycetes | Phycisphaerae | WD2101_soil_group | unclassified | unclassified |
| Otu00294 | 0.037918662 | -0.650210713 | 2.97198799 | -0.218779724 | 0.826821642 | NA | Planctomycetes | Phycisphaerae | WD2101_soil_group | unclassified | unclassified |
| Otu00827 | 0.143407384 | -0.313042721 | 2.964695941 | -0.105590161 | 0.915907532 | NA | Planctomycetes | Phycisphaerae | WD2101_soil_group | unclassified | unclassified |
| Otu00289 | 0.549836934 | -2.124004468 | 2.955281323 | -0.718714814 | 0.472316654 | NA | Cyanobacteria | Cyanobacteria | SubsectionI | FamilyI | Synechococcus |
| Otu00345 | 0.323848449 | -2.965517901 | 2.954947508 | -1.003577185 | 0.315582456 | NA | Cyanobacteria | Cyanobacteria | SubsectionII | FamilyII | Pleurocapsa |
| Otu01364 | 0.227483482 | -1.647630021 | 2.969117903 | -0.554922396 | 0.578947742 | NA | unclassified | unclassified | unclassified | unclassified | unclassified |
| Otu00407 | 0.237810178 | -1.617376857 | 2.962882805 | -0.545879457 | 0.5851488 | NA | unclassified | unclassified | unclassified | unclassified | unclassified |
| Otu00575 | 0.137346778 | -1.659054322 | 2.962162153 | -0.560082209 | 0.575423365 | NA | unclassified | unclassified | unclassified | unclassified | unclassified |
| Otu00160 | 0.308663045 | -2.849159265 | 2.955540081 | -0.964006302 | 0.335042766 | NA | Spirochaetae | Spirochaetes | Spirochaetales | Brachyspiraceae | Brachyspira |
| Otu00823 | 0.085493712 | 0.187552348 | 2.967663119 | 0.063198665 | 0.949608308 | NA | Bacteroidetes | Cytophagia | Cytophagales | Cytophagaceae | Persicitalea |
| Otu00364 | 0.512199047 | 0.547069934 | 2.949642358 | 0.185469921 | 0.852860471 | NA | Bacteroidetes | Cytophagia | Cytophagales | Cytophagaceae | Dyadobacter |
| Otu00200 | 0.328247534 | 0.100291236 | 2.966207215 | 0.033811271 | 0.973027648 | NA | Bacteroidetes | Cytophagia | Cytophagales | Cytophagaceae | Spirosoma |
| Otu00619 | 0.050743438 | -0.338891287 | 2.971598654 | -0.114043425 | 0.90920337 | NA | Bacteroidetes | Cytophagia | Cytophagales | Cytophagaceae | Spirosoma |
| Otu00543 | 0.151803762 | 0.532267256 | 2.965484227 | 0.179487468 | 0.857554956 | NA | Bacteroidetes | Cytophagia | Cytophagales | Cytophagaceae | Spirosoma |
| Otu00795 | 0.153146527 | 0.256234566 | 2.967285124 | 0.0863532 | 0.931185649 | NA | Bacteroidetes | Cytophagia | Cytophagales | Cytophagaceae | Dyadobacter |
| Otu00580 | 0.17547917 | 0.265255843 | 2.967114664 | 0.089398582 | 0.928765151 | NA | Bacteroidetes | Cytophagia | Cytophagales | Cytophagaceae | Hymenobacter |
| Otu00411 | 0.247329593 | 0.219316856 | 2.966527206 | 0.073930506 | 0.941065682 | NA | Bacteroidetes | Cytophagia | Cytophagales | Cytophagaceae | Hymenobacter |
| Otu00739 | 0.29560491 | -0.168961767 | 2.963059324 | -0.057022742 | 0.954527079 | NA | Bacteroidetes | Cytophagia | Cytophagales | Cytophagaceae | Hymenobacter |
| Otu00328 | 0.995968046 | -1.772229343 | 2.950630884 | -0.60062726 | 0.548088277 | NA | Bacteroidetes | Sphingobacteriia | Sphingobacteriales | Sphingobacteriaceae | Mucilaginibacter |
| Otu00367 | 0.206647921 | -0.096511972 | 2.967248342 | -0.032525748 | 0.974052783 | NA | Bacteroidetes | Sphingobacteriia | Sphingobacteriales | Sphingobacteriaceae | Mucilaginibacter |
| Otu00386 | 0.195817871 | -0.128444007 | 2.968128678 | -0.043274406 | 0.965482793 | NA | Bacteroidetes | Sphingobacteriia | Sphingobacteriales | Sphingobacteriaceae | Mucilaginibacter |
| Otu01140 | 0.050315129 | -1.019522235 | 2.971571138 | -0.343091983 | 0.731529262 | NA | Bacteroidetes | Sphingobacteriia | Sphingobacteriales | Sphingobacteriaceae | Pedobacter |
| Otu01376 | 0.260209656 | -1.053968596 | 2.971679105 | -0.354671066 | 0.722836029 | NA | Bacteroidetes | Flavobacteriia | Flavobacteriales | NS9_marine_group | unclassified |
| Otu00349 | 1.156827923 | -2.851971043 | 2.950118165 | -0.966731122 | 0.333678465 | NA | Bacteroidetes | Flavobacteriia | Flavobacteriales | Flavobacteriaceae | unclassified |
| Otu00066 | 0.055177619 | -1.37947632 | 2.972158905 | -0.464132762 | 0.642552631 | NA | Bacteroidetes | Flavobacteriia | Flavobacteriales | Flavobacteriaceae | Riemerella |
| Otu00620 | 0.344559719 | -1.779857969 | 2.960783642 | -0.601144219 | 0.547743934 | NA | Bacteroidetes | Flavobacteriia | Flavobacteriales | Flavobacteriaceae | Flavobacterium |
| Otu01067 | 0.101761907 | -0.471044216 | 2.972551876 | -0.158464591 | 0.874090721 | NA | Bacteroidetes | Flavobacteriia | Flavobacteriales | Flavobacteriaceae | Arenibacter |
| Otu00047 | 0.834284385 | 0.010944488 | 2.491644529 | 0.004392476 | 0.996495323 | NA | Bacteroidetes | Flavobacteriia | Flavobacteriales | Flavobacteriaceae | Flavobacterium |
| Otu00640 | 0.025545574 | -0.515422898 | 2.973561882 | -0.173335185 | 0.862387969 | NA | Bacteroidetes | Flavobacteriia | Flavobacteriales | Flavobacteriaceae | Flavobacterium |
| Otu00380 | 0.425391447 | 0.017998215 | 2.959224491 | 0.006082071 | 0.995147239 | NA | Bacteroidetes | Flavobacteriia | Flavobacteriales | Flavobacteriaceae | Flavobacterium |
| Otu00801 | 0.139116968 | -1.320531554 | 2.969010984 | -0.444771529 | 0.656484872 | NA | Bacteroidetes | Flavobacteriia | Flavobacteriales | Flavobacteriaceae | Ornithobacterium |
| Otu00844 | 0.475024348 | -1.236333967 | 2.966586819 | -0.416753003 | 0.676859081 | NA | Bacteroidetes | Sphingobacteriia | Sphingobacteriales | Saprospiraceae | unclassified |
| Otu00506 | 0.373091022 | -0.633350557 | 2.966709196 | -0.213485891 | 0.830947992 | NA | Bacteroidetes | Sphingobacteriia | Sphingobacteriales | Chitinophagaceae | Taibaiella |

| | | | | | | | | | | | | |
|---|---|---|---|---|---|---|---|---|---|---|---|---|
| Otu00429 | 0.229102008 | 0.74034805 | 2.963229393 | 0.249845001 | 0.802707217 | NA | Bacteroidetes | Sphingobacteriia | Sphingobacteriales | Chitinophagaceae | Chitinophaga |
| Otu00967 | 0.062690355 | -0.362495975 | 2.971497589 | -0.121991004 | 0.902906143 | NA | Bacteroidetes | Sphingobacteriia | Sphingobacteriales | Chitinophagaceae | Parafilimonas |
| Otu00304 | 0.692766434 | -0.336160536 | 1.534852413 | -0.21901815 | 0.82663591 | NA | Bacteroidetes | Sphingobacteriia | Sphingobacteriales | Chitinophagaceae | unclassified |
| Otu00880 | 0.077998512 | -0.839066299 | 2.972439974 | -0.282281999 | 0.777727287 | NA | Bacteroidetes | Sphingobacteriia | Sphingobacteriales | Chitinophagaceae | unclassified |
| Otu01080 | 0.069758629 | -0.974404078 | 2.971671678 | -0.327897622 | 0.742989071 | NA | Bacteroidetes | Sphingobacteriia | Sphingobacteriales | Chitinophagaceae | Flavisolibacter |
| Otu00579 | 0.061425246 | -0.883624807 | 2.970416926 | -0.297475011 | 0.766103883 | NA | Bacteroidetes | Sphingobacteriia | Sphingobacteriales | Chitinophagaceae | unclassified |
| Otu00282 | 0.254724054 | -0.658445606 | 2.965206173 | -0.222057276 | 0.824269296 | NA | Bacteroidetes | Sphingobacteriia | Sphingobacteriales | Chitinophagaceae | unclassified |
| Otu00427 | 0.221100738 | 0.953690379 | 2.96079612 | 0.322106062 | 0.747372346 | NA | Bacteroidetes | Sphingobacteriia | Sphingobacteriales | Chitinophagaceae | unclassified |
| Otu00930 | 0.170974819 | -0.479898135 | 2.970541274 | -0.161552421 | 0.871658327 | NA | Bacteroidetes | Sphingobacteriia | Sphingobacteriales | Chitinophagaceae | Ferruginibacter |
| Otu00489 | 0.231762287 | -0.814791972 | 2.952666098 | -0.275951274 | 0.782585491 | NA | Bacteroidetes | Sphingobacteriia | Sphingobacteriales | Chitinophagaceae | Segetibacter |
| Otu01243 | 0.070684709 | -1.601066042 | 2.968355393 | -0.539378151 | 0.589625954 | NA | Bacteroidetes | Sphingobacteriia | Sphingobacteriales | Chitinophagaceae | Taibaiella |
| Otu01036 | 0.10103901 | -0.541194657 | 2.971032266 | -0.182157112 | 0.85545943 | NA | Bacteroidetes | Sphingobacteriia | Sphingobacteriales | env.OPS_17 | unclassified |
| Otu00033 | 0.83586902 | -2.896178152 | 2.958285269 | -0.979005704 | 0.327577161 | NA | Bacteroidetes | Bacteroidia | Bacteroidales | Porphyromonadaceae | Macellibacteroides |
| Otu01043 | 0.313573611 | -0.964796119 | 2.969975065 | -0.324849905 | 0.745294672 | NA | Bacteroidetes | Bacteroidia | Bacteroidales | Prevotellaceae | Prevotella_9 |
| Otu00011 | 0.231890811 | -0.8096436 | 2.964098504 | -0.273150031 | 0.784737889 | NA | Bacteroidetes | Bacteroidia | Bacteroidales | Bacteroidaceae | Bacteroides |
| Otu01335 | 0.238263388 | -0.618960056 | 2.972779182 | -0.208209227 | 0.835065604 | NA | Bacteroidetes | Cytophagia | Cytophagales | Cytophagaceae | unclassified |
| Otu01101 | 0.03793837 | -1.509514871 | 2.969367677 | -0.508362398 | 0.611199218 | NA | Bacteroidetes | Cytophagia | Cytophagales | Cytophagaceae | Chryseolinea |
| Otu01064 | 0.060006504 | -0.818201041 | 2.973675975 | -0.275148015 | 0.783202524 | NA | Bacteroidetes | Cytophagia | Cytophagales | Cytophagaceae | Ohtaekwangia |
| Otu00501 | 0.191791537 | 0.273457542 | 2.955741556 | 0.092517406 | 0.926286962 | NA | Bacteroidetes | Cytophagia | Cytophagales | Cytophagaceae | unclassified |
| Otu00889 | 0.241028952 | -1.531271401 | 2.968570316 | -0.515827903 | 0.605974612 | NA | Bacteroidetes | Cytophagia | Cytophagales | Cytophagaceae | unclassified |
| Otu00697 | 0.255215749 | -0.109584428 | 2.969255437 | -0.036906366 | 0.970559664 | NA | Bacteroidetes | Cytophagia | Cytophagales | Cytophagaceae | unclassified |
| Otu01169 | 0.016812502 | -0.883768791 | 2.973554236 | -0.297209575 | 0.766306512 | NA | Bacteroidetes | Cytophagia | Cytophagales | Cytophagaceae | Adhaeribacter |
| Otu00258 | 0.080349134 | 0.098335546 | 2.965625763 | 0.033158447 | 0.973548234 | NA | Bacteroidetes | Cytophagia | Order_II | Rhodothermaceae | Rubrivirga |
