# Supplementary material for: Glucocorticoids modulate gastrointestinal microbiome in a wild bird
Source: R Soc Open Sci. 2018 Apr 18;5(4):171743. doi: 10.1098/rsos.171743 (PMC5936907; doi:10.1098/rsos.171743)
Supplement: Table S2 [file rsos171743supp2.pdf]

Table S2. Summary of DESeq2 analyses for the effect of sex on differential OTUs abundance. Differential OTU abundances were assessed using the Wald tests and p-vales adjusted by false discovery rate.
OTUs with a p-adj<0.05 are shown in bold.

OTU	baseMean	log2FoldChange	lfcSE	stat	pvalue	padj	Phylum	Class	Order	Family	Genus
**Otu00046**	**196.9431252**	**28.88425161**	**2.974390802**	**9.710980678**	**2.71E-22**	**1.06E-19**	**Firmicutes**	**Clostridia**	**Clostridiales**	**Lachnospiraceae**	**Tyzzerella_3**
**Otu00001**	**7711.896819**	**3.082781396**	**0.706633679**	**4.362630154**	**1.29E-05**	**0.002512333**	**Firmicutes**	**Bacilli**	**Lactobacillales**	**Enterococcaceae**	**Catellicoccus**
**Otu00013**	**58.65671401**	**8.208879467**	**2.17447428**	**3.775109939**	**0.000159937**	**0.020845137**	**Proteobacteria**	**Gammaproteobacteria**	**Enterobacteriales**	**Enterobacteriaceae**	**Proteus**
Otu00036	253.338566	9.000617076	2.735618755	3.290157687	0.001001313	0.097878299	Firmicutes	Clostridia	Clostridiales	Clostridiaceae_1	Candidatus_Arthromitus
Otu00043	184.7994206	5.713143105	1.86028469	3.071112253	0.00213263	0.166771633	Tenericutes	Mollicutes	Mycoplasmatales	Mycoplasmataceae	Candidatus_Bacilloplasma
Otu00018	408.5234806	3.573246056	1.298405125	2.752027074	0.005922762	0.385966684	Firmicutes	Erysipelotrichia	Erysipelotrichales	Erysipelotrichaceae	unclassified
Otu00072	29.0026344	4.482379851	1.886216727	2.37638644	0.017483142	0.615633094	Firmicutes	Clostridia	Clostridiales	Peptostreptococcaceae	Peptostreptococcus
Otu00010	1166.184605	3.270883275	1.399446475	2.337269294	0.019425186	0.615633094	Proteobacteria	Epsilonproteobacteria	Campylobacterales	Campylobacteraceae	Campylobacter
Otu00008	51.03832308	-4.583552093	1.934978067	-2.368787622	0.017846498	0.615633094	Proteobacteria	Epsilonproteobacteria	Campylobacterales	Campylobacteraceae	Campylobacter
Otu00005	1306.372893	2.694768677	1.080565599	2.493850145	0.012636588	0.615633094	Proteobacteria	Epsilonproteobacteria	Campylobacterales	Helicobacteraceae	Helicobacter
Otu00159	3.661596775	4.47778248	1.932039797	2.317645054	0.020468619	0.615633094	Firmicutes	Bacilli	Bacillales	unclassified	unclassified
Otu00074	25.73978137	6.970903779	2.938101529	2.372587778	0.017663965	0.615633094	Bacteroidetes	Sphingobacteriia	Sphingobacteriales	Sphingobacteriaceae	unclassified
Otu00092	14.09880287	6.849284544	2.947981779	2.323380895	0.0201587	0.615633094	Bacteroidetes	Flavobacteriia	Flavobacteriales	Flavobacteriaceae	unclassified
Otu00053	3.446043179	4.675910264	2.055968817	2.274309915	0.022947359	0.63712257	Actinobacteria	Actinobacteria	Micrococcales	Intrasporangiaceae	unclassified
Otu00071	59.30776908	3.970446485	1.76455759	2.250108757	0.024442042	0.63712257	Proteobacteria	Alphaproteobacteria	Rhizobiales	Aurantimonadaceae	unclassified
Otu00155	7.322988817	3.537024764	1.660421682	2.130196685	0.033155379	0.810234565	Firmicutes	Clostridia	Clostridiales	unclassified	unclassified
Otu00864	0.455713904	1.525182847	2.978025372	0.512145686	0.608549052	0.982761442	Actinobacteria	Thermoleophilia	Solirubrobacterales	Solirubrobacteraceae	Solirubrobacter
Otu00133	5.880239963	1.026669091	1.636062833	0.627524243	0.53031565	0.982761442	Actinobacteria	Thermoleophilia	Solirubrobacterales	Solirubrobacteraceae	Solirubrobacter
Otu00189	2.511716869	2.543559803	1.596756948	1.592953646	0.11117059	0.982761442	Actinobacteria	Thermoleophilia	Solirubrobacterales	unclassified	unclassified
Otu00284	0.588768823	1.188637587	2.113425169	0.562422367	0.573828283	0.982761442	Actinobacteria	Thermoleophilia	Solirubrobacterales	Solirubrobacteraceae	Solirubrobacter
Otu00335	0.994265292	2.598695247	2.810463057	0.924650207	0.355147888	0.982761442	Actinobacteria	Thermoleophilia	Solirubrobacterales	288-2	unclassified
Otu00253	0.730691921	0.733393398	2.959878888	0.247778178	0.80430604	0.982761442	Actinobacteria	Thermoleophilia	Solirubrobacterales	288-2	unclassified
Otu00522	0.719408596	1.946515679	2.969353449	0.655535191	0.512123237	0.982761442	Actinobacteria	Thermoleophilia	Solirubrobacterales	S1-80	unclassified
Otu00254	1.413375643	1.956549632	2.540370196	0.770182879	0.441191418	0.982761442	Actinobacteria	Thermoleophilia	Solirubrobacterales	480-2	unclassified
Otu00736	0.443300453	1.884363898	2.974475698	0.633511277	0.526399819	0.982761442	Actinobacteria	Thermoleophilia	Solirubrobacterales	480-2	unclassified
Otu00456	0.357434969	0.995709964	2.972675058	0.334954189	0.737659644	0.982761442	Actinobacteria	Thermoleophilia	Solirubrobacterales	480-2	unclassified
Otu00363	1.722349105	2.285597857	2.960535608	0.772021742	0.44010155	0.982761442	Actinobacteria	Thermoleophilia	Solirubrobacterales	480-2	unclassified
Otu00122	1.168713304	1.650988426	2.959448683	0.557870267	0.576932976	0.982761442	Actinobacteria	Thermoleophilia	Solirubrobacterales	Elev-16S-1332	unclassified
Otu00240	1.338761775	1.471934148	1.717684578	0.856929245	0.391483989	0.982761442	Actinobacteria	Thermoleophilia	Solirubrobacterales	480-2	unclassified
Otu00395	0.906434667	-0.921574394	2.957057849	-0.311652474	0.75530465	0.982761442	Actinobacteria	Thermoleophilia	Solirubrobacterales	Patulibacteraceae	Patulibacter
Otu00557	0.400337831	2.579067909	2.977874773	0.866076684	0.38644811	0.982761442	Actinobacteria	Thermoleophilia	Solirubrobacterales	unclassified	unclassified
Otu00337	1.130255182	2.875994144	2.854770056	1.007434605	0.313725969	0.982761442	Actinobacteria	Thermoleophilia	Solirubrobacterales	unclassified	unclassified
Otu00665	0.532129401	2.30134457	2.976432324	0.773188946	0.439410567	0.982761442	Actinobacteria	Thermoleophilia	Solirubrobacterales	Patulibacteraceae	Patulibacter
Otu00279	0.788465739	-1.364943706	2.955385949	-0.461849562	0.644189206	0.982761442	Actinobacteria	Thermoleophilia	Solirubrobacterales	unclassified	unclassified
Otu00746	0.474230132	1.423921773	2.976191029	0.478437627	0.632338758	0.982761442	Actinobacteria	Thermoleophilia	Gaiellales	unclassified	unclassified
Otu00375	0.448391117	-1.838900853	2.964249497	-0.620359674	0.535021015	0.982761442	Actinobacteria	Thermoleophilia	Gaiellales	Gaiellaceae	Gaiella
Otu00350	2.260551668	2.55956322	2.940563973	0.870432762	0.384063949	0.982761442	Actinobacteria	Thermoleophilia	Gaiellales	Gaiellaceae	Gaiella
Otu00219	0.439593923	1.553422862	2.982089754	0.520917541	0.602424215	0.982761442	Actinobacteria	Thermoleophilia	Gaiellales	Gaiellaceae	Gaiella
Otu00626	0.493493299	1.340767046	2.974863922	0.450698614	0.652206781	0.982761442	Actinobacteria	Rubrobacteria	Rubrobacterales	Rubrobacteriaceae	Rubrobacter
Otu00147	5.968259234	2.004758892	1.650401445	1.21470985	0.224476774	0.982761442	Actinobacteria	Actinobacteria	Frankiales	Nakamurellaceae	Nakamurella
Otu00265	0.575060325	2.043365651	2.967205476	0.688649865	0.491043633	0.982761442	Actinobacteria	Actinobacteria	Micromonosporales	Micromonosporaceae	Actinoplanes
Otu00150	3.639395038	0.621560303	1.493813376	0.416089662	0.677344394	0.982761442	Actinobacteria	Actinobacteria	Micromonosporales	Micromonosporaceae	Actinoplanes
Otu00232	0.949424147	1.551178787	2.957873994	0.524423552	0.599983979	0.982761442	Actinobacteria	Actinobacteria	Micromonosporales	Micromonosporaceae	Virgisporangium
Otu00124	3.329059964	0.65328743	1.416236115	0.461284261	0.644594675	0.982761442	Actinobacteria	Actinobacteria	Micromonosporales	Micromonosporaceae	unclassified
Otu00346	0.842903713	-2.152097806	2.961580617	-0.726672032	0.46742689	0.982761442	Actinobacteria	Actinobacteria	Micromonosporales	Micromonosporaceae	unclassified
Otu00193	2.985930733	-1.625427071	2.352814659	-0.690843652	0.4896638	0.982761442	Actinobacteria	Actinobacteria	Micromonosporales	Micromonosporaceae	unclassified
Otu00062	24.79909815	2.057967057	1.284728838	1.601868811	0.109184623	0.982761442	Actinobacteria	Actinobacteria	Micromonosporales	Micromonosporaceae	unclassified
Otu00466	0.480444781	-1.668799306	2.965021292	-0.562828776	0.573551483	0.982761442	Actinobacteria	Actinobacteria	Micromonosporales	Micromonosporaceae	unclassified
Otu00316	0.960193859	1.101848129	2.962750389	0.371900425	0.709966989	0.982761442	Actinobacteria	Actinobacteria	Pseudonocardiales	Pseudonocardiaceae	unclassified
Otu00586	1.070115383	3.055742481	2.969087697	1.02918566	0.30339244	0.982761442	Actinobacteria	Actinobacteria	Pseudonocardiales	Pseudonocardiaceae	Actinophytocola
Otu00088	5.915171257	-0.582441748	1.364485605	-0.426858111	0.669482678	0.982761442	Actinobacteria	Actinobacteria	Pseudonocardiales	Pseudonocardiaceae	Pseudonocardia
Otu00671	0.684691266	1.961881929	2.970418215	0.660473303	0.508950145	0.982761442	Actinobacteria	Actinobacteria	Pseudonocardiales	Pseudonocardiaceae	Pseudonocardia
Otu00162	1.949040728	0.549696498	1.694262647	0.324445858	0.745600506	0.982761442	Actinobacteria	Actinobacteria	Pseudonocardiales	Pseudonocardiaceae	Actinomycetospora
Otu00377	0.320287168	0.825665233	2.97223356	0.27779285	0.78117138	0.982761442	Actinobacteria	Actinobacteria	Streptomycetales	Streptomycetaceae	Streptomyces
Otu00440	0.748773821	1.662250548	2.969239443	0.559823679	0.575599711	0.982761442	Actinobacteria	Actinobacteria	Streptomycetales	Streptomycetaceae	unclassified
Otu00089	1.484055471	-0.547281821	1.907383008	-0.28692812	0.774167358	0.982761442	Actinobacteria	Actinobacteria	unclassified	unclassified	unclassified
Otu00530	0.499539852	1.693070327	2.976432221	0.568825426	0.569474618	0.982761442	Actinobacteria	Actinobacteria	Micrococcales	Brevibacteriaceae	Brevibacterium
Otu00104	12.3255541	0.87456155	1.651379822	0.529594427	0.596393159	0.982761442	Actinobacteria	Actinobacteria	Micrococcales	Micrococcaceae	unclassified
Otu00483	0.457266542	2.291834925	2.978103482	0.769561883	0.441559823	0.982761442	Actinobacteria	Actinobacteria	Actinomycetales	Actinomycetaceae	Actinomyces
Otu00333	0.321019617	1.330981017	2.974230529	0.447504322	0.654510968	0.982761442	Actinobacteria	Actinobacteria	Micrococcales	Dermabacteraceae	unclassified
Otu00134	7.685948797	1.287439736	1.655140163	0.77784333	0.43666138	0.982761442	Actinobacteria	Actinobacteria	Actinomycetales	Actinomycetaceae	Varibaculum
Otu00307	1.653809182	1.88213773	2.406142253	0.782222135	0.434084038	0.982761442	Actinobacteria	Actinobacteria	Actinomycetales	Actinomycetaceae	Actinomyces
Otu00067	0.500097622	0.88001492	2.976739749	0.295630453	0.767512315	0.982761442	Actinobacteria	Actinobacteria	Micrococcales	unclassified	unclassified
Otu00154	4.63727302	0.439533955	1.690051537	0.260071333	0.79480875	0.982761442	Actinobacteria	Actinobacteria	Micrococcales	Micrococcaceae	unclassified
Otu00093	9.442991464	-0.498590327	1.557986833	-0.32002217	0.748951525	0.982761442	Actinobacteria	Actinobacteria	Micrococcales	Micrococcaceae	Arthrobacter
Otu00298	2.20634243	2.701201444	2.914671142	0.92676028	0.354051012	0.982761442	Actinobacteria	Actinobacteria	Micrococcales	Micrococcaceae	Arthrobacter
Otu00217	1.232096378	-1.55711117	2.95143711	-0.527577283	0.597792764	0.982761442	Actinobacteria	Actinobacteria	Micrococcales	Micrococcaceae	Arthrobacter
Otu00097	11.39696283	2.489261922	1.362080181	1.827544337	0.067617966	0.982761442	Actinobacteria	Actinobacteria	Micrococcales	Cellulomonadaceae	Cellulomonas
Otu00303	0.868891138	1.348459942	2.964103583	0.454930101	0.649159527	0.982761442	Actinobacteria	Actinobacteria	Micrococcales	Promicromonosporaceae	Cellulosimicrobium
Otu00611	0.417596788	2.319296686	2.977811346	0.77885951	0.436062478	0.982761442	Actinobacteria	Actinobacteria	Micrococcales	unclassified	unclassified
Otu00140	3.823014991	2.915517118	1.743116863	1.672588441	0.094408344	0.982761442	Actinobacteria	Actinobacteria	Micrococcales	Microbacteriaceae	Curtobacterium
Otu00084	12.75940146	1.103855801	1.137957078	0.970032896	0.332030095	0.982761442	Actinobacteria	Actinobacteria	Micrococcales	Microbacteriaceae	unclassified
Otu00177	2.344313097	1.108863501	1.379911135	0.803576022	0.421641875	0.982761442	Actinobacteria	Actinobacteria	Micrococcales	Microbacteriaceae	unclassified
Otu00267	0.727431328	0.960555922	2.957571482	0.324778599	0.745348643	0.982761442	Actinobacteria	Actinobacteria	Micrococcales	Intrasporangiaceae	unclassified
Otu00223	1.886934395	-0.587860943	2.267964518	-0.259202002	0.795479387	0.982761442	Actinobacteria	Actinobacteria	Micrococcales	Intrasporangiaceae	Janibacter
Otu00401	0.678715921	2.143767569	2.970975699	0.721570213	0.470558758	0.982761442	Actinobacteria	Actinobacteria	Micrococcales	Intrasporangiaceae	unclassified
Otu00173	2.05679223	-2.253917034	2.106267918	-1.070099874	0.284574356	0.982761442	Actinobacteria	Actinobacteria	Kineosporiales	Kineosporiaceae	unclassified
Otu00738	0.794316944	2.665734432	2.972548299	0.896784228	0.369834065	0.982761442	Actinobacteria	Actinobacteria	Kineosporiales	Kineosporiaceae	unclassified
Otu00268	1.039906779	1.447045395	1.64981859	0.877093642	0.380435778	0.982761442	Actinobacteria	Actinobacteria	Frankiales	Geodermatophilaceae	Modestobacter
Otu00301	0.640563606	1.116198196	2.96403619	0.376580488	0.70648539	0.982761442	Actinobacteria	Actinobacteria	Frankiales	Geodermatophilaceae	Modestobacter
Otu00105	8.405437787	1.683420825	1.360189477	1.237637001	0.215850688	0.982761442	Actinobacteria	Actinobacteria	Frankiales	Geodermatophilaceae	Blastococcus
Otu00170	0.369922494	2.325054807	2.977057233	0.780990967	0.434807808	0.982761442	Actinobacteria	Actinobacteria	Corynebacteriales	Nocardiaceae	Gordonia
Otu00153	1.846319181	0.839339061	1.672460917	0.5018587	0.615766918	0.982761442	Actinobacteria	Actinobacteria	Corynebacteriales	Nocardiaceae	Williamsia
Otu00198	0.895808011	-1.921394539	2.933755911	-0.654926516	0.512515067	0.982761442	Actinobacteria	Actinobacteria	Corynebacteriales	Nocardiaceae	unclassified
Otu00183	7.840505283	1.2814284	2.419308146	0.529667294	0.596342628	0.982761442	Actinobacteria	Actinobacteria	Corynebacteriales	Nocardiaceae	Rhodococcus
Otu00184	1.666913948	1.490794775	2.496549734	0.59714203	0.550412559	0.982761442	Actinobacteria	Actinobacteria	Corynebacteriales	Nocardiaceae	Rhodococcus
Otu00227	0.768355861	2.587234823	2.970786012	0.870892354	0.383812932	0.982761442	Actinobacteria	Actinobacteria	Corynebacteriales	Mycobacteriaceae	Mycobacterium
Otu00059	14.35979803	2.129813877	1.179795028	1.805240594	0.071037041	0.982761442	Actinobacteria	Actinobacteria	Corynebacteriales	Mycobacteriaceae	Mycobacterium
Otu00366	1.316925492	2.555918101	2.46905428	1.035181009	0.300584386	0.982761442	Actinobacteria	Actinobacteria	Corynebacteriales	Mycobacteriaceae	Mycobacterium
Otu00422	0.686154693	1.622300465	2.96779422	0.546635091	0.584629456	0.982761442	Actinobacteria	Actinobacteria	Corynebacteriales	Corynebacteriaceae	Corynebacterium_1
Otu00012	282.3827	-2.786643753	1.540043188	-1.809458186	0.070379851	0.982761442	Actinobacteria	Actinobacteria	Corynebacteriales	Corynebacteriaceae	unclassified
Otu00195	0.894250777	2.401223771	2.966223245	0.809522269	0.418214799	0.982761442	Actinobacteria	Actinobacteria	Frankiales	Acidothermaceae	Acidothermus
Otu00423	0.547046117	1.081221775	2.964367969	0.364739393	0.71530595	0.982761442	Actinobacteria	Actinobacteria	unclassified	unclassified	unclassified
Otu00221	0.524187383	2.475831956	2.975652336	0.832029981	0.405392022	0.982761442	Actinobacteria	Actinobacteria	Frankiales	Acidothermaceae	Acidothermus
Otu00397	0.575887473	-1.083522176	2.963325577	-0.365643986	0.714630748	0.982761442	Actinobacteria	Actinobacteria	Frankiales	Acidothermaceae	Acidothermus
Otu00144	4.211222821	2.645576345	1.666012971	1.587968635	0.112293432	0.982761442	Actinobacteria	Actinobacteria	Propionibacteriales	Nocardioidaceae	Marmoricola
Otu00206	2.028827699	0.7251119	2.254330653	0.321652859	0.747715694	0.982761442	Actinobacteria	Actinobacteria	Propionibacteriales	Nocardioidaceae	Marmoricola
Otu00130	3.769428884	-1.132635556	1.984309145	-0.570795916	0.568137992	0.982761442	Actinobacteria	Actinobacteria	Propionibacteriales	Nocardioidaceae	Nocardioides
Otu00447	1.299279072	2.288513285	2.382165823	0.96068597	0.336710087	0.982761442	Actinobacteria	Actinobacteria	Propionibacteriales	Nocardioidaceae	Nocardioides
Otu00182	3.490886982	2.65437096	1.692022489	1.568756312	0.116704732	0.982761442	Actinobacteria	Actinobacteria	Propionibacteriales	Nocardioidaceae	Nocardioides
Otu00106	9.775271092	1.751473838	1.139282837	1.53734769	0.124208189	0.982761442	Actinobacteria	Actinobacteria	Propionibacteriales	Nocardioidaceae	Nocardioides
Otu00263	1.181834053	1.259110971	2.249327443	0.5597722	0.575634828	0.982761442	Actinobacteria	Actinobacteria	Propionibacteriales	Nocardioidaceae	Nocardioides
Otu00270	0.736572218	2.400908443	2.970977702	0.808120654	0.419021129	0.982761442	Actinobacteria	Actinobacteria	Propionibacteriales	Nocardioidaceae	Nocardioides
Otu00425	0.500944159	1.865437591	2.386460097	0.781675584	0.434405254	0.982761442	Actinobacteria	Actinobacteria	Propionibacteriales	Nocardioidaceae	Nocardioides
Otu00161	3.38538582	1.746705054	1.777684719	0.982573026	0.325817626	0.982761442	Actinobacteria	Actinobacteria	Propionibacteriales	Nocardioidaceae	Nocardioides
Otu00607	0.327050865	1.421185108	2.975350647	0.477652982	0.632897215	0.982761442	Actinobacteria	Actinobacteria	Propionibacteriales	Nocardioidaceae	unclassified
Otu00178	2.833792985	2.541275778	1.521957394	1.669741734	0.094970473	0.982761442	Actinobacteria	Actinobacteria	Propionibacteriales	Nocardioidaceae	Aeromicrobium
Otu00242	1.971839732	0.702170625	2.231624332	0.314645532	0.753030804	0.982761442	Actinobacteria	Actinobacteria	Frankiales	unclassified	unclassified
Otu00306	0.738435386	-0.882400576	2.955540801	-0.298558076	0.765277257	0.982761442	Actinobacteria	Actinobacteria	Frankiales	Frankiaceae	Jatrophihabitans
Otu00360	0.383933634	2.525144985	2.977572493	0.848054914	0.396407389	0.982761442	Actinobacteria	Actinobacteria	Frankiales	unclassified	unclassified
Otu00271	0.360181001	0.931266834	2.970847174	0.313468442	0.753924793	0.982761442	Actinobacteria	Actinobacteria	unclassified	unclassified	unclassified
Otu00719	0.458005444	1.883042216	2.974078197	0.633151548	0.526634682	0.982761442	Actinobacteria	Acidimicrobiia	Acidimicrobiales	Acidimicrobiaceae	unclassified

Otu00283	1.669490731	2.115354616	1.872512651	1.129687756	0.258607818	0.982761442	Actinobacteria	Acidimicrobiia	Acidimicrobiales	Acidimicrobiaceae	unclassified
Otu00811	0.69758725	2.567536165	2.97434479	0.863227483	0.388012406	0.982761442	Actinobacteria	unclassified	unclassified	unclassified	unclassified
Otu00540	0.395231743	1.196374115	2.972515156	0.402478727	0.687331741	0.982761442	Actinobacteria	Acidimicrobiia	Acidimicrobiales	Acidimicrobiales_Incertae_	Candidatus_Microthrix
Otu00344	1.447979801	1.869966879	2.960730874	0.631589617	0.527655073	0.982761442	Actinobacteria	Acidimicrobiia	Acidimicrobiales	Iamiaceae	Iamia
Otu00273	1.138390863	1.135077374	2.95804219	0.383725891	0.701181621	0.982761442	Actinobacteria	Acidimicrobiia	Acidimicrobiales	Iamiaceae	Iamia
Otu00419	0.406867928	0.977946824	2.971662098	0.329090856	0.742087015	0.982761442	Actinobacteria	Acidimicrobiia	Acidimicrobiales	Iamiaceae	Iamia
Otu00981	0.437197677	1.652445033	2.980464842	0.554425273	0.579287834	0.982761442	Actinobacteria	Acidimicrobiia	Acidimicrobiales	Iamiaceae	Iamia
Otu00503	0.759246335	2.220391684	2.969827192	0.747650129	0.454671219	0.982761442	Actinobacteria	Acidimicrobiia	Acidimicrobiales	Iamiaceae	Iamia
Otu00455	0.566960834	1.501137523	2.968511459	0.505686956	0.613076427	0.982761442	Actinobacteria	Acidimicrobiia	Acidimicrobiales	Acidimicrobiales_Incertae_	Candidatus_Microthrix
Otu00334	1.543968181	3.834573933	2.95445076	1.297897391	0.194322597	0.982761442	Actinobacteria	Acidimicrobiia	Acidimicrobiales	unclassified	unclassified
Otu00679	0.677742363	1.641280974	2.979788387	0.550804541	0.581767671	0.982761442	Actinobacteria	unclassified	unclassified	unclassified	unclassified
Otu00754	0.857528433	2.397271459	2.969773035	0.807223795	0.419537559	0.982761442	Actinobacteria	MB-A2-108	unclassified	unclassified	unclassified
Otu00129	18.62653038	3.449577968	2.216663561	1.556202768	0.119659883	0.982761442	Firmicutes	Clostridia	Clostridiales	Family_XI	Parvimonas
Otu00745	0.386115603	1.239480679	2.973219972	0.416881593	0.676765018	0.982761442	Firmicutes	Clostridia	Clostridiales	Family_XIII	[Eubacterium]_brachy_group
Otu00214	2.338051656	2.346546894	2.501575606	0.938027573	0.34823024	0.982761442	Firmicutes	Clostridia	Clostridiales	Peptostreptococcaceae	unclassified
Otu00014	222.2223755	2.846607306	1.514954161	1.87900557	0.060243733	0.982761442	Firmicutes	Clostridia	Clostridiales	Peptostreptococcaceae	Peptoclostridium
Otu00078	0.41793451	1.856281463	2.983908347	0.622097346	0.53387786	0.982761442	Firmicutes	Clostridia	Clostridiales	Peptostreptococcaceae	unclassified
Otu00039	12.00729958	-1.809340915	1.873774689	-0.965612849	0.334237944	0.982761442	Firmicutes	Clostridia	Clostridiales	Peptostreptococcaceae	Peptostreptococcus
Otu00032	4.636276263	-2.7878875	1.420238362	-1.962971551	0.04964948	0.982761442	Firmicutes	Clostridia	Clostridiales	Clostridiaceae_1	Clostridium_sensu_stricto_1
Otu00452	0.506879231	1.064652621	2.968188264	0.3586877	0.719828731	0.982761442	Proteobacteria	Gammaproteobacteria	Xanthomonadales	Xanthomonadaceae	Luteimonas
Otu00326	1.525236835	1.134980135	1.725794855	0.65765646	0.510758906	0.982761442	Proteobacteria	Gammaproteobacteria	Xanthomonadales	Xanthomonadaceae	unclassified
Otu00102	5.728157963	1.486652042	1.670245164	0.890080136	0.373422858	0.982761442	Proteobacteria	Gammaproteobacteria	Xanthomonadales	Xanthomonadaceae	Stenotrophomonas
Otu00058	267.8344798	-2.021569604	1.378598857	-1.466394371	0.142540879	0.982761442	Proteobacteria	Gammaproteobacteria	Pseudomonadales	Pseudomonadaceae	Pseudomonas
Otu00244	1.24912352	-2.245125314	2.958687018	-0.758824878	0.447957322	0.982761442	Proteobacteria	Gammaproteobacteria	Xanthomonadales	Xanthomonadales_Incerta	Acidibacter
Otu00107	0.402458032	2.15970704	2.974514164	0.726070518	0.467795541	0.982761442	Proteobacteria	Gammaproteobacteria	Legionellales	Coxiellaceae	Rickettsiella
Otu00015	131.2317671	-0.817201707	1.488526793	-0.549000334	0.58300522	0.982761442	Proteobacteria	Gammaproteobacteria	Enterobacteriales	Enterobacteriaceae	Edwardsiella
Otu00002	1645.206147	-1.193039387	0.741863086	-1.608166534	0.107798711	0.982761442	Proteobacteria	Gammaproteobacteria	Enterobacteriales	Enterobacteriaceae	unclassified
Otu00016	8.229295065	-4.205334694	2.236550945	-1.880276728	0.060070373	0.982761442	Proteobacteria	Gammaproteobacteria	Pasteurellales	Pasteurellaceae	unclassified
Otu00030	5.679230633	0.928402108	1.547241092	0.600037132	0.548481489	0.982761442	Proteobacteria	Gammaproteobacteria	Pasteurellales	Pasteurellaceae	Pasteurella
Otu00464	0.910289643	1.21480909	2.964363454	0.409804367	0.681949462	0.982761442	Proteobacteria	Gammaproteobacteria	unclassified	unclassified	unclassified
Otu00023	200.9623519	-0.735685131	1.448004545	-0.508068247	0.611405483	0.982761442	Proteobacteria	Gammaproteobacteria	Vibrionales	Vibrionaceae	Vibrio
Otu00025	67.02470005	1.225763667	1.414845291	0.866358799	0.386293431	0.982761442	Proteobacteria	Gammaproteobacteria	Enterobacteriales	Enterobacteriaceae	unclassified
Otu00564	0.329338218	0.739629222	2.975331996	0.248587123	0.80368017	0.982761442	Proteobacteria	Gammaproteobacteria	Pseudomonadales	Moraxellaceae	Moraxella
Otu00060	2.262380845	-1.834980559	2.953477929	-0.621294827	0.534405657	0.982761442	Proteobacteria	Gammaproteobacteria	Pseudomonadales	Moraxellaceae	Moraxella
Otu00055	6.520711474	-1.163821889	1.467934159	-0.792829761	0.427877014	0.982761442	Proteobacteria	Gammaproteobacteria	Pseudomonadales	Moraxellaceae	Psychrobacter
Otu00063	2.951050171	-3.538872891	2.954956108	-1.197605907	0.231070475	0.982761442	Proteobacteria	Gammaproteobacteria	Pseudomonadales	Moraxellaceae	Psychrobacter
Otu00325	0.81095526	2.813053651	2.970190084	0.947095496	0.34359012	0.982761442	Proteobacteria	Betaproteobacteria	Burkholderiales	Alcaligenaceae	unclassified
Otu00378	2.184572527	4.070348084	2.947773023	1.380821404	0.167333881	0.982761442	Proteobacteria	Betaproteobacteria	Nitrosomonadales	Nitrosomonadaceae	Nitrosospira
Otu00511	1.266322374	2.92091854	2.967515992	0.984297489	0.324969264	0.982761442	Proteobacteria	Betaproteobacteria	Burkholderiales	Burkholderiaceae	Lautropia
Otu00085	0.62404549	0.726111987	2.645919328	0.274427107	0.783756414	0.982761442	Proteobacteria	Betaproteobacteria	Burkholderiales	Alcaligenaceae	Sutterella
Otu00287	0.679490185	-1.191575091	2.107291111	-0.565453479	0.571765358	0.982761442	Proteobacteria	Betaproteobacteria	Burkholderiales	Comamonadaceae	unclassified
Otu00163	3.284861225	1.117136994	2.014693695	0.554494709	0.579240326	0.982761442	Proteobacteria	Betaproteobacteria	Burkholderiales	Comamonadaceae	Verminephrobacter
Otu00121	6.922869835	1.810003007	1.562242158	1.158593114	0.246622077	0.982761442	Proteobacteria	Betaproteobacteria	Burkholderiales	Comamonadaceae	unclassified
Otu00519	0.529976881	2.20741978	2.973868947	0.742272043	0.457922527	0.982761442	Proteobacteria	Betaproteobacteria	Burkholderiales	Oxalobacteraceae	Duganella
Otu00076	12.4791655	0.709446083	2.002454806	0.354288187	0.72312292	0.982761442	Proteobacteria	Gammaproteobacteria	Pseudomonadales	Moraxellaceae	Acinetobacter
Otu00083	9.29662804	2.956457359	1.59621612	1.852166082	0.064001978	0.982761442	Proteobacteria	Gammaproteobacteria	Pseudomonadales	Moraxellaceae	Acinetobacter
Otu00280	0.477987226	-1.221804201	2.966935732	-0.411806763	0.680481064	0.982761442	Proteobacteria	Deltaproteobacteria	Myxococcales	unclassified	unclassified
Otu00138	0.62504248	-1.827785137	2.9637626	-0.616711047	0.537425333	0.982761442	Proteobacteria	Epsilonproteobacteria	Campylobacterales	Campylobacteraceae	Campylobacter
Otu00299	0.795239149	0.787243038	2.964557428	0.265551624	0.790584547	0.982761442	Proteobacteria	Epsilonproteobacteria	Campylobacterales	Helicobacteraceae	Helicobacter
Otu00019	1.963523867	-2.730707607	2.957282615	-0.923384053	0.355807099	0.982761442	Proteobacteria	Epsilonproteobacteria	Campylobacterales	Helicobacteraceae	Helicobacter
Otu00275	0.532999076	-1.322535661	2.461542521	-0.537279226	0.591074753	0.982761442	Proteobacteria	Alphaproteobacteria	Rhodospirillales	Rhodospirillales_Incertae_	Reyranella
Otu00239	0.771388388	-1.61145017	2.3304721	-0.691469411	0.489270597	0.982761442	Proteobacteria	Alphaproteobacteria	Rhodospirillales	Rhodospirillales_Incertae_	Reyranella
Otu00231	0.461181614	-1.50399399	2.966052634	-0.507069218	0.612106252	0.982761442	Proteobacteria	Alphaproteobacteria	Rhodospirillales	Acetobacteraceae	Acidiphilium
Otu00642	0.879187847	2.078665506	2.96830516	0.700286997	0.483748091	0.982761442	Proteobacteria	Alphaproteobacteria	Rhodospirillales	Acetobacteraceae	Roseomonas
Otu00290	1.4808442	1.178492829	2.950847943	0.399374299	0.689617428	0.982761442	Proteobacteria	Alphaproteobacteria	Rhodospirillales	Acetobacteraceae	Roseomonas
Otu00252	2.484997387	1.690327742	2.659161211	0.635662003	0.524996747	0.982761442	Proteobacteria	Alphaproteobacteria	Rhodospirillales	Rhodospirillaceae	Skermanella
Otu00320	0.446980993	-1.119310006	2.96788313	-0.377140863	0.706068925	0.982761442	Proteobacteria	Alphaproteobacteria	Rhodospirillales	Rhodospirillaceae	Skermanella
Otu00403	0.492146492	-1.973546582	2.963424066	-0.665968332	0.505431342	0.982761442	Proteobacteria	Alphaproteobacteria	Rhodospirillales	Rhodospirillales_Incertae_	Candidatus_Alysiosphaera
Otu00451	1.734320034	2.244272144	2.959270883	0.758386857	0.448219424	0.982761442	Proteobacteria	Alphaproteobacteria	Rhodospirillales	Rhodospirillales_Incertae_	Candidatus_Alysiosphaera
Otu00179	3.810222184	2.037077644	2.086611428	0.976261137	0.328935074	0.982761442	Proteobacteria	Alphaproteobacteria	Caulobacterales	Caulobacteraceae	Brevundimonas
Otu00759	0.583039988	2.583678748	2.976784382	0.867942859	0.385425614	0.982761442	Proteobacteria	Alphaproteobacteria	Rhizobiales	Hyphomicrobiaceae	Pedomicrobium
Otu00672	0.317571402	2.306134616	2.980910438	0.773634319	0.439147071	0.982761442	Proteobacteria	Alphaproteobacteria	Rhizobiales	Hyphomicrobiaceae	Hyphomicrobium
Otu00747	0.326014681	1.04936162	2.978536643	0.352307776	0.724607457	0.982761442	Proteobacteria	Alphaproteobacteria	Rhizobiales	unclassified	unclassified
Otu00771	0.438742277	2.071714506	2.975709734	0.696208532	0.486298245	0.982761442	Proteobacteria	Alphaproteobacteria	Rhizobiales	Rhodobiaceae	unclassified
Otu00385	1.101910744	3.482126629	2.960371661	1.176246441	0.239496412	0.982761442	Proteobacteria	Alphaproteobacteria	Rhizobiales	Rhizobiaceae	Kaistia
Otu00095	8.587591049	0.438972679	1.675909424	0.261931029	0.793374617	0.982761442	Proteobacteria	Alphaproteobacteria	Rhizobiales	Hyphomicrobiaceae	Devosia
Otu00201	1.479043734	1.598836555	1.501490256	1.064833121	0.286951454	0.982761442	Proteobacteria	Alphaproteobacteria	Rhizobiales	Hyphomicrobiaceae	Devosia
Otu00327	0.509079216	-0.752030025	2.868517201	-0.262166817	0.793192835	0.982761442	Proteobacteria	Alphaproteobacteria	Rhizobiales	Hyphomicrobiaceae	unclassified
Otu00295	1.042138784	2.198498915	2.116824201	1.038583608	0.298998433	0.982761442	Proteobacteria	Alphaproteobacteria	Rhizobiales	Hyphomicrobiaceae	Devosia
Otu00100	2.340740749	-0.774387459	1.775453852	-0.436163102	0.662718397	0.982761442	Proteobacteria	Alphaproteobacteria	Rhizobiales	Phyllobacteriaceae	unclassified
Otu01018	0.436450737	1.658168959	2.980555408	0.556328849	0.577986069	0.982761442	Proteobacteria	Alphaproteobacteria	Rhizobiales	unclassified	unclassified
Otu01239	0.348793625	1.856281529	2.983908349	0.622097368	0.533877846	0.982761442	Proteobacteria	Alphaproteobacteria	Rhizobiales	D05-2	unclassified
Otu01021	0.326148423	1.613854002	2.984648599	0.540718261	0.58870179	0.982761442	Proteobacteria	Alphaproteobacteria	Rhizobiales	alphaI_cluster	unclassified
Otu00654	0.373829147	1.099995966	2.975624729	0.369668915	0.711629195	0.982761442	Proteobacteria	Alphaproteobacteria	Rhizobiales	Xanthobacteraceae	Labrys
Otu00042	0.860211736	-0.778112201	2.296788462	-0.338782702	0.734773433	0.982761442	Proteobacteria	Alphaproteobacteria	Rhizobiales	Methylobacteriaceae	Methylobacterium
Otu00139	5.24282447	-0.513153884	1.626412617	-0.315512729	0.752372388	0.982761442	Proteobacteria	Alphaproteobacteria	Rhizobiales	Methylobacteriaceae	Microvirga
Otu00215	1.05431967	1.359170963	2.056406088	0.660944826	0.508647698	0.982761442	Proteobacteria	Alphaproteobacteria	Rhizobiales	Methylobacteriaceae	Microvirga
Otu00099	9.254444548	0.768152382	1.689123599	0.454763868	0.649279127	0.982761442	Proteobacteria	Alphaproteobacteria	Rhizobiales	Methylobacteriaceae	Microvirga
Otu00090	6.827741334	-0.883311657	1.652016644	-0.534686899	0.592866382	0.982761442	Proteobacteria	Alphaproteobacteria	Rhizobiales	Methylobacteriaceae	unclassified
Otu00208	2.127401634	1.132292258	2.06215252	0.549082692	0.582948702	0.982761442	Proteobacteria	Alphaproteobacteria	Rhizobiales	unclassified	unclassified
Otu00315	0.330573906	2.824577599	2.977415901	0.948667466	0.342789766	0.982761442	Proteobacteria	Alphaproteobacteria	Rhizobiales	Beijerinckiaceae	Chelatococcus
Otu00296	2.399588329	3.495736721	2.511349434	1.391975435	0.163929837	0.982761442	Proteobacteria	Alphaproteobacteria	Rhizobiales	Methylobacteriaceae	Methylobacterium
Otu00079	8.97091848	1.35973103	1.259613633	1.079482624	0.280372636	0.982761442	Proteobacteria	Alphaproteobacteria	Rhizobiales	Methylobacteriaceae	Methylobacterium
Otu00187	3.368174125	-1.75590257	2.634948643	-0.666389675	0.50516206	0.982761442	Proteobacteria	Alphaproteobacteria	Rhizobiales	Methylobacteriaceae	Methylobacterium
Otu00330	0.638359954	3.107846107	2.972135075	1.045661125	0.295717517	0.982761442	Proteobacteria	Alphaproteobacteria	Rhizobiales	Methylobacteriaceae	Methylobacterium
Otu00186	1.940720046	1.193489265	1.83403553	0.650744899	0.515211175	0.982761442	Proteobacteria	Alphaproteobacteria	Rhizobiales	Bradyrhizobiaceae	Bosea
Otu00482	0.351254849	2.254760645	2.976594191	0.757496824	0.448752268	0.982761442	Proteobacteria	Alphaproteobacteria	Rhizobiales	unclassified	unclassified
Otu00319	1.535985119	1.501101573	2.956691714	0.507696344	0.611666313	0.982761442	Proteobacteria	Alphaproteobacteria	Rhizobiales	Rhizobiaceae	unclassified
Otu00034	30.89457494	1.364555379	1.145475076	1.191257154	0.233552649	0.982761442	Proteobacteria	Alphaproteobacteria	Rhizobiales	unclassified	unclassified
Otu00136	6.228095345	1.937388751	1.45793337	1.328859598	0.183894295	0.982761442	Proteobacteria	Alphaproteobacteria	Rhizobiales	Aurantimonadaceae	unclassified
Otu00235	4.207574809	3.259090551	1.840633682	1.770635072	0.076621406	0.982761442	Proteobacteria	Alphaproteobacteria	Rhizobiales	unclassified	unclassified
Otu00229	1.016970848	-1.089058231	1.58362394	-0.68770003	0.491641701	0.982761442	Proteobacteria	Alphaproteobacteria	unclassified	unclassified	unclassified
Otu00050	0.938457879	3.334235609	2.335398886	1.427694271	0.153379876	0.982761442	Proteobacteria	Alphaproteobacteria	Rhizobiales	Phyllobacteriaceae	Mesorhizobium
Otu00098	2.27469717	-0.44284623	1.570829094	-0.281918785	0.778005785	0.982761442	Proteobacteria	Alphaproteobacteria	Rhizobiales	Xanthobacteraceae	Variibacter
Otu00123	1.742650996	1.655105817	1.614021703	1.025454499	0.305148785	0.982761442	Proteobacteria	Alphaproteobacteria	Rhizobiales	Xanthobacteraceae	Pseudolabrys
Otu00336	0.949970046	1.855493156	2.782579561	0.666824835	0.504884027	0.982761442	Proteobacteria	Alphaproteobacteria	Rhizobiales	unclassified	unclassified
Otu00224	1.028606566	1.189389195	2.957518545	0.402157815	0.687567886	0.982761442	Proteobacteria	Alphaproteobacteria	Rhizobiales	unclassified	unclassified
Otu00158	4.724354716	0.873092064	2.185111404	0.399564097	0.689477605	0.982761442	Proteobacteria	Alphaproteobacteria	Rhizobiales	Bradyrhizobiaceae	unclassified
Otu00241	0.814124485	1.671836625	2.769525993	0.603654427	0.546073419	0.982761442	Proteobacteria	Alphaproteobacteria	Rhizobiales	Bradyrhizobiaceae	Tardiphaga
Otu00686	0.66393422	2.039049075	2.972362004	0.68600294	0.492711254	0.982761442	Proteobacteria	Alphaproteobacteria	Rhizobiales	JG34-KF-361	unclassified
Otu00481	0.558165284	1.477028467	2.969821052	0.497345948	0.618945115	0.982761442	Proteobacteria	Alphaproteobacteria	Rhizobiales	JG34-KF-361	unclassified
Otu00020	66.76096931	3.027814781	1.516521286	1.996552774	0.045873787	0.982761442	Proteobacteria	Alphaproteobacteria	Rhodobacterales	Rhodobacteraceae	Paracoccus
Otu00216	1.151804919	-0.920154942	2.556496584	-0.359928094	0.718900907	0.982761442	Proteobacteria	Alphaproteobacteria	Rhodobacterales	Rhodobacteraceae	unclassified
Otu00247	1.857864702	1.65134854	1.36608008	1.208822648	0.226730989	0.982761442	Proteobacteria	Alphaproteobacteria	Rhodobacterales	Rhodobacteraceae	unclassified
Otu00379	2.287288421	2.757111199	2.960173629	0.931401852	0.351645733	0.982761442	Proteobacteria	Alphaproteobacteria	Rhodobacterales	Rhodobacteraceae	Rubellimicrobium
Otu00251	0.660404448	-1.451819529	2.958374317	-0.490749098	0.623603916	0.982761442	Proteobacteria	Alphaproteobacteria	Rhodobacterales	Rhodobacteraceae	Sedimentitalea
Otu00472	0.936357804	1.935414099	2.965479723	0.652647895	0.513983301	0.982761442	Proteobacteria	Alphaproteobacteria	Rhodobacterales	Rhodobacteraceae	unclassified
Otu00352	0.719853647	0.919395216	2.963884256	0.310199433	0.756409302	0.982761442	Proteobacteria	Alphaproteobacteria	Rhodobacterales	Rhodobacteraceae	unclassified
Otu00278	1.923786512	3.466120272	2.963829692	1.169473496	0.242212913	0.982761442	Proteobacteria	Alphaproteobacteria	Sphingomonadales	unclassified	unclassified
Otu00211	8.462411493	3.949229784	2.95229974	1.337679143	0.181001053	0.982761442	Proteobacteria	Alphaproteobacteria	Sphingomonadales	unclassified	unclassified
Otu00086	15.08138773	2.83199576	1.93913867	1.460440041	0.144169175	0.982761442	Proteobacteria	Alphaproteobacteria	Sphingomonadales	Sphingomonadaceae	Sphingomonas
Otu00274	1.328046068	0.937030369	2.711239947	0.345609532	0.729636182	0.982761442	Proteobacteria	Alphaproteobacteria	Sphingomonadales	Sphingomonadaceae	Sphingomonas
Otu00029	54.75387093	2.650209756	1.743071194	1.520425422	0.128404089	0.982761442	Proteobacteria	Alphaproteobacteria	Sphingomonadales	unclassified	unclassified
Otu00288	0.873824554	0.936604228	2.77178097	0.337907013	0.735433263	0.982761442	Proteobacteria	Alphaproteobacteria	Sphingomonadales	Sphingomonadaceae	Sphingomonas
Otu00491	1.684377593	3.192577032	2.964976081	1.076763166	0.281586085	0.982761442	Proteobacteria	Alphaproteobacteria	Sphingomonadales	Ellin6055	unclassified
Otu00416	0.410569906	2.985962631	2.976230976	1.003269792	0.315730707	0.982761442	Proteobacteria	Alphaproteobacteria	Sphingomonadales	Sphingomonadaceae	Sphingomonas
Otu00305	0.355573108	1.947212681	2.974761866	0.654577667	0.512739707	0.982761442	Proteobacteria	Alphaproteobacteria	Sphingomonadales	Sphingomonadaceae	Sphingomonas

Otu00475	0.596521025	1.714215442	2.970616146	0.577057202	0.563900814	0.982761442	Proteobacteria	Alphaproteobacteria	Sphingomonadales	Sphingomonadaceae	unclassified
Otu00213	2.1898227	-1.448233781	2.193620046	-0.660202656	0.509123788	0.982761442	Proteobacteria	Alphaproteobacteria	Sphingomonadales	unclassified	unclassified
Otu00148	5.196405374	3.488570692	2.956792685	1.179849609	0.238060035	0.982761442	Proteobacteria	Alphaproteobacteria	Sphingomonadales	Erythrobacteraceae	Altererythrobacter
Otu00618	0.928467933	1.955132137	2.965695116	0.659249202	0.509735757	0.982761442	Proteobacteria	Alphaproteobacteria	Sphingomonadales	Erythrobacteraceae	Altererythrobacter
Otu00135	34.19944432	0.505879644	1.769339032	0.285914477	0.774943626	0.982761442	Proteobacteria	Alphaproteobacteria	Sphingomonadales	Sphingomonadaceae	unclassified
Otu00541	0.498523181	-1.068220251	2.966721395	-0.3600676	0.718796582	0.982761442	Proteobacteria	Alphaproteobacteria	Sphingomonadales	Sphingomonadaceae	Novosphingobium
Otu00651	0.679350956	1.671775694	2.971966484	0.562514989	0.573765194	0.982761442	Planctomycetes	Planctomycetacia	Planctomycetales	Planctomycetaceae	Singulisphaera
Otu00342	0.62007824	2.18246832	2.969062748	0.735069787	0.462296994	0.982761442	Planctomycetes	Planctomycetacia	Planctomycetales	Planctomycetaceae	unclassified
Otu00479	0.433011138	1.050820781	2.970938071	0.353699995	0.723563726	0.982761442	Planctomycetes	Planctomycetacia	Planctomycetales	Planctomycetaceae	unclassified
Otu00462	0.765701247	1.759589292	2.967650288	0.592923398	0.553232419	0.982761442	Planctomycetes	Planctomycetacia	Planctomycetales	Planctomycetaceae	unclassified
Otu00538	0.567536049	2.730015544	2.971272979	0.918803342	0.358198444	0.982761442	Planctomycetes	Planctomycetacia	Planctomycetales	Planctomycetaceae	unclassified
Otu00388	0.344988573	1.975820793	2.976633206	0.663777045	0.50683302	0.982761442	Planctomycetes	Planctomycetacia	Planctomycetales	Planctomycetaceae	Singulisphaera
Otu00650	0.596337935	2.066614755	2.972039185	0.695352459	0.486834446	0.982761442	Planctomycetes	Planctomycetacia	Planctomycetales	Planctomycetaceae	Pir4_lineage
Otu00761	0.533058867	1.413109623	2.98002075	0.474194558	0.635361174	0.982761442	Planctomycetes	Planctomycetacia	Planctomycetales	Planctomycetaceae	unclassified
Otu00813	0.316685195	1.529710288	2.978113445	0.513650778	0.607496171	0.982761442	Planctomycetes	Planctomycetacia	Planctomycetales	Planctomycetaceae	unclassified
Otu00493	0.521137644	-0.748069089	2.968321408	-0.25201755	0.801027499	0.982761442	Acidobacteria	Acidobacteria	Subgroup_6	unclassified	unclassified
Otu00324	0.69343472	1.164248483	2.958618957	0.39351079	0.693942256	0.982761442	Acidobacteria	Acidobacteria	Subgroup_4	Unknown_Family	Blastocatella
Otu00420	0.537703124	-1.361437164	2.965026917	-0.459165196	0.646115542	0.982761442	Acidobacteria	Acidobacteria	Subgroup_4	RB41	unclassified
Otu00238	0.986123808	2.996568513	2.969498023	1.009116184	0.312918917	0.982761442	Verrucomicrobia	Spartobacteria	Chthoniobacterales	Xiphinematobacteraceae	Candidatus_Xiphinematobacter
Otu01035	0.489069838	1.458728702	2.984476143	0.48877211	0.625003045	0.982761442	Verrucomicrobia	Spartobacteria	Chthoniobacterales	unclassified	unclassified
Otu00631	0.371680268	1.752000691	2.981404116	0.58764281	0.55677207	0.982761442	Verrucomicrobia	Spartobacteria	Chthoniobacterales	DA101_soil_group	unclassified
Otu00256	1.083354641	-1.417712094	2.58289076	-0.548885812	0.583083815	0.982761442	Verrucomicrobia	Spartobacteria	Chthoniobacterales	DA101_soil_group	unclassified
Otu00261	1.426594283	1.693165808	1.715763327	0.986829466	0.323726247	0.982761442	Verrucomicrobia	Verrucomicrobiae	Verrucomicrobiales	Verrucomicrobiaceae	Luteolibacter
Otu00149	16.60101205	3.663754243	2.952898787	1.240731399	0.214704991	0.982761442	Verrucomicrobia	Verrucomicrobiae	Verrucomicrobiales	Verrucomicrobiaceae	unclassified
Otu00544	1.414613712	2.950001441	2.964539472	0.995096024	0.319689564	0.982761442	Verrucomicrobia	Verrucomicrobiae	Verrucomicrobiales	Verrucomicrobiaceae	unclassified
Otu00171	0.320076833	1.440631145	2.979443688	0.483523535	0.628724053	0.982761442	Verrucomicrobia	OPB35_soil_group	unclassified	unclassified	unclassified
Otu00075	1.700948676	3.078044443	2.96492668	1.03815196	0.299199314	0.982761442	Firmicutes	Negativicutes	Selenomonadales	Veillonellaceae	unclassified
Otu00051	243.6559783	3.731037357	2.477543006	1.505942519	0.13208193	0.982761442	Firmicutes	Negativicutes	Selenomonadales	Veillonellaceae	unclassified
Otu00073	5.730592762	4.159058122	2.955790938	1.407088055	0.15940128	0.982761442	Firmicutes	Negativicutes	Selenomonadales	Veillonellaceae	unclassified
Otu00003	464.568075	-1.420789031	0.924135684	-1.537424705	0.12418934	0.982761442	Fusobacteria	Fusobacteriia	Fusobacteriales	Fusobacteriaceae	Fusobacterium
Otu00143	0.853635132	-0.581455303	1.785566086	-0.325641995	0.744695235	0.982761442	Fusobacteria	Fusobacteriia	Fusobacteriales	Fusobacteriaceae	unclassified
Otu00007	895.9208519	-0.411682932	0.923834786	-0.445623978	0.655868887	0.982761442	Fusobacteria	Fusobacteriia	Fusobacteriales	Fusobacteriaceae	Cetobacterium
Otu00300	3.368513767	3.33253175	2.959269505	1.126133238	0.260109127	0.982761442	Fusobacteria	Fusobacteriia	Fusobacteriales	Fusobacteriaceae	Fusobacterium
Otu00166	5.249393324	3.876163349	2.900491384	1.336381611	0.181424574	0.982761442	Armatimonadetes	unclassified	unclassified	unclassified	unclassified
Otu00314	3.032871066	3.03298178	2.773936027	1.093385626	0.274224522	0.982761442	Firmicutes	Bacilli	Bacillales	Paenibacillaceae	unclassified
Otu00022	114.0967224	-1.535853085	1.050764804	-1.461652578	0.143836438	0.982761442	Firmicutes	Bacilli	Lactobacillales	Enterococcaceae	Enterococcus
Otu00191	2.262385967	-0.735744068	2.666571826	-0.275913838	0.782614245	0.982761442	Firmicutes	Bacilli	Lactobacillales	Aerococcaceae	unclassified
Otu00024	85.9760934	0.659582256	1.099157794	0.600079679	0.548453135	0.982761442	Firmicutes	Bacilli	Lactobacillales	Lactobacillaceae	Lactobacillus
Otu00156	2.659222955	0.906394048	2.946422556	0.307625275	0.758367486	0.982761442	Firmicutes	Bacilli	Lactobacillales	Lactobacillaceae	Lactobacillus
Otu00310	1.034127732	-2.043868265	2.961541908	-0.690136533	0.490108331	0.982761442	Firmicutes	Bacilli	Lactobacillales	Carnobacteriaceae	Dolosigranulum
Otu00317	0.418958985	-1.847253397	2.965567646	-0.622900442	0.533349948	0.982761442	Firmicutes	Bacilli	Lactobacillales	Enterococcaceae	Vagococcus
Otu00357	0.650437942	1.047796872	2.966991621	0.353151275	0.723975035	0.982761442	Firmicutes	Bacilli	Lactobacillales	unclassified	unclassified
Otu00663	0.473625937	0.745661987	2.977385798	0.250441843	0.802245674	0.982761442	Firmicutes	Bacilli	Bacillales	Bacillaceae	unclassified
Otu00250	0.568680866	-1.393000744	2.963257024	-0.470091096	0.638289935	0.982761442	Firmicutes	Bacilli	Bacillales	Bacillaceae	unclassified
Otu00399	2.526123286	3.067126151	2.960646306	1.035965068	0.30021844	0.982761442	Firmicutes	Bacilli	Bacillales	Bacillaceae	Bacillus
Otu00113	17.96886098	1.258535861	2.145983312	0.586461159	0.557565655	0.982761442	Firmicutes	Bacilli	Bacillales	Family_XII	Exiguobacterium
Otu00302	1.169358803	1.635121364	2.959480573	0.552502821	0.580603902	0.982761442	Firmicutes	Bacilli	Bacillales	Planococcaceae	Domibacillus
Otu00340	1.644397784	4.051626366	2.962676506	1.367556113	0.171451062	0.982761442	Firmicutes	Bacilli	Bacillales	Planococcaceae	unclassified
Otu00712	0.500700939	2.184537683	2.979326825	0.733231972	0.463416958	0.982761442	Firmicutes	Bacilli	Bacillales	Bacillaceae	unclassified
Otu00152	2.519596275	-0.942356852	1.802863447	-0.522700071	0.601182989	0.982761442	Firmicutes	Bacilli	Bacillales	Bacillaceae	unclassified
Otu00087	9.943118465	1.338805369	1.212954011	1.103756083	0.26969896	0.982761442	Firmicutes	Bacilli	Bacillales	Planococcaceae	unclassified
Otu00209	3.450249548	2.252886594	1.800694186	1.251121157	0.210890278	0.982761442	Firmicutes	Bacilli	Bacillales	unclassified	unclassified
Otu00045	48.54878085	1.458185545	1.645998555	0.88589722	0.375672914	0.982761442	Firmicutes	Bacilli	Lactobacillales	Streptococcaceae	Streptococcus
Otu00038	267.4782315	3.23073074	2.777914735	1.163005725	0.24482718	0.982761442	Tenericutes	Mollicutes	Mycoplasmatales	Mycoplasmataceae	unclassified
Otu00446	0.390601615	-1.47063638	2.966656247	-0.495721869	0.620090655	0.982761442	Firmicutes	Bacilli	Bacillales	Paenibacillaceae	Paenibacillus
Otu00728	0.32531893	1.298538783	2.979064784	0.435888065	0.662917945	0.982761442	Acidobacteria	Acidobacteria	Acidobacteriales	Acidobacteriaceae_(Subgr	unclassified
Otu00361	0.432003745	-0.886490905	2.969063089	-0.298575974	0.765263599	0.982761442	Deinococcus-Thermus	Deinococci	Deinococcales	Deinococcaceae	Deinococcus
Otu00496	0.445727205	0.877995715	2.971926361	0.295429835	0.767665545	0.982761442	Chloroflexi	Caldilineae	Caldilineales	Caldilineaceae	unclassified
Otu00331	3.40126558	2.981635782	2.958137362	1.007943654	0.313481513	0.982761442	Chloroflexi	TK10	unclassified	unclassified	unclassified
Otu00859	0.646164932	2.127006098	2.974855718	0.714994709	0.474612313	0.982761442	Chloroflexi	Chloroflexia	Kallotenuales	AKIW781	unclassified
Otu00359	0.367000421	2.286400869	2.97816227	0.767722059	0.442652328	0.982761442	Chloroflexi	Thermomicrobia	JG30-KF-CM45	unclassified	unclassified
Otu00321	1.021798227	2.092920146	2.962312542	0.706515642	0.479867529	0.982761442	Chloroflexi	Thermomicrobia	JG30-KF-CM45	unclassified	unclassified
Otu00589	0.477944242	2.060048053	2.972933537	0.692934446	0.488350692	0.982761442	Chloroflexi	Thermomicrobia	JG30-KF-CM45	unclassified	unclassified
Otu00442	0.734079753	3.178541899	2.96781146	1.071005332	0.284167032	0.982761442	Chloroflexi	Thermomicrobia	JG30-KF-CM45	unclassified	unclassified
Otu00262	2.165985971	2.803779354	2.271871519	1.234127604	0.217155359	0.982761442	Chloroflexi	Thermomicrobia	JG30-KF-CM45	unclassified	unclassified
Otu00400	0.652565457	1.371859942	2.96792711	0.462228313	0.643917601	0.982761442	Chloroflexi	Thermomicrobia	JG30-KF-CM45	unclassified	unclassified
Otu00560	0.473093316	3.06117855	2.97508797	1.028937154	0.303509208	0.982761442	unclassified	unclassified	unclassified	unclassified	unclassified
Otu00815	0.441138524	1.957379739	2.976846898	0.657534568	0.510837251	0.982761442	Chloroflexi	KD4-96	unclassified	unclassified	unclassified
Otu00516	0.349978763	0.967930222	2.972889932	0.325585623	0.744737891	0.982761442	Chloroflexi	KD4-96	unclassified	unclassified	unclassified
Otu00518	0.397087629	1.18965212	2.972416917	0.400230571	0.6889867	0.982761442	Chloroflexi	KD4-96	unclassified	unclassified	unclassified
Otu00341	0.676136434	-1.859297773	2.962647388	-0.62757984	0.530279219	0.982761442	Chloroflexi	unclassified	unclassified	unclassified	unclassified
Otu00259	3.370576744	2.987896834	2.82492977	1.05768889	0.290197302	0.982761442	Cyanobacteria	unclassified	unclassified	unclassified	unclassified
Otu00289	0.549836934	1.722975602	2.9702843	0.580070939	0.561866781	0.982761442	Cyanobacteria	Cyanobacteria	SubsectionI	FamilyI	Synechococcus
Otu00364	0.512199047	-0.927398005	2.968077717	-0.312457453	0.754692893	0.982761442	Bacteroidetes	Cytophagia	Cytophagales	Cytophagaceae	Dyadobacter
Otu00200	0.328247534	2.68515367	2.978610762	0.901478536	0.367333941	0.982761442	Bacteroidetes	Cytophagia	Cytophagales	Cytophagaceae	Spirosoma
Otu00358	2.725124164	2.779607676	2.957741557	0.939773683	0.347333661	0.982761442	Bacteroidetes	Cytophagia	Cytophagales	Cytophagaceae	Hymenobacter
Otu00328	0.995968046	2.448953221	2.964438346	0.826110357	0.408741493	0.982761442	Bacteroidetes	Sphingobacteriia	Sphingobacteriales	Sphingobacteriaceae	Mucilaginibacter
Otu00185	3.881221484	2.243446651	2.117444822	1.059506547	0.289369149	0.982761442	Bacteroidetes	Sphingobacteriia	Sphingobacteriales	Sphingobacteriaceae	unclassified
Otu00117	8.780392089	2.156592225	2.933482455	0.735164521	0.462239303	0.982761442	Bacteroidetes	Sphingobacteriia	Sphingobacteriales	Sphingobacteriaceae	Pedobacter
Otu00126	8.641083757	1.309083364	2.770476766	0.472511945	0.636561418	0.982761442	Bacteroidetes	Sphingobacteriia	Sphingobacteriales	Sphingobacteriaceae	Pedobacter
Otu00057	58.54481543	3.66990241	2.923466072	1.255325808	0.20936051	0.982761442	Bacteroidetes	Flavobacteriia	Flavobacteriales	Flavobacteriaceae	Chryseobacterium
Otu00349	1.156827923	2.231758507	2.964673134	0.752784002	0.451579722	0.982761442	Bacteroidetes	Flavobacteriia	Flavobacteriales	Flavobacteriaceae	unclassified
Otu00620	0.344559719	1.645298098	2.975507908	0.552946975	0.58029972	0.982761442	Bacteroidetes	Flavobacteriia	Flavobacteriales	Flavobacteriaceae	Flavobacterium
Otu00047	0.834284385	1.257692018	2.504664109	0.502139993	0.61556905	0.982761442	Bacteroidetes	Flavobacteriia	Flavobacteriales	Flavobacteriaceae	Flavobacterium
Otu00380	0.425391447	1.939806541	2.972503281	0.652583482	0.514024837	0.982761442	Bacteroidetes	Flavobacteriia	Flavobacteriales	Flavobacteriaceae	Flavobacterium
Otu00844	0.475024348	2.495952819	2.979639939	0.837669272	0.402216476	0.982761442	Bacteroidetes	Sphingobacteriia	Sphingobacteriales	Saprospiraceae	unclassified
Otu00506	0.373091022	2.566914903	2.979629367	0.861487986	0.388969335	0.982761442	Bacteroidetes	Sphingobacteriia	Sphingobacteriales	Chitinophagaceae	Taibaiella
Otu00304	0.692766434	0.449372862	1.547700172	0.290348783	0.771549422	0.982761442	Bacteroidetes	Sphingobacteriia	Sphingobacteriales	Chitinophagaceae	unclassified
Otu00033	0.83586902	2.691429738	2.971990388	0.905598399	0.365148456	0.982761442	Bacteroidetes	Bacteroidia	Bacteroidales	Porphyromonadaceae	Macellibacteroides
Otu00118	27.46707442	0.898557082	2.987383826	0.300783941	0.763579255	0.982761442	Bacteroidetes	Bacteroidia	Bacteroidales	Porphyromonadaceae	Dysgonomonas
Otu00031	3.146333301	2.996439476	2.957986466	1.012999725	0.311060292	0.982761442	Bacteroidetes	Bacteroidia	Bacteroidales	Prevotellaceae	Alloprevotella
Otu00443	1.320311449	0.716616286	2.954220537	0.242573727	0.808335636	0.984608205	Actinobacteria	Actinobacteria	Propionibacteriales	Nocardioidaceae	Nocardioides
Otu00082	15.15731967	0.282381227	1.467901733	0.192370661	0.847451872	0.989976703	Actinobacteria	Thermoleophilia	Solirubrobacterales	Solirubrobacteraceae	Solirubrobacter
Otu00542	0.384289054	0.658579485	2.974880645	0.221380137	0.824796457	0.989976703	Actinobacteria	Thermoleophilia	Solirubrobacterales	480-2	unclassified
Otu00180	1.465728496	0.343975165	1.89144572	0.181858333	0.855693905	0.989976703	Actinobacteria	Thermoleophilia	Solirubrobacterales	480-2	unclassified
Otu00512	0.361578713	0.625016819	2.974550408	0.210121441	0.833572891	0.989976703	Actinobacteria	Thermoleophilia	Solirubrobacterales	480-2	unclassified
Otu00114	5.07338925	-0.238343142	1.701155454	-0.140106621	0.88857575	0.989976703	Actinobacteria	Actinobacteria	Pseudonocardiales	Pseudonocardiaceae	Pseudonocardia
Otu00096	6.028315176	0.277326831	1.697057268	0.163416307	0.870190665	0.989976703	Actinobacteria	Actinobacteria	Micrococcales	Micrococcaceae	Rothia
Otu00112	5.594321984	0.217963972	1.504446699	0.144879823	0.884805757	0.989976703	Actinobacteria	Actinobacteria	Micrococcales	Sanguibacteraceae	Sanguibacter
Otu00081	15.36432648	0.28862807	1.497206183	0.192777771	0.847133013	0.989976703	Actinobacteria	Actinobacteria	Propionibacteriales	Nocardioidaceae	Nocardioides
Otu00249	0.868378284	-0.382499517	2.908980336	-0.131489207	0.895388324	0.989976703	Actinobacteria	Actinobacteria	Propionibacteriales	Nocardioidaceae	Marmoricola
Otu00044	26.03633665	-0.23279498	1.76533118	-0.131870429	0.895086779	0.989976703	Firmicutes	Clostridia	Clostridiales	Peptostreptococcaceae	Peptostreptococcus
Otu00004	1368.475293	-0.133202585	0.916611605	-0.14532064	0.884457719	0.989976703	Firmicutes	Clostridia	Clostridiales	Clostridiaceae_1	Clostridium_sensu_stricto_1
Otu00069	1.248067309	0.376722949	2.566237579	0.146799717	0.883290108	0.989976703	Proteobacteria	Gammaproteobacteria	Pseudomonadales	Moraxellaceae	Psychrobacter
Otu00236	0.929929445	-0.510406719	2.297513054	-0.222156178	0.824192306	0.989976703	Proteobacteria	Betaproteobacteria	Burkholderiales	Oxalobacteraceae	Massilia
Otu00108	0.717675932	-0.542599661	2.967940377	-0.18282027	0.854939044	0.989976703	Proteobacteria	Betaproteobacteria	Neisseriales	Neisseriaceae	Neisseria
Otu00480	0.315316681	0.52156165	2.972608074	0.175455908	0.860721413	0.989976703	Proteobacteria	Deltaproteobacteria	Myxococcales	Polyangiaceae	Byssovorax
Otu00405	0.46554909	-0.536307482	2.967274036	-0.1807408	0.856571033	0.989976703	Proteobacteria	Alphaproteobacteria	Rhodospirillales	Acetobacteraceae	Acidiphilium
Otu00167	3.622541416	0.397174471	2.511394544	0.158148974	0.874338941	0.989976703	Proteobacteria	Alphaproteobacteria	Caulobacterales	Caulobacteraceae	Brevundimonas
Otu00376	0.313650486	-0.593184127	2.966529355	-0.199958961	0.841512677	0.989976703	Proteobacteria	Alphaproteobacteria	Rhizobiales	Rhizobiales_Incertae_Sedi	Alsobacter
Otu00132	5.156637352	-0.352304282	1.517544058	-0.232154236	0.816418221	0.989976703	Proteobacteria	Alphaproteobacteria	Rhizobiales	Methylobacteriaceae	Methylobacterium
Otu00141	3.87744812	-0.290688029	1.679840337	-0.173045035	0.862616029	0.989976703	Proteobacteria	Alphaproteobacteria	Rhizobiales	Rhizobiaceae	Rhizobium
Otu00408	0.420862094	0.488731361	2.972418858	0.164422104	0.869398864	0.989976703	Proteobacteria	Alphaproteobacteria	Rhodobacterales	Rhodobacteraceae	unclassified
Otu00391	1.342355662	0.49562765	2.952677592	0.167857016	0.866695767	0.989976703	Acidobacteria	Acidobacteria	Subgroup_4	Unknown_Family	Blastocatella
Otu00202	1.169981868	-0.338384995	1.79226692	-0.18880279	0.850247385	0.989976703	Verrucomicrobia	Spartobacteria	Chthoniobacterales	DA101_soil_group	unclassified
Otu00021	131.6752215	-0.25682356	1.12512819	-0.228261599	0.819442876	0.989976703	Firmicutes	Bacilli	Lactobacillales	Enterococcaceae	Enterococcus
Otu00026	109.59509	0.149319394	1.160230786	0.12869801	0.89759661	0.989976703	Firmicutes	Bacilli	Bacillales	Staphylococcaceae	Staphylococcus

Otu00131	3.445103639	-0.241944158	1.796157278	-0.134700987	0.892848293	0.989976703	Firmicutes	Bacilli	Bacillales	unclassified	unclassified
Otu00308	0.508954747	-0.377604616	2.969942289	-0.127142072	0.898827953	0.989976703	Firmicutes	Bacilli	Bacillales	unclassified	unclassified
Otu00110	5.693446855	0.449517213	2.833570823	0.158639837	0.873952641	0.989976703	Firmicutes	Bacilli	Bacillales	Planococcaceae	unclassified
Otu00054	0.484696845	0.606492262	2.965399289	0.204522967	0.837944846	0.989976703	Firmicutes	Bacilli	Lactobacillales	Streptococcaceae	Lactococcus
Otu00068	4.41495154	0.201778423	1.497367595	0.134755436	0.892805241	0.989976703	Tenericutes	Mollicutes	Mycoplasmatales	Mycoplasmataceae	Ureaplasma
Otu00566	0.433245484	-0.382811979	2.974124371	-0.12871418	0.897583815	0.989976703	Fibrobacteres	Fibrobacteria	Fibrobacterales	Fibrobacteraceae	possible_genus_04
Otu00573	0.346421933	0.603533605	2.972095824	0.203066671	0.839082922	0.989976703	Acidobacteria	Acidobacteria	Subgroup_3	SJA-149	unclassified
Otu00383	0.325864359	0.562818474	2.969059343	0.189561208	0.849652988	0.989976703	WCHB1-60	unclassified	unclassified	unclassified	unclassified
Otu00142	9.870763988	-0.412992388	2.248597743	-0.183666638	0.854274983	0.989976703	Cyanobacteria	Cyanobacteria	SubsectionIII	FamilyI	unclassified
Otu00040	0.644950457	-0.333200248	2.968410073	-0.112248726	0.910626196	0.991889486	Proteobacteria	Betaproteobacteria	unclassified	unclassified	unclassified
Otu00293	0.936915965	0.265200559	2.222401145	0.119330644	0.905013407	0.991889486	Proteobacteria	Alphaproteobacteria	Rhizobiales	Phyllobacteriaceae	unclassified
Otu00569	0.365947552	0.347778855	2.976572418	0.116838701	0.906987875	0.991889486	Proteobacteria	Alphaproteobacteria	Rhizobiales	Rhizobiaceae	Shinella
Otu00027	20.17380473	0.146170945	1.303459998	0.112140722	0.91071183	0.991889486	Firmicutes	Bacilli	Lactobacillales	Streptococcaceae	Streptococcus
Otu00230	0.628022715	-0.24969024	2.358123327	-0.105885149	0.915673477	0.99308681	Actinobacteria	Thermoleophilia	Solirubrobacterales	288-2	unclassified
Otu00109	32.90172373	-0.172779829	1.655758015	-0.104350894	0.916890891	0.99308681	Firmicutes	Bacilli	Bacillales	Planococcaceae	unclassified
Otu00257	0.627003978	-0.194449744	2.967433623	-0.065527917	0.94775368	0.996375142	Actinobacteria	Thermoleophilia	Solirubrobacterales	0319-6M6	unclassified
Otu00322	0.574967431	0.253760337	2.969572875	0.08545348	0.931900878	0.996375142	Actinobacteria	Thermoleophilia	Gaiellales	Gaiellaceae	Gaiella
Otu00266	0.568089375	-0.042242086	2.965067218	-0.014246586	0.988633253	0.996375142	Actinobacteria	Actinobacteria	Micromonosporales	Micromonosporaceae	unclassified
Otu00248	0.916218503	-0.11265275	2.213516292	-0.05089312	0.959410688	0.996375142	Actinobacteria	Actinobacteria	Micromonosporales	Micromonosporaceae	unclassified
Otu00309	0.545366902	0.17388506	2.959580034	0.058753289	0.953148614	0.996375142	Actinobacteria	Actinobacteria	Micromonosporales	Micromonosporaceae	unclassified
Otu00049	3.172354417	-0.089406858	1.248100307	-0.071634353	0.942892901	0.996375142	Actinobacteria	Actinobacteria	Micrococcales	Microbacteriaceae	Amnibacterium
Otu00103	10.79486826	0.031419641	1.690886448	0.018581757	0.985174756	0.996375142	Actinobacteria	Actinobacteria	Frankiales	Geodermatophilaceae	unclassified
Otu00222	0.880325036	0.218135534	2.951648188	0.073902959	0.941087602	0.996375142	Actinobacteria	Actinobacteria	unclassified	unclassified	unclassified
Otu00476	0.34818929	-0.020923221	2.972996353	-0.007037755	0.99438473	0.996375142	Actinobacteria	Acidimicrobiia	Acidimicrobiales	unclassified	unclassified
Otu00125	0.414865377	0.064754876	2.974081316	0.021773068	0.982628977	0.996375142	Proteobacteria	Gammaproteobacteria	Pseudomonadales	Pseudomonadaceae	Pseudomonas
Otu00546	0.381513573	0.16760529	2.972166417	0.056391624	0.95502983	0.996375142	Proteobacteria	Gammaproteobacteria	Cellvibrionales	Cellvibrionaceae	Cellvibrio
Otu00127	6.038200836	-0.052803588	1.921615502	-0.027478748	0.97807789	0.996375142	Proteobacteria	Betaproteobacteria	Burkholderiales	Comamonadaceae	unclassified
Otu00329	0.359376563	0.051971237	2.97106028	0.017492488	0.986043725	0.996375142	Proteobacteria	Deltaproteobacteria	Oligoflexales	Oligoflexaceae	Oligoflexus
Otu00365	0.341486554	0.184057511	2.967079174	0.062033232	0.950536368	0.996375142	Proteobacteria	Alphaproteobacteria	Rhizobiales	unclassified	unclassified
Otu00120	3.849632301	-0.006161891	1.356318175	-0.004543101	0.996375142	0.996375142	Proteobacteria	Alphaproteobacteria	Rhizobiales	Bradyrhizobiaceae	unclassified
Otu00732	0.344795976	-0.03969344	2.977518608	-0.013331047	0.989363679	0.996375142	Proteobacteria	Alphaproteobacteria	Rhizobiales	JG34-KF-361	unclassified
Otu00343	0.441603765	0.085515117	2.965208332	0.028839497	0.9769926	0.996375142	Proteobacteria	Alphaproteobacteria	unclassified	unclassified	unclassified
Otu00199	0.985851321	0.08190537	2.964156101	0.027631936	0.977955711	0.996375142	Proteobacteria	Alphaproteobacteria	Rhodobacterales	Rhodobacteraceae	unclassified
Otu00115	35.30162363	0.068315092	1.691006556	0.04039907	0.967774972	0.996375142	Proteobacteria	Alphaproteobacteria	Rhodobacterales	Rhodobacteraceae	unclassified
Otu00505	0.385692465	0.278426854	2.972203739	0.093676907	0.925365815	0.996375142	Proteobacteria	Alphaproteobacteria	Rhodobacterales	Rhodobacteraceae	unclassified
Otu00313	0.745508768	0.177252106	2.954915655	0.059985504	0.95216718	0.996375142	Proteobacteria	Alphaproteobacteria	Sphingomonadales	Sphingomonadaceae	Sphingomonas
Otu00246	0.725681185	-0.038417217	2.675942189	-0.01435652	0.988545548	0.996375142	Proteobacteria	Alphaproteobacteria	Sphingomonadales	Sphingomonadaceae	Sphingomonas
Otu00172	0.498807272	0.068584801	2.964090336	0.023138566	0.981539742	0.996375142	Planctomycetes	Planctomycetacia	Planctomycetales	Planctomycetaceae	Pir4_lineage
Otu00390	0.739792078	-0.214457866	2.962825064	-0.072382899	0.942297194	0.996375142	Acidobacteria	Acidobacteria	Subgroup_6	unclassified	unclassified
Otu00197	0.977966829	0.151059497	2.962146456	0.050996633	0.959328203	0.996375142	Verrucomicrobia	Spartobacteria	Chthoniobacterales	Chthoniobacteraceae	Chthoniobacter
Otu00168	0.818778412	-0.051568318	2.966085344	-0.017385986	0.986128689	0.996375142	Firmicutes	Bacilli	Lactobacillales	unclassified	unclassified
Otu00234	0.765880814	-0.081887183	2.958469938	-0.027678896	0.977918256	0.996375142	Firmicutes	Bacilli	Bacillales	Bacillaceae	Bacillus
Otu00356	0.611716164	-0.090604558	2.652623196	-0.034156588	0.972752284	0.996375142	Firmicutes	Bacilli	Bacillales	Planococcaceae	unclassified
Otu00653	0.372384786	0.27485402	2.975183188	0.092382217	0.926394367	0.996375142	Chloroflexi	TK10	unclassified	unclassified	unclassified
Otu00345	0.323848449	0.024013269	2.972657914	0.008078047	0.993554721	0.996375142	Cyanobacteria	Cyanobacteria	SubsectionII	FamilyII	Pleurocapsa
Otu00980	0.310519044	1.576422349	2.97905453	0.529168679	0.596688442	NA	Cyanobacteria	ML635J-21	unclassified	unclassified	unclassified
Otu01196	0.042690782	0.585937945	2.986414272	0.19620116	0.84445272	NA	Cyanobacteria	Melainabacteria	Gastranaerophilales	unclassified	unclassified
Otu00525	0.153358398	0.198070943	2.978042999	0.066510438	0.946971448	NA	Actinobacteria	Thermoleophilia	Gaiellales	unclassified	unclassified
Otu00627	0.124060073	0.424994423	2.979534186	0.142637875	0.886576184	NA	Actinobacteria	Thermoleophilia	Solirubrobacterales	Solirubrobacteraceae	Solirubrobacter
Otu00780	0.075558789	0.504199059	2.981766661	0.169094069	0.86572265	NA	Actinobacteria	Thermoleophilia	Solirubrobacterales	Solirubrobacteraceae	Solirubrobacter
Otu00673	0.095568896	0.40735091	2.980517956	0.136671181	0.89129071	NA	Actinobacteria	Thermoleophilia	Solirubrobacterales	unclassified	unclassified
Otu00879	0.064621265	0.950225999	2.983280062	0.318517196	0.750092653	NA	Actinobacteria	Thermoleophilia	Solirubrobacterales	288-2	unclassified
Otu01129	0.037779395	0.741251105	2.984977935	0.248327164	0.803881283	NA	Actinobacteria	Thermoleophilia	Solirubrobacterales	Elev-16S-1332	unclassified
Otu00812	0.101671278	0.902470506	2.985831923	0.30225094	0.762460774	NA	Actinobacteria	Thermoleophilia	Solirubrobacterales	480-2	unclassified
Otu01023	0.130073613	0.839141329	2.986311749	0.28099589	0.778713552	NA	Actinobacteria	Thermoleophilia	Solirubrobacterales	480-2	unclassified
Otu00791	0.16081182	1.722136306	2.985021743	0.576925883	0.563989525	NA	Actinobacteria	Thermoleophilia	Solirubrobacterales	480-2	unclassified
Otu01009	0.052449191	0.451633559	2.984439375	0.151329447	0.879715842	NA	Actinobacteria	Thermoleophilia	Solirubrobacterales	Elev-16S-1332	unclassified
Otu00485	0.186544422	-0.166162857	2.97611505	-0.055832135	0.955475535	NA	Actinobacteria	Thermoleophilia	Solirubrobacterales	unclassified	unclassified
Otu00993	0.283825688	1.877105601	2.983658674	0.6291288	0.529264737	NA	Actinobacteria	Thermoleophilia	Solirubrobacterales	480-2	unclassified
Otu00797	0.181776227	1.758131631	2.980254979	0.589926581	0.555239872	NA	Actinobacteria	Thermoleophilia	Solirubrobacterales	0319-6M6	unclassified
Otu00798	0.130858905	0.995423689	2.980364537	0.333993938	0.738384133	NA	Actinobacteria	Thermoleophilia	Solirubrobacterales	0319-6M6	unclassified
Otu01001	0.069100476	1.283487579	2.985482032	0.429909665	0.667261355	NA	Actinobacteria	Thermoleophilia	Solirubrobacterales	unclassified	unclassified
Otu00737	0.250181608	1.989231614	2.981923505	0.667096795	0.504710307	NA	Actinobacteria	Thermoleophilia	Solirubrobacterales	unclassified	unclassified
Otu01166	0.159248512	1.26872928	2.983916917	0.425189211	0.670698748	NA	Actinobacteria	Thermoleophilia	Solirubrobacterales	unclassified	unclassified
Otu00656	0.24915521	1.774214522	2.980894629	0.595195317	0.551712921	NA	Actinobacteria	Thermoleophilia	Solirubrobacterales	unclassified	unclassified
Otu00551	0.187407907	1.025846455	2.97741867	0.344542226	0.730438546	NA	Actinobacteria	Thermoleophilia	Solirubrobacterales	Patulibacteraceae	Patulibacter
Otu01378	0.037779395	0.741251105	2.984977935	0.248327164	0.803881283	NA	Actinobacteria	Thermoleophilia	Gaiellales	unclassified	unclassified
Otu00900	0.022472707	0.638656808	2.988914003	0.213675204	0.830800349	NA	Actinobacteria	Thermoleophilia	Gaiellales	unclassified	unclassified
Otu00559	0.174961347	0.024026509	2.976525925	0.008071997	0.993559548	NA	Actinobacteria	Thermoleophilia	Gaiellales	unclassified	unclassified
Otu00919	0.134103481	0.910962433	2.985267306	0.305152718	0.760249841	NA	Actinobacteria	Thermoleophilia	Gaiellales	unclassified	unclassified
Otu01032	0.055517327	0.727764691	2.989283964	0.243457865	0.807650722	NA	Actinobacteria	Thermoleophilia	Gaiellales	unclassified	unclassified
Otu00588	0.124146618	0.241214827	2.977970791	0.080999729	0.935442168	NA	Actinobacteria	Rubrobacteria	Rubrobacterales	Rubrobacteriaceae	Rubrobacter
Otu00582	0.143680217	-0.601630288	2.976624287	-0.202118316	0.839824229	NA	Actinobacteria	Rubrobacteria	Rubrobacterales	Rubrobacteriaceae	Rubrobacter
Otu00502	0.205347739	-0.119450979	2.976822824	-0.040127003	0.967991874	NA	Actinobacteria	Actinobacteria	Micromonosporales	Micromonosporaceae	unclassified
Otu00968	0.165105478	1.805104042	2.984325707	0.604861607	0.545270955	NA	Actinobacteria	Actinobacteria	Micromonosporales	Micromonosporaceae	Dactylosporangium
Otu01207	0.037779395	0.741251105	2.984977935	0.248327164	0.803881283	NA	Actinobacteria	Actinobacteria	Pseudonocardiales	Pseudonocardiaceae	Lentzea
Otu00695	0.197094734	1.190767812	2.980011001	0.399585039	0.689462178	NA	Actinobacteria	Actinobacteria	unclassified	unclassified	unclassified
Otu00577	0.252417614	0.74552634	2.976744352	0.250450241	0.802239181	NA	Actinobacteria	Actinobacteria	Streptomycetales	Streptomycetaceae	Streptomyces
Otu00494	0.307314268	0.303267128	2.97440997	0.101958752	0.918789416	NA	Actinobacteria	Actinobacteria	Streptomycetales	Streptomycetaceae	unclassified
Otu00581	0.211836745	1.653432717	2.981390956	0.554584334	0.579179007	NA	Actinobacteria	Actinobacteria	Frankiales	Cryptosporangiaceae	Cryptosporangium
Otu00609	0.135742389	-0.452471281	2.977038543	-0.151987042	0.879197156	NA	Actinobacteria	Actinobacteria	unclassified	unclassified	unclassified
Otu00953	0.063834298	0.779692247	2.986580071	0.261065241	0.794042196	NA	Actinobacteria	Actinobacteria	Kineosporiales	Kineosporiaceae	unclassified
Otu00065	0.00787546	1.019097991	2.98881957	0.340970061	0.73312612	NA	Actinobacteria	Actinobacteria	Actinomycetales	Actinomycetaceae	Actinomyces
Otu00450	0.278499979	1.435864947	2.975914091	0.482495429	0.629454045	NA	Actinobacteria	Actinobacteria	Actinomycetales	Actinomycetaceae	unclassified
Otu01003	0.180054734	1.832798183	2.98433734	0.614139078	0.539123427	NA	Actinobacteria	Actinobacteria	Micrococcales	Beutenbergiaceae	Salana
Otu01049	0.080248837	0.486537883	2.981528169	0.163184064	0.870373513	NA	Actinobacteria	Actinobacteria	Micrococcales	Microbacteriaceae	unclassified
Otu00431	0.104291221	1.0453136	2.983261718	0.350392858	0.726043886	NA	Actinobacteria	Actinobacteria	Kineosporiales	Kineosporiaceae	Kineosporia
Otu01079	0.191068772	1.203301391	2.986251218	0.402947141	0.686987111	NA	Actinobacteria	Actinobacteria	unclassified	unclassified	unclassified
Otu00454	0.221479825	-0.929585502	2.971992443	-0.31278192	0.754446352	NA	Actinobacteria	Actinobacteria	Frankiales	Geodermatophilaceae	unclassified
Otu00690	0.066135398	0.675925776	2.982674688	0.226617331	0.820721315	NA	Actinobacteria	Actinobacteria	Corynebacteriales	Nocardiaceae	Nocardia
Otu00591	0.151493455	0.70928178	2.978407904	0.23814125	0.811771542	NA	Actinobacteria	Actinobacteria	Corynebacteriales	Nocardiaceae	Rhodococcus
Otu00923	0.068670366	0.849684238	2.984224901	0.284725269	0.77585463	NA	Actinobacteria	Actinobacteria	Corynebacteriales	Mycobacteriaceae	Mycobacterium
Otu00017	0.019422142	0.909547968	2.987568111	0.304444262	0.760789451	NA	Actinobacteria	Actinobacteria	Corynebacteriales	Corynebacteriaceae	Corynebacterium
Otu00705	0.095568896	0.40735091	2.980517956	0.136671181	0.89129071	NA	Actinobacteria	Actinobacteria	Corynebacteriales	Corynebacteriaceae	Corynebacterium
Otu00704	0.176124189	-0.041621056	2.97752743	-0.013978396	0.988847217	NA	Actinobacteria	Actinobacteria	Frankiales	Geodermatophilaceae	unclassified
Otu00753	0.029331346	1.446566117	2.987085314	0.484273452	0.628191814	NA	Actinobacteria	Actinobacteria	Streptosporangiales	Thermomonosporaceae	Actinomadura
Otu00809	0.070919576	0.333582584	2.982797331	0.111835484	0.910953852	NA	Actinobacteria	Actinobacteria	Streptosporangiales	Thermomonosporaceae	Actinocorallia
Otu01372	0.031902721	1.108649166	2.986737713	0.371190668	0.710495523	NA	Actinobacteria	Actinobacteria	Streptosporangiales	Thermomonosporaceae	Actinoallomurus
Otu00514	0.184791212	0.568405481	2.977009152	0.190931721	0.848579087	NA	Actinobacteria	Actinobacteria	Streptosporangiales	Streptosporangiaceae	Streptosporangium
Otu01025	0.057042562	0.612459416	2.983300104	0.205295946	0.837340911	NA	Actinobacteria	Actinobacteria	Streptosporangiales	Streptosporangiaceae	Microbispora
Otu01303	0.022791153	0.783255429	2.987384171	0.262187715	0.793176724	NA	Actinobacteria	Actinobacteria	Propionibacteriales	Nocardioidaceae	Marmoricola
Otu00735	0.215385159	0.883758206	2.978973866	0.29666531	0.766722044	NA	Actinobacteria	Actinobacteria	Propionibacteriales	Nocardioidaceae	Nocardioides
Otu00616	0.232521201	-0.630539312	2.971846131	-0.212170915	0.831973697	NA	Actinobacteria	Actinobacteria	Propionibacteriales	Nocardioidaceae	Nocardioides
Otu01384	0.037779395	0.741251105	2.984977935	0.248327164	0.803881283	NA	Actinobacteria	Actinobacteria	Propionibacteriales	Nocardioidaceae	Kribbella
Otu00702	0.147961536	0.748960653	2.981482005	0.25120415	0.801656276	NA	Actinobacteria	Actinobacteria	Frankiales	Geodermatophilaceae	unclassified
Otu00849	0.116910228	0.916592003	2.980343984	0.307545709	0.758428037	NA	Actinobacteria	Actinobacteria	Frankiales	Frankiaceae	Jatrophihabitans
Otu00606	0.133017195	1.224435318	2.979497521	0.410953629	0.681106537	NA	Actinobacteria	Actinobacteria	Frankiales	unclassified	unclassified
Otu00536	0.200911779	0.883561705	2.97667987	0.296827924	0.766597886	NA	Actinobacteria	Actinobacteria	Frankiales	unclassified	unclassified
Otu00245	0.302983337	1.315013777	2.97493127	0.44203165	0.658466303	NA	Actinobacteria	Actinobacteria	Frankiales	unclassified	unclassified
Otu00774	0.197933822	0.980194568	2.980163662	0.328906288	0.742226521	NA	Actinobacteria	Acidimicrobiia	Acidimicrobiales	Acidimicrobiaceae	Illumatobacter
Otu00492	0.294567608	0.619459179	2.974455394	0.208259697	0.835026199	NA	Actinobacteria	Acidimicrobiia	Acidimicrobiales	Acidimicrobiaceae	Illumatobacter
Otu00558	0.250192876	0.455110453	2.97789701	0.152829481	0.878532749	NA	Actinobacteria	Acidimicrobiia	Acidimicrobiales	Acidimicrobiales_Incertae_	Candidatus_Microthrix
Otu00598	0.142420873	0.496825485	2.978488602	0.166804561	0.86752383	NA	Actinobacteria	Acidimicrobiia	Acidimicrobiales	Iamiaceae	Iamia
Otu00639	0.246117155	0.844197369	2.978210119	0.283457961	0.776825803	NA	Actinobacteria	Acidimicrobiia	Acidimicrobiales	Iamiaceae	Iamia
Otu00192	0.119890488	0.321988106	2.978676829	0.108097697	0.913918195	NA	Actinobacteria	Acidimicrobiia	Acidimicrobiales	Iamiaceae	Iamia
Otu00637	0.234648326	0.804622606	2.977733167	0.270213132	0.78699629	NA	Actinobacteria	Acidimicrobiia	Acidimicrobiales	Iamiaceae	Iamia
Otu00807	0.063805443	1.091774066	2.983262704	0.365966452	0.714390109	NA	Actinobacteria	unclassified	unclassified	unclassified	unclassified

Otu00914	0.163364462	1.118302239	2.983406498	0.37484072	0.707778929	NA	Actinobacteria	unclassified	unclassified	unclassified	unclassified
Otu00188	0.023359873	0.866090981	2.986852341	0.289967793	0.771840877	NA	Firmicutes	Clostridia	Clostridiales	unclassified	unclassified
Otu01282	0.035582271	0.559858777	2.988441231	0.187341404	0.851392962	NA	Firmicutes	Clostridia	Clostridiales	Family_XI	Gallicola
Otu00297	0.260306898	-1.189435872	2.97021307	-0.40045473	0.68882162	NA	Firmicutes	Clostridia	Clostridiales	Peptostreptococcaceae	unclassified
Otu00703	0.087987767	0.219490883	2.981118826	0.073627016	0.941307174	NA	Firmicutes	Clostridia	Clostridiales	Peptostreptococcaceae	Terrisporobacter
Otu01045	0.056295622	0.616681692	2.983351327	0.2067077	0.836238143	NA	Firmicutes	Clostridia	Clostridiales	Lachnospiraceae	unclassified
Otu01340	0.025934339	0.727764692	2.989283964	0.243457865	0.807650722	NA	Firmicutes	Clostridia	Clostridiales	Lachnospiraceae	Lachnoclostridium
Otu00605	0.111097365	0.342435967	2.979757929	0.114920734	0.908507951	NA	Firmicutes	Clostridia	Clostridiales	Clostridiaceae_1	Clostridium_sensu_stricto_1
Otu00632	0.096856062	0.30801599	2.980448579	0.103345514	0.917688754	NA	Firmicutes	Clostridia	Clostridiales	Clostridiaceae_1	Clostridium_sensu_stricto_1
Otu01336	0.008212655	0.925997581	2.989138024	0.309787495	0.756722562	NA	Firmicutes	Clostridia	Clostridiales	Clostridiaceae_1	unclassified
Otu01174	0.012701833	0.973309715	2.988458937	0.325689506	0.744659285	NA	Firmicutes	Clostridia	Clostridiales	Clostridiaceae_1	unclassified
Otu00269	0.311577479	-1.00990712	2.969261022	-0.340120694	0.733765638	NA	Firmicutes	Clostridia	Clostridiales	Clostridiaceae_1	Clostridium_sensu_stricto_1
Otu00426	0.194634506	-0.83459443	2.973369755	-0.280689756	0.778948367	NA	Firmicutes	Clostridia	Clostridiales	Clostridiaceae_1	unclassified
Otu00870	0.078063397	1.256987344	2.98236378	0.421473514	0.673409348	NA	Firmicutes	Clostridia	Clostridiales	Clostridiaceae_1	Clostridium_sensu_stricto_1
Otu00009	0.292456099	0.183270741	2.978455595	0.061532138	0.95093542	NA	Firmicutes	Clostridia	Clostridiales	Ruminococcaceae	Faecalibacterium
Otu00006	0.098494791	0.983067975	2.980941356	0.329784406	0.741562871	NA	Firmicutes	Clostridia	Clostridiales	Ruminococcaceae	Subdoligranulum
Otu00437	0.260763089	-0.514759274	2.973592137	-0.173110249	0.862564769	NA	Proteobacteria	Deltaproteobacteria	Myxococcales	Haliangiaceae	Haliangium
Otu00678	0.1507892	0.419127553	2.9825551	0.140526341	0.888244142	NA	Proteobacteria	Deltaproteobacteria	Myxococcales	Haliangiaceae	Haliangium
Otu00531	0.234719763	0.604916397	2.979120467	0.203052009	0.839094381	NA	Proteobacteria	Deltaproteobacteria	Myxococcales	Nannocystaceae	unclassified
Otu01396	0.039031698	1.202390796	2.986284084	0.402637781	0.687214712	NA	Proteobacteria	Deltaproteobacteria	Myxococcales	Nannocystaceae	Nannocystis
Otu01184	0.016812502	1.33555333	2.988061427	0.446963144	0.654901671	NA	Proteobacteria	Deltaproteobacteria	Oligoflexales	unclassified	unclassified
Otu00513	0.082359078	0.698185216	2.983850762	0.233987981	0.814994309	NA	Proteobacteria	Gammaproteobacteria	Xanthomonadales	Xanthomonadaceae	Rhodanobacter
Otu00905	0.217517105	1.206935911	2.986261529	0.40416283	0.686092985	NA	Proteobacteria	Gammaproteobacteria	Xanthomonadales	Xanthomonadaceae	Luteibacter
Otu01289	0.033887121	0.627076147	2.987078994	0.209929549	0.833722659	NA	Proteobacteria	Gammaproteobacteria	Xanthomonadales	Xanthomonadaceae	unclassified
Otu00960	0.055078767	1.66961151	2.985253472	0.559286347	0.575966312	NA	Proteobacteria	Gammaproteobacteria	Xanthomonadales	Xanthomonadaceae	Lysobacter
Otu00218	0.115579003	0.325694682	2.979571318	0.109309242	0.912957217	NA	Proteobacteria	Gammaproteobacteria	Xanthomonadales	Xanthomonadaceae	unclassified
Otu00392	0.272714235	0.288742015	2.974090754	0.097085812	0.922658248	NA	Proteobacteria	Gammaproteobacteria	Xanthomonadales	Xanthomonadaceae	Pseudoxanthomonas
Otu01014	0.178352704	1.110848304	2.981880172	0.372532845	0.709496162	NA	Proteobacteria	Gammaproteobacteria	Xanthomonadales	Xanthomonadaceae	Arenimonas
Otu00912	0.04130738	0.620964133	2.984808326	0.208041544	0.835196529	NA	Proteobacteria	Gammaproteobacteria	Xanthomonadales	Xanthomonadaceae	Mizugakiibacter
Otu00885	0.063772265	1.572555538	2.986208071	0.526606151	0.59846712	NA	Proteobacteria	Gammaproteobacteria	Pseudomonadales	Pseudomonadaceae	unclassified
Otu00646	0.114991039	0.327862381	2.979595279	0.110035878	0.912380921	NA	Proteobacteria	Gammaproteobacteria	Pseudomonadales	Pseudomonadaceae	unclassified
Otu00913	0.086383631	0.466373429	2.98601488	0.156185903	0.875886488	NA	Proteobacteria	Gammaproteobacteria	unclassified	unclassified	unclassified
Otu00830	0.206465387	1.408439186	2.984856856	0.471861551	0.637025614	NA	Proteobacteria	Gammaproteobacteria	Xanthomonadales	Xanthomonadales_Incerta	Steroidobacter
Otu01149	0.107590428	1.203301382	2.986251218	0.402947138	0.686987113	NA	Proteobacteria	unclassified	unclassified	unclassified	unclassified
Otu01215	0.070525734	0.679552263	2.984143738	0.227721022	0.819863128	NA	Proteobacteria	unclassified	unclassified	unclassified	unclassified
Otu00406	0.273868408	0.703394401	2.971932579	0.236679125	0.812905731	NA	Proteobacteria	Gammaproteobacteria	Legionellales	Legionellaceae	Legionella
Otu00800	0.192694288	1.960433312	2.983410442	0.657111501	0.511109225	NA	Proteobacteria	Gammaproteobacteria	Legionellales	Legionellaceae	Legionella
Otu00028	0.051242402	0.831707073	2.989467755	0.278212425	0.780849298	NA	Proteobacteria	Gammaproteobacteria	Legionellales	Legionellaceae	Legionella
Otu00537	0.058264309	0.165643027	2.984127843	0.05550802	0.955733741	NA	Proteobacteria	Gammaproteobacteria	Aeromonadales	Aeromonadaceae	Tolumonas
Otu00061	0.096232086	1.974975081	2.98317234	0.662038547	0.507946518	NA	Proteobacteria	Gammaproteobacteria	Pasteurellales	Pasteurellaceae	Avibacterium
Otu00793	0.074970825	0.507232632	2.981808128	0.170109078	0.864924354	NA	Proteobacteria	Gammaproteobacteria	Vibrionales	Vibrionaceae	unclassified
Otu00207	0.14315713	1.054925491	2.977691729	0.354276261	0.723131857	NA	Proteobacteria	Gammaproteobacteria	Pseudomonadales	Moraxellaceae	Psychrobacter
Otu00964	0.044542275	1.264571824	2.985742867	0.423536748	0.671903693	NA	Proteobacteria	Betaproteobacteria	Burkholderiales	Burkholderiaceae	Burkholderia
Otu00467	0.242811271	-1.079891594	2.972192968	-0.363331589	0.716357199	NA	Proteobacteria	Betaproteobacteria	Burkholderiales	Alcaligenaceae	unclassified
Otu01176	0.008110678	1.109179264	2.988776319	0.371114846	0.710551994	NA	Proteobacteria	Betaproteobacteria	Burkholderiales	Alcaligenaceae	unclassified
Otu01410	0.022689176	0.959385237	2.987257656	0.321159186	0.74808976	NA	Proteobacteria	Betaproteobacteria	Burkholderiales	Alcaligenaceae	Castellaniella
Otu01089	0.073825564	1.450986405	2.987032108	0.485761904	0.627135987	NA	Proteobacteria	Betaproteobacteria	Burkholderiales	unclassified	unclassified
Otu00684	0.090700803	1.03484047	2.983978803	0.346798868	0.728742428	NA	Proteobacteria	Betaproteobacteria	Burkholderiales	Oxalobacteraceae	unclassified
Otu00196	0.021659414	0.831707086	2.989467755	0.278212429	0.780849294	NA	Proteobacteria	Betaproteobacteria	Burkholderiales	Comamonadaceae	Aquabacterium
Otu01177	0.025597144	0.782255846	2.989188148	0.261695085	0.79355653	NA	Proteobacteria	Betaproteobacteria	Burkholderiales	Comamonadaceae	unclassified
Otu01055	0.25886096	1.553422977	2.982089757	0.520917579	0.602424189	NA	Proteobacteria	Betaproteobacteria	Burkholderiales	Comamonadaceae	unclassified
Otu00373	0.07748926	-0.084031421	2.981975996	-0.028179778	0.977518766	NA	Proteobacteria	Betaproteobacteria	Hydrogenophilales	Hydrogenophilaceae	Hydrogenophilus
Otu01034	0.070551025	0.505603231	2.988224547	0.16919854	0.865640478	NA	Proteobacteria	Betaproteobacteria	SC-I-84	unclassified	unclassified
Otu00907	0.05541535	0.881820691	2.989047819	0.295017258	0.767980696	NA	Proteobacteria	Betaproteobacteria	SC-I-84	unclassified	unclassified
Otu01332	0.016099402	0.727764692	2.989283964	0.243457865	0.807650722	NA	Proteobacteria	Gammaproteobacteria	Pseudomonadales	Moraxellaceae	Acinetobacter
Otu00643	0.277003426	1.174965295	2.984489994	0.393690479	0.693809572	NA	Proteobacteria	Gammaproteobacteria	Cellvibrionales	Halieaceae	unclassified
Otu01258	0.037030307	0.559858773	2.988441231	0.187341403	0.851392963	NA	Proteobacteria	Deltaproteobacteria	unclassified	unclassified	unclassified
Otu01259	0.069758629	0.627076146	2.987078994	0.209929549	0.833722659	NA	Proteobacteria	Deltaproteobacteria	Myxococcales	Polyangiaceae	Sorangium
Otu00281	0.211315633	1.519767796	2.976712568	0.510552417	0.609664502	NA	Proteobacteria	Deltaproteobacteria	Myxococcales	BIrii41	unclassified
Otu00667	0.115693192	1.223304676	2.983155978	0.410070638	0.681754131	NA	Proteobacteria	Deltaproteobacteria	Myxococcales	Sandaracinaceae	unclassified
Otu00766	0.059486412	0.596882932	2.983102615	0.200087965	0.841411785	NA	Proteobacteria	Deltaproteobacteria	Myxococcales	Sandaracinaceae	unclassified
Otu00565	0.148765027	-0.043006175	2.977847943	-0.014442032	0.988477326	NA	Proteobacteria	Deltaproteobacteria	Myxococcales	Sandaracinaceae	unclassified
Otu00596	0.123244251	0.549038396	2.979079908	0.184297976	0.853779702	NA	Proteobacteria	Deltaproteobacteria	Myxococcales	Sandaracinaceae	unclassified
Otu00389	0.121075646	1.623095193	2.983054175	0.544105168	0.586369104	NA	Proteobacteria	Deltaproteobacteria	Myxococcales	unclassified	unclassified
Otu00048	0.238766913	1.68049767	2.985558984	0.562875388	0.573519741	NA	Proteobacteria	Deltaproteobacteria	Desulfovibrionales	Desulfovibrionaceae	Desulfovibrio
Otu00773	0.070965419	0.323800972	2.982645502	0.108561668	0.913550165	NA	Proteobacteria	Deltaproteobacteria	Oligoflexales	Oligoflexaceae	Oligoflexus
Otu01301	0.21324218	1.329944801	2.986459696	0.445324878	0.656084992	NA	Proteobacteria	Epsilonproteobacteria	Campylobacterales	Campylobacteraceae	Campylobacter
Otu00037	0.170460456	1.859090271	2.983979095	0.623023892	0.533268821	NA	Proteobacteria	Epsilonproteobacteria	Campylobacterales	Campylobacteraceae	Campylobacter
Otu00817	0.109031903	0.318974089	2.982571148	0.106946012	0.91483181	NA	Proteobacteria	Alphaproteobacteria	Rhodospirillales	DA111	unclassified
Otu00920	0.061835055	0.297314515	2.985795219	0.099576325	0.92068069	NA	Proteobacteria	Alphaproteobacteria	Rhodospirillales	Acetobacteraceae	unclassified
Otu00767	0.124041089	0.324658293	2.9834639	0.108819246	0.913345858	NA	Proteobacteria	Alphaproteobacteria	Rhodospirillales	Acetobacteraceae	unclassified
Otu00370	0.15647641	2.25865413	2.981533478	0.757547801	0.448721739	NA	Proteobacteria	Alphaproteobacteria	Rhodospirillales	Acetobacteraceae	unclassified
Otu00769	0.176752493	-0.364096612	2.977760689	-0.122271952	0.902683644	NA	Proteobacteria	Alphaproteobacteria	Rhodospirillales	Acetobacteraceae	unclassified
Otu00709	0.075698057	1.230636639	2.982417684	0.412630546	0.679877318	NA	Proteobacteria	Alphaproteobacteria	Rhodospirillales	Acetobacteraceae	Roseococcus
Otu00698	0.294460967	0.731569145	2.980643635	0.24543999	0.806115768	NA	Proteobacteria	Alphaproteobacteria	Rhodospirillales	Acetobacteraceae	Roseomonas
Otu00610	0.148184067	0.484010972	2.979498013	0.162447154	0.870953741	NA	Proteobacteria	Alphaproteobacteria	Rhodospirillales	Acetobacteraceae	unclassified
Otu01377	0.10080784	1.570268803	2.986055251	0.525867297	0.598980412	NA	Proteobacteria	Alphaproteobacteria	Rhodospirillales	Rhodospirillaceae	Dongia
Otu01181	0.031087644	0.619581693	2.986955691	0.207429154	0.835674716	NA	Proteobacteria	Alphaproteobacteria	Rhodospirillales	Rhodospirillaceae	Defluviicoccus
Otu01359	0.063881956	1.005911733	2.988253145	0.336621994	0.736401874	NA	Proteobacteria	Alphaproteobacteria	Rhodospirillales	Rhodospirillales_Incertae_	Candidatus_Alysiosphaera
Otu00374	0.263060728	0.198122334	2.973413436	0.066631277	0.946875246	NA	Proteobacteria	Alphaproteobacteria	Rhodospirillales	Rhodospirillales_Incertae_	Candidatus_Alysiosphaera
Otu00101	0.019263167	0.911196952	2.987595952	0.304993368	0.760371203	NA	Proteobacteria	Alphaproteobacteria	Rhizobiales	A0839	unclassified
Otu00194	0.092225359	-0.129419457	2.980383229	-0.043423764	0.965363735	NA	Proteobacteria	Alphaproteobacteria	Caulobacterales	Caulobacteraceae	unclassified
Otu00457	0.267836538	-0.978034339	2.971080086	-0.329184778	0.742016028	NA	Proteobacteria	Alphaproteobacteria	Caulobacterales	Caulobacteraceae	unclassified
Otu00729	0.099720662	0.615681838	2.982587681	0.206425394	0.836458636	NA	Proteobacteria	Alphaproteobacteria	Caulobacterales	Caulobacteraceae	Caulobacter
Otu01317	0.208967255	1.48622664	2.986721334	0.497611419	0.618757954	NA	Proteobacteria	Alphaproteobacteria	Caulobacterales	Caulobacteraceae	Phenylobacterium
Otu00590	0.12535857	0.795623241	2.981052251	0.266893423	0.78955122	NA	Proteobacteria	Alphaproteobacteria	unclassified	unclassified	unclassified
Otu00414	0.24904423	-0.031938878	2.973857123	-0.010739883	0.991430978	NA	Proteobacteria	Alphaproteobacteria	Rhizobiales	Hyphomicrobiaceae	Rhodomicrobium
Otu00752	0.22662902	-0.711480107	2.974063931	-0.239228249	0.810928599	NA	Proteobacteria	Alphaproteobacteria	Rhizobiales	Hyphomicrobiaceae	Rhodomicrobium
Otu00790	0.124982232	1.305276942	2.981319733	0.437818503	0.661517857	NA	Proteobacteria	Alphaproteobacteria	Rhizobiales	Hyphomicrobiaceae	Pedomicrobium
Otu00926	0.020170373	0.930278287	2.988578938	0.311277803	0.75558944	NA	Proteobacteria	Alphaproteobacteria	Rhizobiales	Hyphomicrobiaceae	Hyphomicrobium
Otu00959	0.082422226	1.37844855	2.984797751	0.4618231	0.644208184	NA	Proteobacteria	Alphaproteobacteria	Rhizobiales	Hyphomicrobiaceae	Pedomicrobium
Otu00755	0.08306861	0.969413403	2.981500691	0.325142773	0.745073018	NA	Proteobacteria	Alphaproteobacteria	Rhizobiales	DUNssu044	unclassified
Otu00404	0.299836238	-0.096654093	2.972165146	-0.032519759	0.974057559	NA	Proteobacteria	Alphaproteobacteria	Rhizobiales	Rhizobiaceae	Kaistia
Otu00571	0.122874083	1.80651987	2.984469929	0.605306776	0.54497518	NA	Proteobacteria	Alphaproteobacteria	Rhizobiales	Hyphomicrobiaceae	Devosia
Otu00583	0.120101065	0.789525849	2.97884149	0.265044599	0.790975105	NA	Proteobacteria	Alphaproteobacteria	Rhizobiales	Rhizobiales_Incertae_Sedi	Nordella
Otu01246	0.032439084	0.627076147	2.987078994	0.209929549	0.833722659	NA	Proteobacteria	Alphaproteobacteria	Rhizobiales	Rhizobiales_Incertae_Sedi	Nordella
Otu00517	0.239226705	1.790197038	2.979080056	0.60092277	0.547891427	NA	Proteobacteria	Alphaproteobacteria	Rhizobiales	unclassified	unclassified
Otu00638	0.153865599	-0.431173538	2.978285429	-0.144772403	0.884890572	NA	Proteobacteria	Alphaproteobacteria	Rhizobiales	unclassified	unclassified
Otu00954	0.049672009	0.94944518	2.984097467	0.318168287	0.750357287	NA	Proteobacteria	Alphaproteobacteria	Rhizobiales	unclassified	unclassified
Otu00966	0.234246363	1.805104051	2.984325707	0.60486161	0.545270953	NA	Proteobacteria	Alphaproteobacteria	Rhizobiales	Methylobacteriaceae	Microvirga
Otu00617	0.1365442	0.774753718	2.978412266	0.260123062	0.794768849	NA	Proteobacteria	Alphaproteobacteria	Rhizobiales	Methylobacteriaceae	Microvirga
Otu00741	0.08306861	0.969413403	2.981500691	0.325142773	0.745073018	NA	Proteobacteria	Alphaproteobacteria	Rhizobiales	unclassified	unclassified
Otu01368	0.227483482	1.277312043	2.984038612	0.428048095	0.668616105	NA	Proteobacteria	Alphaproteobacteria	Rhizobiales	unclassified	unclassified
Otu00660	0.090642194	0.705983776	2.980586317	0.236860705	0.812764856	NA	Proteobacteria	Alphaproteobacteria	Rhizobiales	Bradyrhizobiaceae	Bosea
Otu01325	0.096969637	1.20330139	2.986251218	0.402947141	0.686987111	NA	Proteobacteria	Alphaproteobacteria	Rhizobiales	Methylobacteriaceae	Methylobacterium
Otu00799	0.016895746	0.952748431	2.988584845	0.318795845	0.749881329	NA	Proteobacteria	Alphaproteobacteria	Rhizobiales	Methylobacteriaceae	Methylobacterium
Otu00713	0.16313929	1.89898728	2.983304829	0.636538131	0.524425736	NA	Proteobacteria	Alphaproteobacteria	Rhizobiales	unclassified	unclassified
Otu00402	0.267173844	0.031001907	2.968295715	0.010444346	0.991666769	NA	Proteobacteria	Alphaproteobacteria	Rhizobiales	unclassified	unclassified
Otu00453	0.246913902	1.080724166	2.976002234	0.363146288	0.716495608	NA	Proteobacteria	Alphaproteobacteria	Rhizobiales	Methylocystaceae	unclassified
Otu00635	0.185567834	-0.815883291	2.974124643	-0.274327202	0.783833182	NA	Proteobacteria	Alphaproteobacteria	Rhizobiales	unclassified	unclassified
Otu00664	0.180113868	1.238458325	2.980787986	0.415480179	0.677790422	NA	Proteobacteria	Alphaproteobacteria	Rhizobiales	Xanthobacteraceae	unclassified
Otu01134	0.176960215	1.203301393	2.986251218	0.402947142	0.68698711	NA	Proteobacteria	Alphaproteobacteria	Rhizobiales	Xanthobacteraceae	Pseudolabrys
Otu00894	0.059597784	0.455760821	2.98449586	0.152709483	0.878627382	NA	Proteobacteria	Alphaproteobacteria	Rhizobiales	Bradyrhizobiaceae	unclassified
Otu01171	0.221606809	1.663230955	2.98541781	0.55711832	0.577446591	NA	Proteobacteria	Alphaproteobacteria	Rhizobiales	unclassified	unclassified
Otu00444	0.266399611	1.176833043	2.974747735	0.395607678	0.692394472	NA	Proteobacteria	Alphaproteobacteria	Rhizobiales	Rhizobiales_Incertae_Sedi	Agaricicola
Otu00599	0.147758757	0.727199858	2.978560405	0.244144741	0.807118723	NA	Proteobacteria	Alphaproteobacteria	Rhizobiales	Rhizobiales_Incertae_Sedi	Agaricicola
Otu01000	0.035934706	1.417167828	2.98716826	0.474418481	0.635201517	NA	Proteobacteria	Alphaproteobacteria	Rhizobiales	Rhizobiales_Incertae_Sedi	Agaricicola

Otu00888	0.169883613	1.588561883	2.981164608	0.532866209	0.594126198	NA	Proteobacteria	Alphaproteobacteria	Rhizobiales	JG34-KF-361	unclassified
Otu00979	0.047256557	0.505603337	2.988224547	0.169198576	0.86564045	NA	Proteobacteria	Alphaproteobacteria	Rhizobiales	JG34-KF-361	unclassified
Otu00806	0.081953523	1.106786526	2.984095113	0.370895191	0.710715597	NA	Proteobacteria	Alphaproteobacteria	Rhodobacterales	Rhodobacteraceae	unclassified
Otu01179	0.022453958	0.874722073	2.986991784	0.292843816	0.769641535	NA	Proteobacteria	Alphaproteobacteria	unclassified	unclassified	unclassified
Otu00533	0.151117579	0.205052494	2.978145014	0.068852421	0.945107091	NA	Proteobacteria	Alphaproteobacteria	Rhodobacterales	Rhodobacteraceae	unclassified
Otu01094	0.227483482	1.277312043	2.984038612	0.428048095	0.668616105	NA	Proteobacteria	Alphaproteobacteria	Rhodobacterales	Rhodobacteraceae	unclassified
Otu00918	0.062581995	0.28864517	2.98568828	0.096676258	0.922983495	NA	Proteobacteria	Alphaproteobacteria	Rhodobacterales	Rhodobacteraceae	unclassified
Otu00860	0.064878169	0.17512066	2.983539145	0.058695613	0.953194553	NA	Proteobacteria	Alphaproteobacteria	Rhodobacterales	Rhodobacteraceae	Paracocccus
Otu00982	0.188976126	1.859090278	2.983979095	0.623023895	0.53326882	NA	Proteobacteria	Alphaproteobacteria	Caulobacterales	Hyphomonadaceae	Hirschia
Otu01269	0.052525718	0.585937944	2.986414272	0.19620116	0.84445272	NA	Proteobacteria	Alphaproteobacteria	Rhizobiales	Rhizobiales_Incertae_Sedi	Rhizomicrobium
Otu01304	0.070505569	0.619581692	2.986955691	0.207429154	0.835674716	NA	Proteobacteria	Alphaproteobacteria	Rhizobiales	Rhizobiales_Incertae_Sedi	Rhizomicrobium
Otu00762	0.103721297	1.563829512	2.98365197	0.524132683	0.600186258	NA	Proteobacteria	Alphaproteobacteria	Sphingomonadales	Sphingomonadaceae	Zymomonas
Otu00495	0.153771772	0.432103432	2.979076015	0.145046125	0.884674454	NA	Proteobacteria	Alphaproteobacteria	Sphingomonadales	unclassified	unclassified
Otu00957	0.092273343	1.293531447	2.985731084	0.43323776	0.664842049	NA	Proteobacteria	Alphaproteobacteria	Sphingomonadales	Sphingomonadaceae	unclassified
Otu00550	0.118171202	-0.400702108	2.978168254	-0.134546498	0.892970445	NA	Proteobacteria	Alphaproteobacteria	Sphingomonadales	Sphingomonadaceae	Sphingomonas
Otu00810	0.095844546	-0.213252947	2.980723383	-0.071544025	0.942964788	NA	Proteobacteria	Alphaproteobacteria	Sphingomonadales	unclassified	unclassified
Otu00562	0.133256301	0.300384914	2.979545985	0.100815666	0.91969679	NA	Proteobacteria	Alphaproteobacteria	Sphingomonadales	Sphingomonadaceae	unclassified
Otu00831	0.208806362	1.573306153	2.982614029	0.527492373	0.597851711	NA	Proteobacteria	Alphaproteobacteria	Sphingomonadales	Erythrobacteraceae	Porphyrobacter
Otu01330	0.055517327	0.727764691	2.989283964	0.243457865	0.807650722	NA	Planctomycetes	Planctomycetacia	Planctomycetales	Planctomycetaceae	unclassified
Otu00938	0.042699282	0.773389525	2.984557038	0.259130422	0.795534613	NA	Planctomycetes	Planctomycetacia	Planctomycetales	Planctomycetaceae	unclassified
Otu00682	0.094075017	0.413790074	2.980596604	0.138827936	0.889586118	NA	Planctomycetes	Planctomycetacia	Planctomycetales	Planctomycetaceae	Singulisphaera
Otu01158	0.023436115	0.951423453	2.98713087	0.318507456	0.75010004	NA	Planctomycetes	Planctomycetacia	Planctomycetales	Planctomycetaceae	Singulisphaera
Otu01262	0.066613295	0.559858742	2.988441231	0.187341392	0.851392971	NA	Planctomycetes	Planctomycetacia	Planctomycetales	Planctomycetaceae	Singulisphaera
Otu01015	0.02098545	1.374871053	2.987717143	0.460174436	0.645391018	NA	Planctomycetes	Planctomycetacia	Planctomycetales	Planctomycetaceae	Singulisphaera
Otu00428	0.275131809	-0.71885073	2.970358488	-0.242008072	0.808773909	NA	Planctomycetes	Planctomycetacia	Planctomycetales	Planctomycetaceae	Singulisphaera
Otu00532	0.214781081	1.721603221	2.979961648	0.577726637	0.563448693	NA	Planctomycetes	Planctomycetacia	Planctomycetales	Planctomycetaceae	Singulisphaera
Otu00847	0.104425843	1.405278534	2.983882978	0.470956316	0.637671931	NA	Planctomycetes	Planctomycetacia	Planctomycetales	Planctomycetaceae	Singulisphaera
Otu00825	0.293553575	0.51418548	2.982403435	0.172406414	0.86311803	NA	Planctomycetes	Planctomycetacia	Planctomycetales	Planctomycetaceae	Singulisphaera
Otu00845	0.075558789	0.504199059	2.981766661	0.169094069	0.86572265	NA	Planctomycetes	Planctomycetacia	Planctomycetales	Planctomycetaceae	Singulisphaera
Otu00474	0.186008059	0.368042971	2.976275708	0.123658897	0.901585357	NA	Planctomycetes	Planctomycetacia	Planctomycetales	Planctomycetaceae	Singulisphaera
Otu01164	0.031155782	1.113678553	2.986794892	0.372867436	0.709247109	NA	Planctomycetes	Planctomycetacia	Planctomycetales	Planctomycetaceae	Singulisphaera
Otu01052	0.088274856	0.462181452	2.98458424	0.154856226	0.876934664	NA	Planctomycetes	Planctomycetacia	Planctomycetales	Planctomycetaceae	Zavarzinella
Otu00796	0.16786254	1.804558289	2.984338883	0.604676064	0.545394255	NA	Planctomycetes	Planctomycetacia	Planctomycetales	Planctomycetaceae	Planctomyces
Otu00711	0.065764376	0.97006428	2.985273145	0.324949924	0.745218972	NA	Planctomycetes	Planctomycetacia	Planctomycetales	Planctomycetaceae	Planctomyces
Otu00984	0.242311173	0.91096243	2.985267306	0.305152717	0.760249842	NA	Planctomycetes	Planctomycetacia	Planctomycetales	Planctomycetaceae	Planctomyces
Otu01168	0.138687791	1.732570691	2.984835965	0.58045759	0.561606078	NA	Planctomycetes	Planctomycetacia	Planctomycetales	Planctomycetaceae	Planctomyces
Otu00398	0.243050621	0.235137055	2.974080675	0.079062097	0.936983231	NA	Planctomycetes	Planctomycetacia	Planctomycetales	Planctomycetaceae	Pir4_lineage
Otu00351	0.301587062	0.134117207	2.972329875	0.045121912	0.964010136	NA	Planctomycetes	Planctomycetacia	Planctomycetales	Planctomycetaceae	Pir4_lineage
Otu01085	0.037779395	0.741251105	2.984977935	0.248327164	0.803881283	NA	Planctomycetes	Planctomycetacia	Planctomycetales	Planctomycetaceae	Blastopirellula
Otu01192	0.107590428	1.203301382	2.986251218	0.402947138	0.686987113	NA	Planctomycetes	Planctomycetacia	Planctomycetales	Planctomycetaceae	Rhodopirellula
Otu00652	0.260149738	-0.956313403	2.971469861	-0.321831769	0.747580145	NA	Planctomycetes	Planctomycetacia	Planctomycetales	Planctomycetaceae	Pirellula
Otu01198	0.159089537	1.270221059	2.983937983	0.425686146	0.670336559	NA	Planctomycetes	Planctomycetacia	Planctomycetales	Planctomycetaceae	Pirellula
Otu00412	0.222703273	-0.203821816	2.97155375	-0.068590991	0.94531519	NA	Acidobacteria	Acidobacteria	Subgroup_6	unclassified	unclassified
Otu00372	0.299650642	-0.96331808	2.968284841	-0.32453694	0.74553156	NA	Acidobacteria	Acidobacteria	Subgroup_6	unclassified	unclassified
Otu01082	0.073825564	1.450986405	2.987032108	0.485761904	0.627135987	NA	Acidobacteria	Acidobacteria	Subgroup_6	unclassified	unclassified
Otu00700	0.152708327	-0.297326291	2.977689902	-0.099851328	0.920462358	NA	Acidobacteria	Acidobacteria	Subgroup_6	unclassified	unclassified
Otu01204	0.07292198	0.585937942	2.986414272	0.196201159	0.844452721	NA	Acidobacteria	Acidobacteria	Subgroup_6	unclassified	unclassified
Otu00803	0.134634424	-0.083996712	2.980137292	-0.028185518	0.977514188	NA	Acidobacteria	Acidobacteria	Subgroup_6	unclassified	unclassified
Otu00520	0.188071066	0.969064716	2.979143322	0.325283013	0.744966886	NA	Acidobacteria	Acidobacteria	Subgroup_4	Unknown_Family	Blastocatella
Otu00471	0.195027918	-0.185596021	2.975980368	-0.062364666	0.950272433	NA	Acidobacteria	Acidobacteria	Subgroup_4	Unknown_Family	Blastocatella
Otu00792	0.087011717	0.993580947	2.981841973	0.333210464	0.73897542	NA	Acidobacteria	Acidobacteria	Subgroup_4	RB41	unclassified
Otu00906	0.027066078	0.672575945	2.98715812	0.225155789	0.821858081	NA	unclassified	unclassified	unclassified	unclassified	unclassified
Otu01320	0.004274925	0.9713864	2.989547933	0.324927522	0.745235927	NA	Verrucomicrobia	Opitutae	Opitutales	Opitutaceae	Opitutus
Otu00526	0.279034516	-1.285333665	2.969947562	-0.432779919	0.665174663	NA	Verrucomicrobia	Spartobacteria	Chthoniobacterales	Xiphinematobacteraceae	Candidatus_Xiphinematobacter
Otu00644	0.102657563	-0.052709103	2.979625632	-0.017689841	0.985886285	NA	Verrucomicrobia	Spartobacteria	Chthoniobacterales	FukuN18_freshwater_grou	unclassified
Otu00521	0.159906538	1.351430676	2.98014907	0.453477542	0.650204911	NA	Verrucomicrobia	Spartobacteria	Chthoniobacterales	Xiphinematobacteraceae	Candidatus_Xiphinematobacter
Otu00760	0.070646973	0.093193759	2.982913746	0.031242525	0.975076126	NA	Verrucomicrobia	Spartobacteria	Chthoniobacterales	DA101_soil_group	unclassified
Otu00175	0.156386457	0.522666991	2.97869248	0.175468597	0.860711444	NA	Verrucomicrobia	Spartobacteria	Chthoniobacterales	DA101_soil_group	unclassified
Otu00677	0.089692223	0.711888051	2.980674169	0.238834576	0.811233858	NA	Verrucomicrobia	Spartobacteria	Chthoniobacterales	Chthoniobacteraceae	Chthoniobacter
Otu00944	0.07569309	1.759218808	2.984675457	0.589417119	0.555581496	NA	Verrucomicrobia	Spartobacteria	Chthoniobacterales	Chthoniobacteraceae	Chthoniobacter
Otu01213	0.191068772	1.203301391	2.986251218	0.402947141	0.686987111	NA	Verrucomicrobia	Verrucomicrobiae	Verrucomicrobiales	Verrucomicrobiaceae	Prosthecobacter
Otu00897	0.113836557	1.110848295	2.981880172	0.372532842	0.709496164	NA	Verrucomicrobia	Verrucomicrobiae	Verrucomicrobiales	Verrucomicrobiaceae	Luteolibacter
Otu00749	0.085087398	-0.067613151	2.981151593	-0.022680212	0.98190536	NA	Verrucomicrobia	Verrucomicrobiae	Verrucomicrobiales	Verrucomicrobiaceae	Haloferula
Otu00779	0.051242402	0.831707073	2.989467755	0.278212425	0.780849298	NA	Verrucomicrobia	Verrucomicrobiae	Verrucomicrobiales	Verrucomicrobiaceae	Luteolibacter
Otu01288	0.141768355	1.133841022	2.987068843	0.379583157	0.704254865	NA	Verrucomicrobia	OPB35_soil_group	unclassified	unclassified	unclassified
Otu00430	0.306999231	0.076758774	2.973741051	0.025812192	0.979407138	NA	Verrucomicrobia	OPB35_soil_group	unclassified	unclassified	unclassified
Otu00902	0.01248758	0.815608616	2.989168696	0.272854663	0.784964938	NA	Firmicutes	unclassified	unclassified	unclassified	unclassified
Otu00035	0.019422142	0.909547968	2.987568111	0.304444262	0.760789451	NA	Firmicutes	Negativicutes	Selenomonadales	Veillonellaceae	Veillonella
Otu00600	0.102800309	-0.291594624	2.97943662	-0.097869047	0.922036279	NA	unclassified	unclassified	unclassified	unclassified	unclassified
Otu00225	0.076917306	1.444750904	2.984590249	0.484070101	0.628336119	NA	Fusobacteria	Fusobacteriia	Fusobacteriales	Leptotrichiaceae	Streptobacillus
Otu00228	0.054248325	1.532599857	2.986439343	0.513186299	0.607821009	NA	Tenericutes	Mollicutes	Entomoplasmatales	Entomoplasmatales_Incer	Candidatus_Hepatoplasma
Otu00629	0.236142206	0.799475418	2.977643968	0.268492616	0.788320158	NA	Firmicutes	Erysipelotrichia	Erysipelotrichales	Erysipelotrichaceae	unclassified
Otu00508	0.19379078	-0.366647479	2.974765104	-0.123252582	0.901907089	NA	Armatimonadetes	unclassified	unclassified	unclassified	unclassified
Otu00507	0.19379078	-0.366647479	2.974765104	-0.123252582	0.901907089	NA	Armatimonadetes	unclassified	unclassified	unclassified	unclassified
Otu00165	0.089398975	1.687144104	2.985507786	0.565111273	0.571998082	NA	Firmicutes	Bacilli	unclassified	unclassified	unclassified
Otu00915	0.058967378	1.325067829	2.986776528	0.443644784	0.657299422	NA	Firmicutes	Bacilli	Lactobacillales	Enterococcaceae	Catellicoccus
Otu00896	0.073522174	1.305676898	2.985230965	0.437378854	0.661836617	NA	Firmicutes	Bacilli	Lactobacillales	Lactobacillaceae	Lactobacillus
Otu00819	0.106216365	1.10678654	2.984095113	0.370895195	0.710715594	NA	Firmicutes	Bacilli	Bacillales	Listeriaceae	Listeria
Otu00145	0.013922857	0.831707092	2.989467755	0.278212431	0.780849293	NA	Firmicutes	Bacilli	Bacillales	Staphylococcaceae	Macrococcus
Otu00226	0.099072544	0.061180678	2.98179084	0.020518099	0.983630075	NA	Firmicutes	Bacilli	Bacillales	Bacillaceae	Bacillus
Otu00603	0.1673978	1.150059665	2.978757096	0.386087092	0.699432169	NA	Firmicutes	Bacilli	Bacillales	unclassified	unclassified
Otu00765	0.259922567	0.833185048	2.981197918	0.279479951	0.779876522	NA	Firmicutes	Erysipelotrichia	Erysipelotrichales	Erysipelotrichaceae	Turicibacter
Otu00128	0.004172948	1.15858249	2.989162417	0.387594359	0.698316244	NA	Firmicutes	Bacilli	Lactobacillales	Leuconostocaceae	Weissella
Otu00757	0.0777283	0.78683606	2.98236975	0.263829145	0.791911576	NA	Firmicutes	Bacilli	Bacillales	Paenibacillaceae	Paenibacillus
Otu01277	0.033186024	0.619581693	2.986955691	0.207429154	0.835674716	NA	Firmicutes	Bacilli	Bacillales	Paenibacillaceae	Paenibacillus
Otu00675	0.086704464	0.726615675	2.980896041	0.243757469	0.807418661	NA	Firmicutes	Bacilli	Bacillales	Paenibacillaceae	Paenibacillus
Otu00490	0.156600634	-0.416475567	2.975923927	-0.139948324	0.88870082	NA	Firmicutes	Bacilli	Bacillales	Paenibacillaceae	Paenibacillus
Otu00775	0.129917762	-0.131610311	2.981435747	-0.044143266	0.964790205	NA	Firmicutes	Bacilli	Bacillales	Paenibacillaceae	Cohnella
Otu00837	0.22179203	1.105675861	2.985995421	0.370287192	0.711168516	NA	Actinobacteria	Coriobacteriia	Coriobacteriales	Coriobacteriaceae	unclassified
Otu01024	0.159089537	1.270221059	2.983937983	0.425686146	0.670336559	NA	Acidobacteria	Acidobacteria	Subgroup_3	unclassified	unclassified
Otu01265	0.069758629	0.627076146	2.987078994	0.209929549	0.833722659	NA	Acidobacteria	Acidobacteria	Subgroup_3	Unknown_Family	Candidatus_Solibacter
Otu00940	0.063834298	0.779692247	2.986580071	0.261065241	0.794042196	NA	Armatimonadetes	Armatimonadia	Armatimonadales	unclassified	unclassified
Otu00776	0.085493712	0.384581186	2.982925909	0.128927502	0.897415015	NA	Deinococcus-Thermus	Deinococci	Deinococcales	Trueperaceae	Truepera
Otu01146	0.139410687	1.133841009	2.987068844	0.379583153	0.704254869	NA	Deinococcus-Thermus	Deinococci	Deinococcales	Trueperaceae	Truepera
Otu00781	0.093429467	0.328670149	2.982720889	0.110191386	0.912257593	NA	Chloroflexi	TK10	unclassified	unclassified	unclassified
Otu00770	0.197615872	0.982060134	2.980188067	0.329529584	0.741755437	NA	unclassified	unclassified	unclassified	unclassified	unclassified
Otu00978	0.210331939	0.975869184	2.982988238	0.327144831	0.743558345	NA	Chloroflexi	Chloroflexia	Kallotenuales	AKIW781	unclassified
Otu00529	0.121039117	0.258543657	2.979253892	0.086781344	0.930845317	NA	Chloroflexi	Thermomicrobia	JG30-KF-CM45	unclassified	unclassified
Otu00563	0.146190571	1.002826933	2.979362768	0.33659108	0.736425181	NA	Chloroflexi	Thermomicrobia	JG30-KF-CM45	unclassified	unclassified
Otu00868	0.101282656	1.395432777	2.983043803	0.467788229	0.639936039	NA	Chloroflexi	Thermomicrobia	JG30-KF-CM45	unclassified	unclassified
Otu00459	0.304725783	0.789447481	2.973051075	0.26553445	0.790597775	NA	Chloroflexi	Thermomicrobia	JG30-KF-CM45	unclassified	unclassified
Otu01347	0.284277478	1.856281531	2.98390835	0.622097368	0.533877845	NA	Chloroflexi	KD4-96	unclassified	unclassified	unclassified
Otu01415	0.072901815	0.55985873	2.988441231	0.187341389	0.851392974	NA	Chloroflexi	Anaerolineae	Anaerolineales	Anaerolineaceae	unclassified
Otu01251	0.049382532	0.685928669	2.984227289	0.229851349	0.818207282	NA	Chloroflexi	Anaerolineae	Anaerolineales	Anaerolineaceae	unclassified
Otu01208	0.139410687	1.133841009	2.987068844	0.379583153	0.704254869	NA	Chloroflexi	Anaerolineae	Anaerolineales	Anaerolineaceae	unclassified
Otu01191	0.054457235	1.352244904	2.987094822	0.452695674	0.650767895	NA	Chloroflexi	Anaerolineae	Anaerolineales	Anaerolineaceae	unclassified
Otu00487	0.181694147	0.347663672	2.977033831	0.116781901	0.907032886	NA	Saccharibacteria	unclassified	unclassified	unclassified	unclassified
Otu01264	0.060006504	0.733450708	2.988758087	0.24540317	0.806144275	NA	Planctomycetes	Phycisphaerae	Phycisphaerales	Phycisphaeraceae	Phycisphaera
Otu01248	0.042174885	1.096946341	2.98739967	0.367191023	0.713476535	NA	Planctomycetes	Phycisphaerae	WD2101_soil_group	unclassified	unclassified
Otu00548	0.103683039	1.2377914	2.98302017	0.414945702	0.678181652	NA	Planctomycetes	Phycisphaerae	WD2101_soil_group	unclassified	unclassified
Otu00294	0.037918662	1.571320447	2.986046887	0.526220956	0.598734695	NA	Planctomycetes	Phycisphaerae	WD2101_soil_group	unclassified	unclassified
Otu00827	0.143407384	-0.128811409	2.981484055	-0.043203789	0.965539084	NA	Planctomycetes	Phycisphaerae	WD2101_soil_group	unclassified	unclassified
Otu01364	0.227483482	1.277312043	2.984038612	0.428048095	0.668616105	NA	unclassified	unclassified	unclassified	unclassified	unclassified
Otu00407	0.237810178	1.550571288	2.977608103	0.520743911	0.602545181	NA	unclassified	unclassified	unclassified	unclassified	unclassified
Otu00575	0.137346778	0.936428744	2.977958525	0.314453253	0.753176816	NA	unclassified	unclassified	unclassified	unclassified	unclassified
Otu00160	0.308663045	-0.142253483	2.973429388	-0.047841554	0.961842519	NA	Spirochaetae	Spirochaetes	Spirochaetales	Brachyspiraceae	Brachyspira
Otu00823	0.085493712	0.384581186	2.982925909	0.128927502	0.897415015	NA	Bacteroidetes	Cytophagia	Cytophagales	Cytophagaceae	Persicitalea

Otu00619	0.050743438	1.233138836	2.98589362	0.412988202	0.679615258	NA	Bacteroidetes	Cytophagia	Cytophagales	Cytophagaceae	Spirosoma
Otu00543	0.151803762	0.809043356	2.980300602	0.271463676	0.786034434	NA	Bacteroidetes	Cytophagia	Cytophagales	Cytophagaceae	Spirosoma
Otu00795	0.153146527	0.457749242	2.982449768	0.153480956	0.878019007	NA	Bacteroidetes	Cytophagia	Cytophagales	Cytophagaceae	Dyadobacter
Otu00580	0.17547917	1.246677596	2.981337614	0.418160489	0.675829781	NA	Bacteroidetes	Cytophagia	Cytophagales	Cytophagaceae	Hymenobacter
Otu00411	0.247329593	2.085810195	2.979632346	0.700022671	0.483913146	NA	Bacteroidetes	Cytophagia	Cytophagales	Cytophagaceae	Hymenobacter
Otu00739	0.29560491	0.149251472	2.97939896	0.05009449	0.96004709	NA	Bacteroidetes	Cytophagia	Cytophagales	Cytophagaceae	Hymenobacter
Otu00367	0.206647921	2.039928301	2.980545612	0.684414388	0.493713533	NA	Bacteroidetes	Sphingobacteriia	Sphingobacteriales	Sphingobacteriaceae	Mucilaginibacter
Otu00386	0.195817871	2.344822742	2.981131142	0.78655471	0.43154258	NA	Bacteroidetes	Sphingobacteriia	Sphingobacteriales	Sphingobacteriaceae	Mucilaginibacter
Otu01140	0.050315129	1.617851893	2.985666576	0.541872929	0.587906047	NA	Bacteroidetes	Sphingobacteriia	Sphingobacteriales	Sphingobacteriaceae	Pedobacter
Otu01376	0.260209656	1.203301394	2.986251218	0.402947142	0.68698711	NA	Bacteroidetes	Flavobacteriia	Flavobacteriales	NS9_marine_group	unclassified
Otu00066	0.055177619	1.528511448	2.986463812	0.511813149	0.608781787	NA	Bacteroidetes	Flavobacteriia	Flavobacteriales	Flavobacteriaceae	Riemerella
Otu01067	0.101761907	1.133841008	2.987068844	0.379583152	0.704254869	NA	Bacteroidetes	Flavobacteriia	Flavobacteriales	Flavobacteriaceae	Arenibacter
Otu00640	0.025547574	0.664459202	2.988684687	0.22232496	0.824060923	NA	Bacteroidetes	Flavobacteriia	Flavobacteriales	Flavobacteriaceae	Flavobacterium
Otu00801	0.139116968	1.355701358	2.983621217	0.454381189	0.649554489	NA	Bacteroidetes	Flavobacteriia	Flavobacteriales	Flavobacteriaceae	Ornithobacterium
Otu00429	0.229102008	1.128042264	2.977604004	0.378842271	0.704804995	NA	Bacteroidetes	Sphingobacteriia	Sphingobacteriales	Chitinophagaceae	Chitinophaga
Otu00967	0.062690355	1.293531459	2.985731084	0.433237764	0.664842046	NA	Bacteroidetes	Sphingobacteriia	Sphingobacteriales	Chitinophagaceae	Parafilimonas
Otu00880	0.077998512	1.491652452	2.986675722	0.499435691	0.617472481	NA	Bacteroidetes	Sphingobacteriia	Sphingobacteriales	Chitinophagaceae	unclassified
Otu01080	0.069758629	0.627076146	2.987078994	0.209929549	0.833722659	NA	Bacteroidetes	Sphingobacteriia	Sphingobacteriales	Chitinophagaceae	Flavisolibacter
Otu00579	0.061425246	1.595535592	2.984428632	0.53462012	0.592912568	NA	Bacteroidetes	Sphingobacteriia	Sphingobacteriales	Chitinophagaceae	unclassified
Otu00282	0.254724054	2.001341273	2.978629956	0.671899935	0.501647402	NA	Bacteroidetes	Sphingobacteriia	Sphingobacteriales	Chitinophagaceae	unclassified
Otu00427	0.221100738	0.245313104	2.976272201	0.08242294	0.934310395	NA	Bacteroidetes	Sphingobacteriia	Sphingobacteriales	Chitinophagaceae	unclassified
Otu00930	0.170974819	1.82332554	2.984213464	0.61099032	0.541205989	NA	Bacteroidetes	Sphingobacteriia	Sphingobacteriales	Chitinophagaceae	Ferruginibacter
Otu00489	0.231762287	-1.04152651	2.971996831	-0.350446709	0.726003477	NA	Bacteroidetes	Sphingobacteriia	Sphingobacteriales	Chitinophagaceae	Segetibacter
Otu01243	0.070684709	0.678209454	2.984126218	0.227272375	0.820211952	NA	Bacteroidetes	Sphingobacteriia	Sphingobacteriales	Chitinophagaceae	Taibaiella
Otu01036	0.10103901	1.732570931	2.984835963	0.580457671	0.561606024	NA	Bacteroidetes	Sphingobacteriia	Sphingobacteriales	env.OPS_17	unclassified
Otu01043	0.313573611	1.6540443	2.983924584	0.5543184	0.57936096	NA	Bacteroidetes	Bacteroidia	Bacteroidales	Prevotellaceae	Prevotella_9
Otu00011	0.231890811	1.140688686	2.978888399	0.382924277	0.70177591	NA	Bacteroidetes	Bacteroidia	Bacteroidales	Bacteroidaceae	Bacteroides
Otu01335	0.238263388	1.272810243	2.987212891	0.426086218	0.670045022	NA	Bacteroidetes	Cytophagia	Cytophagales	Cytophagaceae	unclassified
Otu01101	0.03793837	0.740093362	2.984961744	0.247940652	0.804180326	NA	Bacteroidetes	Cytophagia	Cytophagales	Cytophagaceae	Chryseolinea
Otu01064	0.060006504	0.733450708	2.988758087	0.24540317	0.806144275	NA	Bacteroidetes	Cytophagia	Cytophagales	Cytophagaceae	Ohtaekwangia
Otu00501	0.191791537	-0.799191611	2.973666611	-0.268756292	0.78811723	NA	Bacteroidetes	Cytophagia	Cytophagales	Cytophagaceae	unclassified
Otu00889	0.241028952	1.453720361	2.983224864	0.487298285	0.626046963	NA	Bacteroidetes	Cytophagia	Cytophagales	Cytophagaceae	unclassified
Otu00697	0.255215749	1.682100762	2.982939713	0.563907059	0.572817386	NA	Bacteroidetes	Cytophagia	Cytophagales	Cytophagaceae	unclassified
Otu01169	0.016812502	1.33555333	2.988061427	0.446963144	0.654901671	NA	Bacteroidetes	Cytophagia	Cytophagales	Cytophagaceae	Adhaeribacter
Otu00258	0.080349134	-0.022047031	2.981740698	-0.007394014	0.994100485	NA	Bacteroidetes	Cytophagia	Order_II	Rhodothermaceae	Rubrivirga
