# Supplementary material for: Glucocorticoids modulate gastrointestinal microbiome in a wild bird
Source: R Soc Open Sci. 2018 Apr 18;5(4):171743. doi: 10.1098/rsos.171743 (PMC5936907; doi:10.1098/rsos.171743)
Supplement: Supplemetary Materia [file rsos171743supp3.docx]

**Royal Society Open science**

Noguera et al. ‘**GLUCOCORTICOIDS MODULATE GASTROINTESTINAL MICROBIOME IN A WILD BIRD’**

SUPPLEMENTARY MATERIAL (SM)


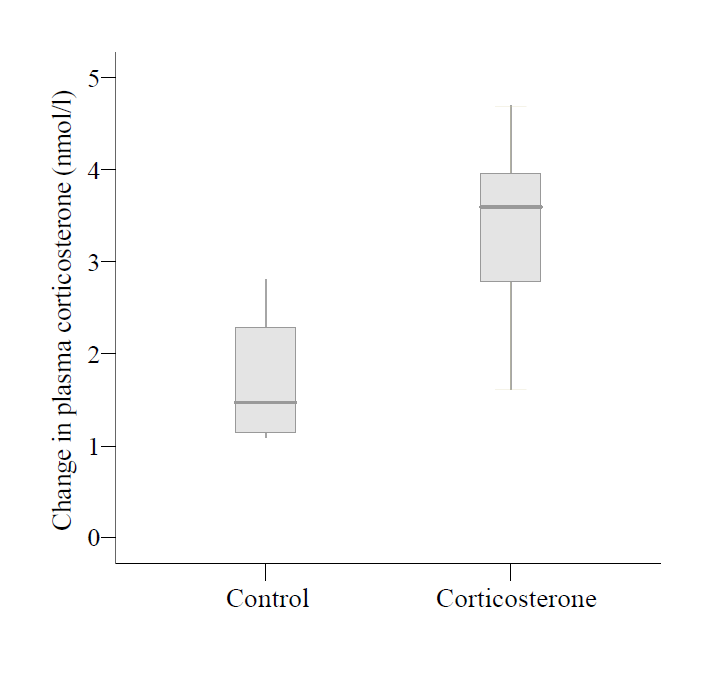


**Figure S1.** Change in plasma corticosterone levels (squared root-transformed) during the first 8 days after hatching in sham-implanted (control) and corticosterone-implanted (corticosterone) yellow-legged gull chicks. The experimental treatment successfully increased plasma corticosterone levels within the normal range of variation in this colony (corticosterone group: range, 1.44-9.33 nmol/L; natural population: 2.66-18.5nmol/L).

**Figure S2**. Rarefaction curves indicating the observed number of operational taxonomic units (OTUs) at a genetic distance of 3% in cloacal samples of control (red) and corticosterone chicks (blue)

**Figure S3**. Taxa prevalence versus total counts. Each point is a different taxon. A prevalence threshold of 5% of all samples was selected.

**Figure S4.** Effects of corticosterone implants on diversity of cloacal microbiome of gull chicks. (a) Observed OTU richness, (b) estimated taxonomic richness (Chao 1), and (d) Faith’s phylogenetic diversity (PD).


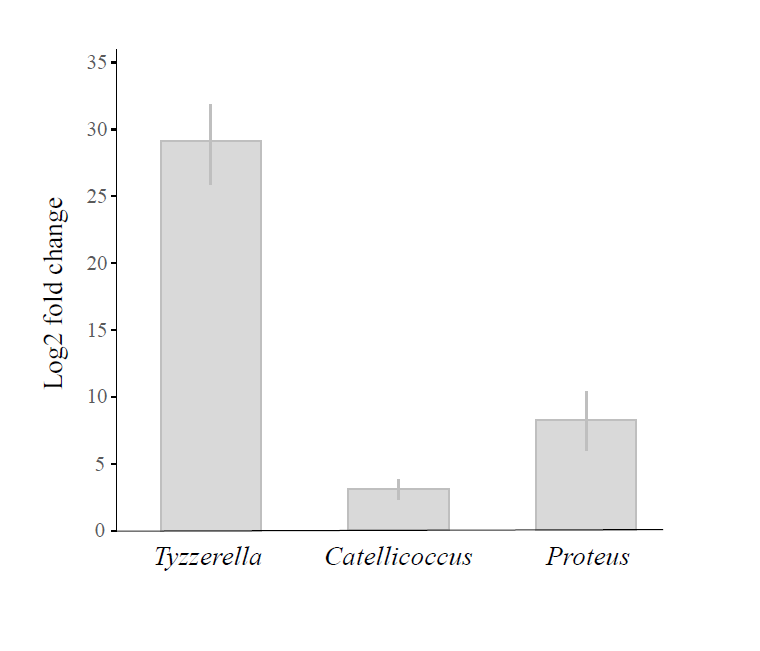


**Figure S5**. Bacterial genera more abundant (P-adj <0.001; see table S2 for further statistical details) in male than in female chicks. Bars represent the change in the abundance as Log2 fold change (mean +-SE).

**Figure S6**. Box-plot of PC1 extracted from the normalised abundance of OTUs in control and corticosterone chicks.


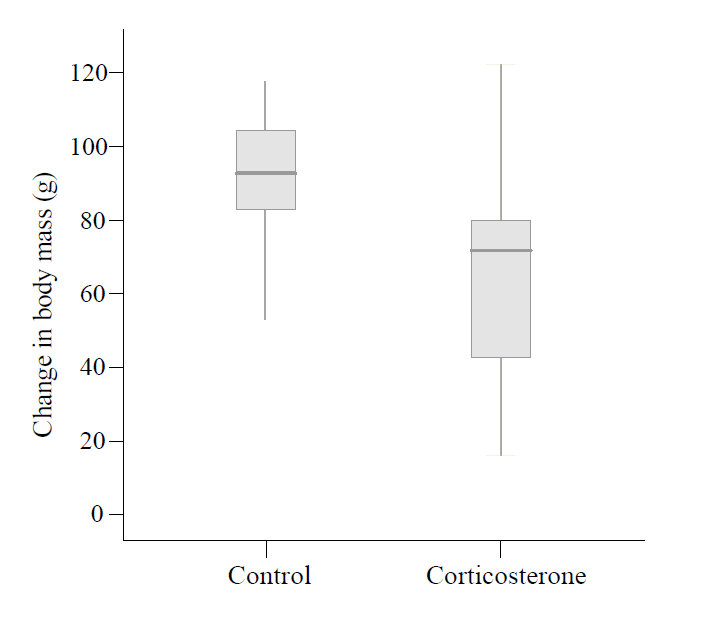


**Figure S6.** Change in body mass during the first 8 days after hatching in sham-implanted (control) and corticosterone-implanted (corticosterone) yellow-legged gull chicks. Similar results were achieved when the same model was tested on size-corrected body mass i.e., residuals of body mass regressed on tarsus length (treatment: F_1,27_=2.659, p=0.115; Age: F_1,27_=0.080, p=0.775; treatment x age; F_1,27_=4.541, p=0.042).
